# A comparison of strategies for selecting auxiliary variables for multiple imputation

**DOI:** 10.1002/bimj.202200291

**Published:** 2024-01-23

**Authors:** Rheanna M. Mainzer, Cattram D. Nguyen, John B. Carlin, Margarita Moreno‐Betancur, Ian R. White, Katherine J. Lee

**Affiliations:** ^1^ Clinical Epidemiology and Biostatistics Unit Murdoch Children's Research Institute Parkville Victoria Australia; ^2^ Department of Paediatrics The University of Melbourne Parkville Victoria Australia; ^3^ Centre for Epidemiology and Biostatistics, Melbourne School of Population and Global Health The University of Melbourne Parkville Victoria Australia; ^4^ MRC Clinical Trials Unit University College London London UK

**Keywords:** imputation model, missing data, variable selection

## Abstract

Multiple imputation (MI) is a popular method for handling missing data. Auxiliary variables can be added to the imputation model(s) to improve MI estimates. However, the choice of which auxiliary variables to include is not always straightforward. Several data‐driven auxiliary variable selection strategies have been proposed, but there has been limited evaluation of their performance. Using a simulation study we evaluated the performance of eight auxiliary variable selection strategies: (1, 2) two versions of selection based on correlations in the observed data; (3) selection using hypothesis tests of the “missing completely at random” assumption; (4) replacing auxiliary variables with their principal components; (5, 6) forward and forward stepwise selection; (7) forward selection based on the estimated fraction of missing information; and (8) selection via the least absolute shrinkage and selection operator (LASSO). A complete case analysis and an MI analysis using all auxiliary variables (the “full model”) were included for comparison. We also applied all strategies to a motivating case study. The full model outperformed all auxiliary variable selection strategies in the simulation study, with the LASSO strategy the best performing auxiliary variable selection strategy overall. All MI analysis strategies that we were able to apply to the case study led to similar estimates, although computational time was substantially reduced when variable selection was employed. This study provides further support for adopting an inclusive auxiliary variable strategy where possible. Auxiliary variable selection using the LASSO may be a promising alternative when the full model fails or is too burdensome.

## 1 INTRODUCTION

Missing data are often encountered in medical research. Many studies aim to estimate an expected value (e.g., a mean or proportion) or an exposure–outcome association. Restricting the analysis to individuals with available data, that is, analyzing complete cases, can lead to bias or loss of precision in these estimates compared to if all data were observed (Little & Rubin, 2002). Multiple imputation (MI) is a popular two‐stage method for handling missing data that can produce valid estimates and standard errors (SEs) of target quantities under relaxed assumptions regarding the mechanism leading to missing data (Rubin, 2004; van Buuren, 2018). In the first stage of MI, missing data are imputed multiple times with random draws from the predictive distribution of the missing values given the observed data and a specified imputation model. In the second stage, the statistical analysis of interest is applied to each imputed data set and the results are combined using Rubin's rules to obtain a single estimate with associated SE (Rubin, 2004). When and how MI should be applied depends on the target of analysis, the reasons for missing data and whether incomplete records are informative about the target parameter.

MI is usually carried out using either multiple imputation by chained equations (MICE) or multivariate normal imputation (MVNI). MICE, also known as “fully conditional specification,” “regression switching,” or “sequential regression multiple imputation,” is a flexible MI approach in which univariate imputation models are specified for each variable with missing data (White et al., 2011). Imputed values for each variable with missing data are then generated using these models, one variable at a time, until all missing values are replaced (one cycle). The algorithm carries out a number of cycles before obtaining one imputed data set, and then this procedure is repeated *m* times to obtain *m* sets of complete data that differ in their imputed values. The second approach, MVNI, is based on the assumption that all incomplete variables jointly follow a multivariate normal distribution. Imputations are obtained from this model using the Data Augmentation algorithm (Schafer, 1997).

Although the availability of MI procedures in statistical software such as R, Stata and SAS has made MI widely accessible and easy to implement in practice, the user still needs to make a number of decisions in order to carry out an analysis using MI, including which variables to include in the imputation model and in what form (van Buuren et al., 1999). Best practice is to include all variables that appear in the main analysis, in the same form, as predictors in the imputation model (Meng, 1994). Failure to do so could introduce bias in MI estimates. A key benefit of MI is that additional variables that do not appear in the main analysis, known as “auxiliary variables,” can be added to the imputation model to improve performance by reducing bias and/or increasing precision (Collins et al., 2001; Schafer, 2003). Candidate auxiliary variables are variables that are related to the variables with missing data and possibly also related to the missingness of the variables with missing data (Schafer, 1997). The extent to which auxiliary variables reduce bias or improve precision in MI estimates depends on the missingness mechanism, the estimand and the relationship between the auxiliary variables and the variables with missing values (Collins et al., 2001; Hardt et al., 2012).

The choice of which auxiliary variables to include in the imputation model is not always straightforward. Traditionally the recommendation has been to use an inclusive auxiliary variable selection strategy, that is, to include more auxiliary variables rather than fewer (Collins et al., 2001). This advice is particularly pertinent when the imputer and analyst are different people because the imputer must accommodate a wide range of potential analyses (Rubin, 1996). However, with the increase in computing power and availability of software, it is easier now for the same person to perform both jobs, allowing each analysis to have a tailored MI procedure (i.e., the “impute once, analyze once” approach; Graham, 2012). Furthermore, the sheer size of modern data sets means there are often many potential auxiliary variables available to choose from. For example, the Longitudinal Study of Australian Children (LSAC) is an ongoing, large‐scale longitudinal study that collects data on hundreds of variables at each wave (currently up to wave 9, Sanson et al., 2002). While it has been recommended that imputations should not be created using a model that is too restrictive (van Buuren, 2018), including too many auxiliary variables in an imputation model can be problematic (Carpenter & Kenward, 2008; Hardt et al., 2012).

There are two main issues with adopting the inclusive auxiliary variable strategy. First, imputation algorithms may automatically drop variables or fail completely due to numerical problems such as perfect prediction, where categories of the response variable are perfectly separated by a linear combination of the covariates, or collinearity, where covariates are highly correlated (Nguyen et al., 2021; White et al., 2010). With many auxiliary variables included in the imputation procedure, encountering these types of computational issues are more likely. For example, Nguyen et al. (2021) considered different approaches to estimate an exposure–outcome association using MI when the exposure, covariates, and composite outcome all contained missing values. Of the 11 imputation model specifications they considered, 7 failed to generate imputations due to perfect prediction (3), collinearity (1), or convergence failure (3). Second, including too many auxiliary variables in the imputation model may have a detrimental effect on the properties of MI estimates. Hardt et al. (2012) found that including too many auxiliary variables increased bias and reduced precision of MI estimates, which led to the recommendation that the number of variables included in the imputation model should be no more than a third of the number of cases with complete data. After a sufficient number of suitable auxiliary variables have been included in the imputation model, the benefits of adding more may be small (Graham, 2012). For example, van Buuren et al. (1999) suggest that no more than 15–25 variables are needed in the imputation model in practice. Unlike the recommendation provided by Hardt et al. (2012) who consider limiting the number of auxiliary variables based on the sample size, van Buuren et al. (1999) justify their suggestion based on diminishing returns in the increase in explained variance from linear regression after including additional variables. These two issues motivate the desire to employ an auxiliary variable selection strategy when there are a large number of potential auxiliary variables available.

The aim of this study was to provide a broad comparison of a range of strategies for selecting the set of auxiliary variables for the imputation model in MI. Using a simulation study, we evaluated eight auxiliary variable selection strategies against two “benchmark” analysis strategies that do not use any auxiliary variable selection: a complete case analysis and MI using all auxiliary variables (the “full” imputation model). We also illustrate the implication of the method choice in practice by applying each analysis strategy to the motivating LSAC case study. In Section 2, we describe the motivating case study. In Section 3, we describe the auxiliary variable selection strategies that were evaluated. In Section 4, we describe the design of the simulation study. Results are provided in Section 5 and the strategies are applied to the motivating case study in Section 6. A discussion of these results and a summary of our key messages are provided in Section 7.

## 2 MOTIVATING EXAMPLE: THE LONGITUDINAL STUDY OF AUSTRALIAN CHILDREN

The LSAC is a large‐scale longitudinal study, with the aim of investigating the effect of a child's environment on their well‐being throughout life (Sanson et al., 2002). Two cohorts were recruited into the study in 2003: the baby “B” cohort, consisting of participants aged 0–1 years at wave 1, and the kindergarten “K” cohort, consisting of participants aged 4–5 at wave 1. This case study uses data from the 4983 children in the K cohort of LSAC to examine the association between body mass index (BMI) *z*‐score at 4–5 years of age and health‐related quality of life (HRQoL) problems at 12 and 13 years of age. This case study is a simplified version of a regression analysis that appears in Jansen et al. (2013).

The analysis of interest aimed to estimate the effect of BMI *z*‐score on HRQoL using a linear regression model with adjustment for potential confounders. The outcome of interest, HRQoL, was measured using the Total Scale Score of the Pediatric Quality of Life Inventory (PedsQL) collected at wave 4 (Varni et al., 2003). The PedsQL scale is made up of either 21 or 23 items (depending on the child's age), each of which is measured on a 5‐point Likert scale with 1 corresponding to “never a problem” and 5 corresponding to “always a problem.” The Total Scale Score is calculated as the average of the observed scale items, after a reverse linear transformation of items in the following manner: 1=100,2=75,3=50,4=25, and 5 = 0. The exposure was BMI *z*‐score at wave 1. BMI at each wave was calculated using height and weight measurements taken by an interviewer. These measurements were standardized by age and sex to derive the BMI *z*‐score. Several potentially confounding covariates were measured at wave 1: child sex, child age, whether the child was Aboriginal or Torres Strait Islander, whether the child had a non‐English speaking background and the socioeconomic status of the child's family.

The parameter of interest was the coefficient of BMI*z* in the following linear regression model, adjusted for potential confounders:

(1) $$E\left(\mathrm{HRQoL}\right)=\beta_{0}+\beta_{1}\mathrm{BMI}z+\beta_{2}\mathrm{Female}+\beta_{3}\mathrm{Age}+\beta_{4}\mathrm{IndStat}+\beta_{5}\mathrm{NonEng}+\beta_{6}\mathrm{SES},$$

where Female is an indicator for whether the child is female, Age is the age in months for the child at wave 1, IndStat is an indicator for whether the child is Aboriginal or Torres Strait Islander, NonEng is an indicator for whether the child has a non‐English speaking background, and SES is a standardized score representing the socioeconomic status of the child's family at wave 1. HRQoL was missing for 17.4% of participants. A small percentage of participants were also missing values for BMI*z*, IndStat, NonEng, and SES (Supporting Information Table 1). We have shown previously that individuals with missing data on one or more variables in the analysis model had lower mean HRQoL and SES, and were more likely to be Aboriginal or Torres Strait Islander or come from a non‐English speaking background than individuals with complete data on all variables (Mainzer et al., 2021). Furthermore, LSAC collects data on hundreds of variables at each wave, resulting in a large number of potential auxiliary variables available in the data set. The assumption that nonresponse in HRQoL does not depend on HRQoL itself after conditioning on variables in the imputation model is more plausible when the conditioning set includes auxiliary variables, and even more so if there are a large number of these. Thus, MI was identified as a more appropriate method of estimating β_1_ than a complete case analysis, which conditions only on variables in the analysis model. We note that it may be the case that missingness in HRQoL depended on HRQoL even after conditioning on analysis and auxiliary variables, resulting in the data being “missing not at random,” but this would imply the need for sensitivity analysis under specific substantive assumptions (Moreno‐Betancur et al., 2018). We do not consider this further.

A subset of 85 auxiliary variables for inclusion in the imputation model was initially identified across the four waves of data collection based on substantive knowledge. This subset was the PedsQL scale items (measured at waves 1–3; 67 ordinal items in total); Global Health Measure (GHM, measured at waves 1–4), a rating of the child's current health; special health care needs (measured at waves 1–4), a variable that indicates whether the child has a health condition that requires special care; Strengths and Difficulties Questionnaire (SDQ, measured at waves 1–4), a scale score providing information on the child's behavior; Matrix Reasoning Test (measured at waves 2–4), a measure of the nonverbal intelligence of the child; and the Peabody Picture Vocabulary Test (measured at waves 1–3), a scale score providing information on the child's vocabulary. The case study had two major complexities that were not incorporated into the design of the simulation study (Section 4). First, the exposure, two covariates and all auxiliary variables had some missing values. Second, the case study involved different types of variables (including highly skewed ordinal PedsQL items). Further details of the variables in this case study, including percentage of missing data, are provided in Supporting Information Table 1.

## 3 AVAILABLE AUXILIARY VARIABLE SELECTION STRATEGIES

Several strategies have been proposed to specify an imputation model for an incomplete variable using a reduced set of auxiliary variables prior to performing the imputation. van Buuren et al. (1999) use the following four‐step strategy for quick, data‐based selection of variables to be included in the imputation model for the incomplete variable. First, include all variables that appear in the analysis model. Then add variables that are related to the nonresponse in the incomplete variable according to a suitable criterion, such as the level of correlation between the variables undergoing selection and the missingness indicator of the incomplete variable. Next, add variables that explain a considerable amount of variance in the incomplete variable. Lastly, remove any variables in steps two and three that have too many missing values. Variables in steps two and three can be chosen based on their correlation with the missingness indicator of the incomplete variable and their correlation with the incomplete variable, respectively, while variables in step four can be identified by a criterion such as the proportion of usable cases (i.e., the proportion of observed cases in the variable being selected within the subset of cases where the incomplete variable is unobserved). This strategy has been implemented in the quickpred function in the R package mice to specify imputation models for each incomplete variable in the MICE procedure (van Buuren & Groothuis‐Oudshoorn, 2011), and has been adopted in practice to choose an imputation model when the full model is not feasible (Clark & Altman, 2003; Heymans et al., 2007). A similar strategy is given by Graham (2012), with guidance on correlation cutoffs for inclusion of variables (0.4 if the variable is a cause of missingness, 0.5 otherwise, p. 197) but it is noted that these can be reduced to include more variables if there are a relatively small number of variables in the analysis.

In a different approach, Howard et al. (2015) suggest using principal components of auxiliary variables in the imputation model, instead of the auxiliary variables themselves. Strictly speaking, this approach is a dimensionality reduction method rather than a variable selection method, but we included it in this comparison since it has also been adopted in practice to specify the imputation model (Erentaitė et al., 2018; Jensen et al., 2018; Jensen & Orsmond, 2019; Latzman et al., 2015; Metzger et al., 2018; Nair et al., 2018; Roche et al., 2019), with the R package PcAux available to aid its implementation (Lang et al., 2017).

Other strategies for selecting auxiliary variables utilize hypothesis tests or penalized regression. The ice package in Stata has an option to construct imputation models for each incomplete variable using stepwise selection (Royston & White, 2011). An additional step is added between the initialization of the MICE algorithm (where one imputation is performed to obtain a complete data set) and performing the imputation, in which the stepwise command is used on the complete data set to select variables for each of the univariate imputation models. A related approach proposed by Andridge and Thompson (2015) uses forward selection with the fraction of missing information (FMI) due to nonresponse (estimated as the ratio of between‐imputation variance to total variance for a given estimator; Little & Rubin, 2002) for the mean of the incomplete variable as the selection criterion for inclusion of auxiliary variables in the imputation model for that variable. Dixon and Brown (1983) described the implementation of two‐sample *t*‐tests to identify other variables that are associated with missingness in the incomplete variable. The sample is split into two groups based on the presence or absence of values of the incomplete variable. *t*‐Tests are then applied to the other variables using this grouping, with large test statistics providing evidence that the probability of being missing is not the same for all cases, that is, data are not missing completely at random (MCAR, Rubin, 1976). Enders (2010) suggests results from such *t*‐tests can be used to identify, and limit the number of, potential auxiliary variables. Another approach is to use the least absolute shrinkage and selection operator (LASSO), which simultaneously performs variable regularization and selection, to select auxiliary variables for the imputation model (James et al., 2021). This approach has been used for dimension reduction in the context of high‐dimensional data (Zhao & Long, 2016).

In this paper, we evaluate the performance of the following strategies (described in detail in Section 4.4): (1, 2) two versions of the four‐step strategy used by van Buuren et al. (1999) (2018); (3) selection of predictors of missingness in the incomplete variable using hypothesis tests of the MCAR assumption; (4) replacing auxiliary variables with a subset of their principal components; (5, 6) forward and forward stepwise selection of predictors of the incomplete variable; (7) forward selection based on the FMI; and (8) selection via the LASSO.

## 4 SIMULATION STUDY

A simulation study was conducted to evaluate the performance of the above strategies for selecting auxiliary variables for inclusion in the imputation model for an incompletely observed variable when estimating a marginal mean or an exposure–outcome association. For simplicity, we focused on the setting where there is one incompletely observed continuous outcome, one completely observed continuous exposure, one completely observed confounding variable, and a set of completely observed continuous auxiliary variables.

### 4.1 Simulation of complete data

Let *X* denote a complete continuous exposure variable, *Y* denote an incomplete continuous outcome, *Z* denote a complete continuous covariate and $A={\left(A_{1},A_{2},\cdots ,A_{p}\right)}^{⊤}$ denote a *p*‐vector of continuous complete potential auxiliary variables. Also let ***0***_*c*_ denote a *c*‐dimensional vector of 0s. We considered sample sizes of n∈{250,1000} to reflect moderate and large studies, combined factorially with the ratio of sample size to number of auxiliary variables n:p∈{10:1,3:1}, resulting in four combinations: (i) n=250 and p=25, (ii) n=250 and p=83, (iii) n=1000 and p=100, and (iv) n=1000 and p=333.

Data for ${\left(Y,X,Z,A^{⊤}\right)}^{⊤}$ were generated from a multivariate normal distribution with mean $0_{p+3}$ and correlation matrix ***Σ***. Since the strength of relationship between an auxiliary variable and a variable with missing data is a key consideration for inclusion in the imputation model (Graham, 2012; Hardt et al., 2012; van Buuren, 2018), we designed ***Σ*** such that there was a range of realistic correlations between *Y* and each auxiliary variable. This was done by assigning auxiliary variables into one of three groups, where variables in group one had moderate correlations with *Y* (0.4 on average), variables in group two had low correlations with *Y* (0.2 on average) and variables in group three had very low correlations with *Y* (0.1 on average). In particular, the requirement for ***Σ*** to be positive semidefinite meant that we could not consider too many auxiliary variables that were highly correlated with *Y*. However, very large correlations were not observed in the case study (Supporting Information Figure 1). We also designed ***Σ*** to have (i) 80% power to reject the null hypothesis that the coefficient of *X*, $\beta_{X}$, in the analysis model (see Equation 2) is equal to 0 at the 5% level of significance; and (ii) a similar correlation structure across each of the different scenarios. The form and construction of ***Σ*** are described in detail in Supporting Information Section 2.1.

We generated 2000 data sets so that the Monte Carlo SE for the estimated coverage probability of the 95% confidence intervals for our estimands (see Section 4.3) was less than 0.5%. Data sets were generated in R using mvrnorm, which utilizes an eigenvalue decomposition of the covariance matrix.

### 4.2 Imposing missing data

After generating the complete data, values of *Y* were set to missing with a probability determined by the logistic regression model

$$\text{logit}\;P\left(M_{Y}=1\right)=\gamma_{0}+\gamma_{X}X+\gamma_{Z}Z+{\gamma_{A}}^{⊤}A,$$

where $M_{Y}$ denotes the missingness indicator for *Y*, and ${\left(\gamma_{0},\gamma_{X},\gamma_{Z},{\gamma_{A}}^{⊤}\right)}^{⊤}\in R^{p+3}$ determines the proportion of missing data, which variables are related to $M_{Y}$ and the strength of the missingness mechanism.

For each combination of *n* and n:p, the following missingness mechanism was used. Values of *Y* had a 30% chance of being missing and the odds of missingness increased by 20% with every 1 standard deviation increase in each of *X*, *Z* and a set of auxiliary variables chosen to be associated with missingness, conditional on the other variables associated with missingness. This was achieved by setting $\gamma_{X}=\gamma_{Z}=\log\left(1.2\right)$ and the elements of ***γ_A_*** to either 0 or log (1.2). For the scenario where n=1000 and p=100 (the scenario closest to the case study), we additionally examined (i) the impact of increasing the proportion of missing data in *Y* from 30% to 50% and (ii) the impact of increasing the conditional odds of missingness from a 20% increase to a 100% increase. Since three different groups of auxiliary variables were considered, in all scenarios we set ***γ_A_*** such that half of the auxiliary variables in each group were associated with $M_{Y}$. In terms of Rubin's missingness mechanisms, *Y* is “missing at random.” More precisely, missingness is independent of *Y* given *X*, *Z*, and ***A***. The strength of the missingness mechanism was chosen based on what was observed in the LSAC case study. Estimated odds ratios were obtained from univariate logistic regressions of the missingness indicator for HRQoL on all other variables in the case study, which were rescaled to have mean 0 and variance 1. These odds ratios ranged from 0.6 to 1.3 (Supporting Information Table 1 and Supporting Information Figure 2). A value of log (1.2) was chosen for the missingness model coefficients based on the distribution of the odds ratios. For simplicity, the strength of association with missingness was chosen to be same for all variables associated with missingness. The missingness probabilities were chosen to moderately large, but realistic. In Supporting Information Section 2.2, we describe how γ_0_ was calculated to achieve the desired proportions of missingness in *Y*.

### 4.3 Estimands

We considered two estimands: the regression coefficient of *X* on *Y*, adjusted for *Z*, that is, the parameter $\beta_{X}$ in the following linear regression model:

(2) $$E\left(Y\;|\;X,Z\right)=\beta_{0}+\beta_{X}X+\beta_{Z}Z,$$

and the marginal mean of *Y*, $\mu_{Y}=E\left(Y\right)$. The true value of $\mu_{Y}=0$ was known from the data generation and the true value of $\beta_{X}$ was calculated for each scenario using conditional distribution properties of the multivariate normal distribution ($\beta_{X}=0.18$ for the scenarios where n=250,p=25 and n=250,p=83, and $\beta_{X}=0.09$ for the scenarios where n=1000,p=100 and n=1000,p=333).

The motivation for using MI rather than a complete case analysis may be to reduce bias or increase precision. For the missingness mechanism considered here, the complete case estimate of both $\mu_{Y}$ and $\beta_{X}$ will be biased. This is illustrated graphically in Supporting Information Section 2.3 and can also be explained using causal diagrams (Moreno‐Betancur et al., 2018). In comparison, a MI procedure that includes *X*, *Z* and the auxiliary variables associated with both *Y* and $M_{Y}$ can lead to unbiased estimation of both estimands; some bias is to be expected from a MI procedure that includes *X*, *Z* and a subset of the auxiliary variables associated with both *Y* and $M_{Y}$, while variance may be reduced. Therefore, we are also interested in assessing the precision of each estimation strategy.

### 4.4 Auxiliary variable selection strategies for comparison

We compared the performance of eight data‐based strategies for selecting auxiliary variables for the imputation model for *Y* and two benchmark strategies. For each auxiliary variable selection strategy, the variable selection was done once, prior to the imputation step. The auxiliary variable selection strategies were:

1. *Quickpred‐pt2*: This approach uses the four‐step selection strategy proposed by van Buuren et al. (1999). For each i=1,⋯,p, $A_{i}$ is included in the imputation model for *Y* if the maximum of the absolute correlation between $A_{i}$ and *Y* (using all available cases) and the absolute correlation between $A_{i}$ and $M_{Y}$ is greater than 0.2. This strategy was implemented using the quickpred function from the R package mice (van Buuren & Groothuis‐Oudshoorn, 2011). The relatively low correlation of 0.2 was chosen as a reasonable cutoff for an inclusive strategy as it is halfway between the cutoffs for the *Quickpred‐pt4* and *Full* strategies (described below), and small enough to include some, but not all, of the auxiliary variables in the case study (Supporting Information Table 1).
2. *Quickpred‐pt4*: Similar to *Quickpred‐pt2*, except with the correlation cutoff set to 0.4 instead of 0.2. This cutoff was chosen based on the rule of thumb provided by Graham (2012).
3. *PredMiss*: Under this approach, auxiliary variables were selected for inclusion in the imputation model for *Y* if the *p*‐value obtained from a *t*‐test for a given auxiliary variable, carried out using $M_{Y}$ to define the two groups, was less than 0.05. In other words, this approach was used to select auxiliary variables that were predictors of missingness in *Y*.
4. *PcAux*: This approach uses principal components of auxiliary variables as predictors in the imputation model instead of the auxiliary variables themselves (Howard et al., 2015). The number of principal components was chosen such that the principal component scores explained ≥40% of the variance in the $A_{i}$s, following Howard et al. (2015). This strategy was implemented using PcAux in R (Lang et al., 2017).
5. *Forward*: Under this approach, auxiliary variables that were predictors of *Y* were chosen using forward selection (Royston & White, 2011). An auxiliary variable was added to the imputation model for *Y* at each step of the forward selection algorithm if the *p*‐value from a Wald test on the corresponding regression coefficient was less than 0.05. This strategy was implemented in Stata using the stepwise option in ice.
6. *Forward‐sw*: This approach is similar to *Forward*, except that at each step auxiliary variables could either be added to the imputation model as per above, or removed if the *p*‐value from the Wald test dropped below 0.05. It was also implemented using ice in Stata.
7. *Forward‐FMI*: This approach uses forward selection based on the FMI in the mean of *Y* (hereafter FMI) as proposed by Andridge and Thompson (2015). The forward selection procedure was initialized by estimating the FMI for each auxiliary variable in turn and selecting for inclusion in the imputation model the auxiliary variable associated with the smallest FMI. In the next step, p−1 pairs of auxiliary variables (with each pair consisting of the auxiliary variable chosen in the previous step and one of the remaining p−1 auxiliary variables) were used to create p−1 proxy variables, where the proxy variables were created as the predicted values from a linear regression of *Y* on the subset of auxiliary variables. Estimates of the FMI were obtained for each proxy variable, with the auxiliary variable from the pair resulting in the smallest estimated FMI added to the imputation model. The forward selection procedure continued in this manner until the reduction in the FMI was less than a prespecified percentage of the missing data in *Y* (1% in the scenarios where p∈{25,83,100} and 0.5% for the scenario where p=333). In Supporting Information Section 2.4, we provide a brief description of the theory and assumptions behind this strategy, and the justification for the use of this stopping rule.
8. *LASSO*: In this approach, a model for *Y* that included *X*, *Z* and all auxiliary variables was fitted using the LASSO with 10‐fold cross‐validation (Friedman et al., 2010) The regularization penalty (usually denoted by λ) was chosen to be the value that gave the most regularized model such that the cross‐validation error was within one SE of the minimum (James et al., 2021). The auxiliary variables that were included in the fitted model were included in the imputation model for *Y*, that is, the LASSO was used to select variables for the imputation model but not to estimate parameters of the imputation model. This is similar to the “indirect use of regularized regression” approach described by Zhao and Long (2016). This strategy was implemented using glmnet.

The benchmark strategies were as follows:

1. *CCA*: A complete case analysis. The subset of individuals included in the analysis was restricted to those with data for *Y*.
2. *Full*: All auxiliary variables were included in the imputation model for *Y*.

To be useful in practice, an auxiliary variable selection strategy should perform better than the *CCA* strategy by either reducing bias and/or increasing precision (see Section 7 for further comments). Ideally, the auxiliary variable selection strategy would also perform no worse than the *Full* strategy if the imputation algorithm for this strategy does not fail, and successfully produce imputations in the case that the *Full* strategy does fail.

All imputation models included *X* and *Z*, and m=30 imputations were performed, which was chosen to be equal to the percentage of missing data in *Y* (Royston & White, 2011). MI estimates were obtained using Rubin's rules (Rubin, 2004). Unless stated otherwise in the strategy descriptions above, MI was implemented using mice with linear regression in R (van Buuren & Groothuis‐Oudshoorn, 2011).

### 4.5 Performance measures

Let θ denote an unknown parameter of interest, M=2000 denote the number of simulation repetitions, ${\hat{\theta }}_{i}$ denote the estimate of θ from the *i*th simulated data set (i=1,…,M) and $se({\hat{\theta }}_{i})$ denote the SE of ${\hat{\theta }}_{i}$. We estimate θ by $\hat{\theta }=M^{-1}\sum_{i=1}^{M}{\hat{\theta }}_{i}$.

The performance of the 10 strategies (eight auxiliary variable selection strategies and two benchmark strategies) for estimating $\beta_{X}$ and $\mu_{Y}$ was compared for each of the six simulation scenarios using bias, calculated as $\hat{\theta }-\theta$, empirical SE, calculated as ${\left(M^{-1}\sum_{i=1}^{M}{\left({\hat{\theta }}_{i}-\bar{\theta }\right)}^{2}\right)}^{1/2}$, average model SE, calculated as ${\left(M^{-1}\sum_{i=1}^{M}se{\left({\hat{\theta }}_{i}\right)}^{2}\right)}^{1/2}$, and the coverage probability of the 95% confidence interval for θ, calculated as the proportion of times that θ was in the 95% confidence intervals obtained for each of the simulated data sets (Morris et al., 2019). We used the formulae provided by Morris et al. (2019) to calculate Monte Carlo SE estimates for bias and empirical SE. For bias, the Monte Carlo SE is estimated by ${\left(M^{-1}{\left(M-1\right)}^{-1}\sum_{i=1}^{M}{\left({\hat{\theta }}_{i}-\hat{\theta }\right)}^{2}\right)}^{1/2}$ and for empirical SE, by ${\left(\text{empirical SE}/(2(M-1))\right)}^{1/2}$.

We also report standardized bias, defined as (bias/empirical SE) × 100, relative bias for $\beta_{X}$, defined as (bias/true parameter value) × 100 (this cannot be calculated for $\mu_{Y}=0$), the relative error in the model SE, defined as (average model SE/empirical SE − 1) × 100, and the convergence rate, defined as the proportion of data sets for which MI estimates, were successfully obtained.

## 5 RESULTS

**FIGURE 1:**
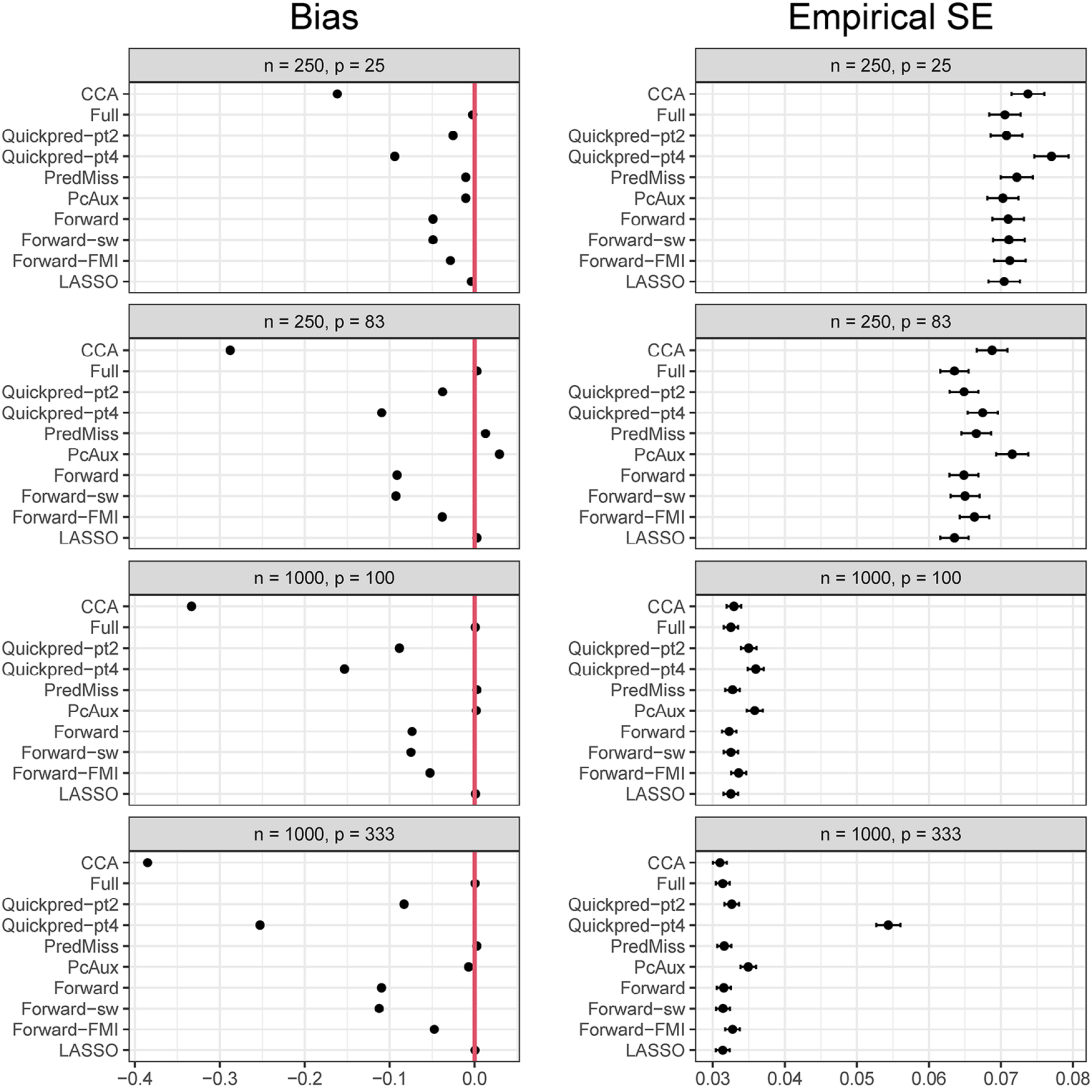
Simulation estimates of bias and empirical standard error (SE) for $\mu_{Y}$ (true value = 0) from each analysis strategy and the four scenarios where *Y* had a 30% chance of being missing and the coefficients in the missingness model were log (1.2). Estimates are presented with 95% Monte Carlo confidence intervals to quantify simulation uncertainty.

**FIGURE 2:**
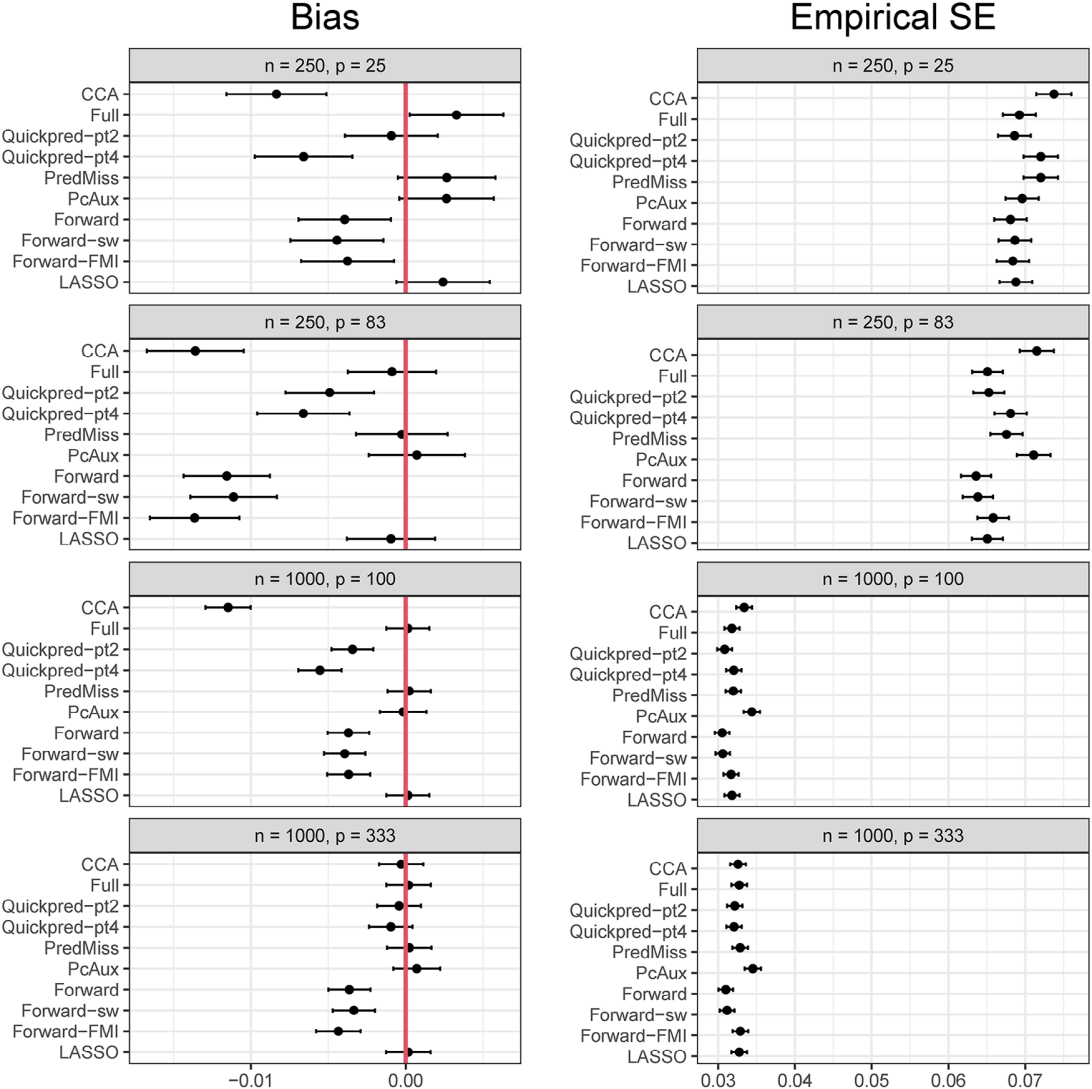
Simulation estimates of bias and empirical standard error (SE) for $\beta_{X}$ (true value = 0.18 for the scenarios where p=25 and p=83, and 0.09 for the scenarios where p=100 and p=333) from each analysis strategy and for the four scenarios where *Y* had a 30% chance of being missing and the coefficients in the missingness model were log (1.2). Estimates are presented with 95% Monte Carlo confidence intervals to quantify simulation uncertainty.

Figures 1 and 2 present simulation estimates of bias and empirical SE for $\mu_{Y}$ and $\beta_{X}$, respectively, for each analysis strategy and for the four scenarios where the proportion of missing data in *Y* was 30% and the coefficients in the missingness model were log (1.2). Results for all six scenarios are summarized below and the full results are presented in Supporting Information Tables 2–4. There were no problems with convergence of any of the analysis approaches, with estimates produced on all simulation runs.

### 5.1 Mean of *Y*

As expected, estimating $\mu_{Y}$ using complete cases led to the largest bias, while using MI with all auxiliary variables in the imputation model was approximately unbiased across all scenarios. Within each scenario, all auxiliary variable selection strategies led to smaller absolute bias than the complete case analysis. The smallest standardized bias was observed for the *Full* and *LASSO* strategies, with *Full*, *LASSO*, and *PredMiss* producing standardized bias of less than 30% in each scenario, an amount that is considered not to be problematic by Collins et al. (2001). The *Full* and *LASSO* strategies led to lower empirical SEs than the complete case analysis in five of the six scenarios. The remaining MI strategies had larger empirical SEs compared to the complete case analysis in at least two of the six scenarios, with *Quickpred‐pt4* and *PcAux* producing larger empirical SEs than the complete case analysis in five of the six scenarios. The absolute relative errors in the model SE were largest for the *Quickpred‐pt4* strategy in the n=1000, p=333 scenario (41%), and the scenario with the stronger missingness mechanism (−14% for *Quickpred‐pt2*, −15% for the three forward selection strategies, and −25% for *Quickpred‐pt4* in the scenario with the stronger missingness mechanism). For all other scenarios and strategies the absolute relative error in the model SE did not exceed 9%. Estimating $\mu_{Y}$ using complete cases led to the lowest coverage probability in each scenario (41% in the n=250,p=25 scenario, and ⩽1% in all other scenarios). *Quickpred‐pt2*, *Quickpred‐pt4*, *Forward*, *Foward‐sw*, and *Forward‐FMI* all led to lower than nominal coverage probability (ranging from 0% to 93% across methods and scenarios, with the lowest values obtained for the scenario with the strong missingness mechanism), while the *Full*, *PredMiss*, *PcAux*, and *LASSO* strategies had reasonable coverage probability across all scenarios (between 94% and 96%).

### 5.2 Regression coefficient of *X*

The complete case analysis led to absolute standardized bias of more than 30% in three of the six scenarios: the three scenarios where n=1000 and p=100. All MI analysis strategies led to absolute standardized bias of less than 30% across all scenarios, with *Full*, *PcAux*, *PredMiss*, and *LASSO* producing absolute standardized bias values of less than 5% across all scenarios. All MI strategies produced smaller empirical SEs than the complete case analysis for the two scenarios with n=250 and the scenario with n=1000, p=100 and 50% missing values. For the remaining scenarios, *PcAux* resulted in the highest empirical SEs. The model SE was a reasonable estimate of the empirical SE for all strategies and scenarios, with the largest absolute relative error in the model SE of 3.3% obtained from the *Quickpred‐pt2* strategy for the scenario with n=1000,p=100, 30% missing values and coefficients of the missingness model equal to log (1.2). The complete case analysis led to slightly lower than nominal coverage probabilities of 92% in the scenario with 50% missing values and 93% in the scenario with the stronger missingness mechanism. The remaining complete case analysis estimates of coverage probability were between 94% and 96%. All MI analysis strategies produced estimates of coverage probability between 94% and 96%.

### 5.3 Selected auxiliary variables

**FIGURE 3:**
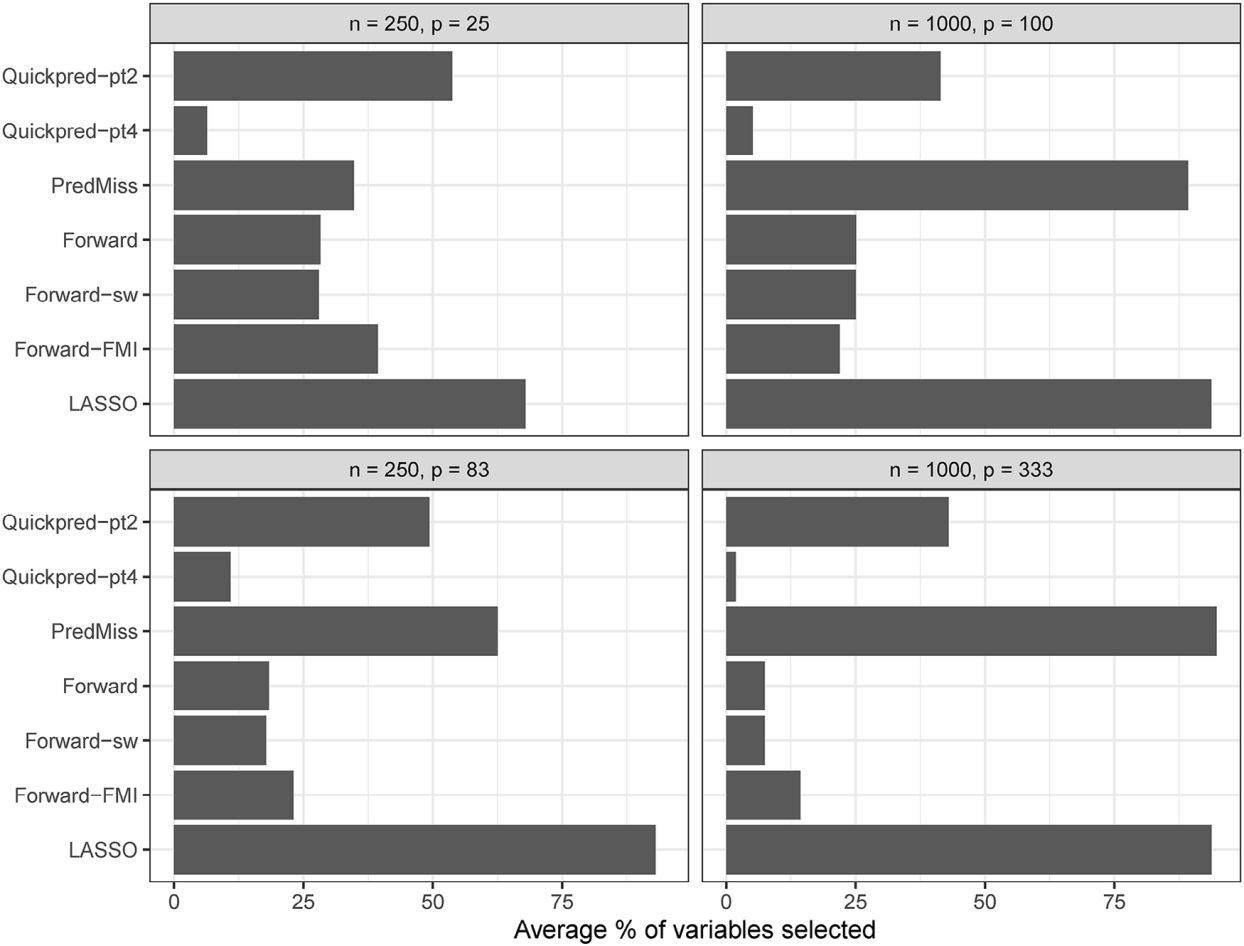
Average number of auxiliary variables selected across simulation runs, expressed as a proportion of the total number of auxiliary variables, for relevant analysis strategies and the four scenarios where *Y* had a 30% chance of being missing and the coefficients in the missingness model were log (1.2).

Figure 3 illustrates the average number of auxiliary variables selected across simulation runs, expressed as a proportion of the total number of auxiliary variables, for the relevant analysis strategies and for the four scenarios where *Y* had a 30% chance of being missing and the coefficients in the missingness model were log (1.2). This figure does not include *PcAux* since this strategy included auxiliary variable information via principal component scores. In Supporting Information Section 2.6, we explore the selected auxiliary variables further by providing similar figures, stratified by auxiliary variable group, where the groups are determined by (i) the true correlations between *Y* and the auxiliary variables and (ii) the relationship between $M_{Y}$ and the auxiliary variables. The *LASSO* strategy was the most inclusive strategy overall (determined by the total average number of auxiliary variables included) and the *Quickpred‐pt4* strategy was the least inclusive strategy overall. As expected, auxiliary variables that were highly correlated with *Y* were selected more often than those with low correlation. In general, the *PredMiss*, *Quickpred‐pt2* and *Quickpred‐pt4* strategies selected a higher number of auxiliary variables that appeared in the missingness model relative to those that did not, while the opposite was found for the *Forward*, *Forward‐sw* and *Forward‐FMI* strategies (although this pattern differed slightly across simulation scenarios).

## 6 APPLICATION OF STRATEGIES TO THE LSAC EXAMPLE

To illustrate the implication of the method choice in practice, we applied each of the auxiliary variable selection strategies considered in the simulation study to the LSAC case study described earlier. We also carried out the benchmark strategies *CCA* (in R) and *Full* (in both R and Stata). Continuous variables (including SDQ and GHM) were imputed using linear regression, except for HRQoL, which was imputed using predictive mean matching (PMM, a semiparametric approach where values are imputed using random draws from the observed data of a set of donors with similar predicted means, Morris et al., 2014) with five donors because this variable exhibited negative skew. Binary variables were imputed using logistic regression and PedsQL items were imputed using ordinal logistic regression. The augment option was used in Stata to perform augmented regression in the presence of perfect prediction for all categorical imputation variables. All imputation models included all analysis model variables (to ensure congeniality), with auxiliary variables chosen according to the analysis strategy. A total of 20 imputations were performed, with 10 iterations for each imputation.

### 6.1 Implementation of auxiliary variable selection strategies

To accommodate the additional complexities of multivariate missingness and different types of variables, the auxiliary variable selection strategies were implemented as follows. For the *Quickpred‐pt2* and *Quickpred‐pt4* strategies, the quickpred function was used to select the auxiliary variables in the imputation model for each of the incomplete variables using the correlation cutoffs of 0.2 and 0.4, respectively. Correlations between two variables were calculated using all complete pairs of observations on those variable. For the *PredMiss* strategy, *t*‐tests (for continuous variables) or chi‐squared tests (for categorical variables) were used to select the auxiliary variables in the imputation model for each of the incomplete variables. The two groups to be compared were determined by the missingness indicator of the incomplete variable and consisted of the observed values of the auxiliary variables. An auxiliary variable was selected for inclusion in the imputation model for the incomplete variable if the *p*‐value obtained from the hypothesis test was less than 0.05 and a sufficient number of observations were available in each group (≥20 for the *t*‐test and all expected counts ≥5 for the chi‐squared test). The *PcAux* strategy required imputation of the missing values in the auxiliary variables prior to obtaining principal components. This was done using the PcAux package (Lang et al., 2017), which implements a single imputation using PMM within the MICE approach. The first eight principal components of the auxiliary variables, which explained approximately 44% of the variance in these variables, were included in the imputation models for the remaining five incomplete variables in the analysis model.

For the *Forward* and *Forward‐sw* strategies, the stepwise algorithm was used to select the auxiliary variables in the imputation model for each of the incomplete variables, after an initial imputation to obtain a complete data set. Dummy variables for the ordinal PedsQL items were grouped during the variable selection step to ensure they were either all included in, or excluded from, the imputation model. The *Forward‐FMI* method does not currently handle missing values in auxiliary variables or highly negatively skewed ordinal variables (Andridge & Thompson, 2015). Rather than extend this methodology (which is beyond the scope of this study), we deemed this method not suitable to use in this case study. For the *LASSO* strategy, we wanted to implement the grouped LASSO to ensure dummy variables for PedsQL items were either all included or excluded from the imputation model (c.f. *Forward* and *Forward‐sw*). However, it is not clear how to apply the grouped LASSO with ordinal outcomes. Furthermore, the LASSO requires predictors (here the analysis model variables and the candidate auxiliary variables) to be complete. Rather than imputing the auxiliary variables, we proceeded as follows. A model for *HRQoL* that included all variables in the analysis model and all candidate auxiliary variables was fitted to the complete cases using the grouped LASSO with 10‐fold cross‐validation (implemented using the gglasso package in R). The regularization penalty was chosen to be the value that gave the most regularized model such that the cross‐validation error was within one SE of the minimum. Each auxiliary variable selected using the LASSO was included in the imputation model for each of the incomplete variables in the analysis model, as well as the imputation model for all other incomplete auxiliary variables selected by the LASSO. Auxiliary variables not selected by the LASSO were excluded from the imputation process altogether.

### 6.2 Results

Estimates from *Full*, *Forward* and *Forward‐sw*, which were implemented in Stata, were not obtained because the imputation procedure failed due to lack of convergence and no imputations were produced. For the *Full* strategy, this failure occurred when fitting an ordinal logistic regression model during the imputation procedure. For the *Forward* and *Forward‐sw* strategies, these failures occurred during the auxiliary variable selection step for one of the PedsQL items. Although the R strategies produced estimates, problems were reported via the list of logged events reported by mice in the *Full* and *PredMiss* strategies (all of which involved PedsQL items and were handled in the algorithm by removal of a contrast). No problems were reported for the other MI strategies. Table 1 presents the auxiliary variables selected for inclusion in the imputation model for HRQoL for each of the MI strategies that produced estimates and the computational time taken by each strategy on a high‐performance computing cluster node with an Intel(R) Xeon(R) Gold 6126 CPU @ 2.60 GHz and 16 GB of RAM. *Quickpred‐pt2* and *PredMiss* were the most inclusive auxiliary variable selection strategies, followed by the *LASSO*. Employing auxiliary variable selection led to substantially decreased computational time relative to the *Full* strategy.

**TABLE 1:** Auxiliary variables selected for inclusion in the imputation model for HRQoL and computational time taken for auxiliary variable selection (Selection), to produce MI estimates (Estimation) and in total.

	Selected variables		Computational time (min)		
Strategy	Details	Number	Selection	Estimation	Total
*Full* ^a^	All	85	–	3003.3	3003.3
*Quickpred‐pt2*	54 PedsQL items (8 at w1, 23 at w2, 23 at w3); GHM (w2–w4); SDQ (w1–w4)	61	0.02	666.2	666.2
*Quickpred‐pt4*	SDQ (w2–w4)	3	0.02	86.8	86.8
*PredMiss*	38 PedsQL items (19 at w1, 8 at w2, 11 at w3); GHM (w1–w3); SDQ (w1–w3); MR (w2–w4); PPVT (w1–w3)	50	0.8	946.1	946.9
*PcAux*	First 8 principal components of auxiliary variables	8	8.7	0.6	9.3
*LASSO*	7 PedsQL items (3 at w2, 4 at w3); GHM (w4); SDQ (w1–w4); MR (w2 and w3); PPVT (w1–w3)	17	9.9	23.2	33.1

*Note*: The complete case analysis took approximately one‐tenth of a second of computational time.

Abbreviations: GHM, Global Health Measure; MR, Matrix Reasoning Test; PedsQL, Pediatric Quality of Life Inventory; PPVT, Peabody Picture Vocabulary Test; SDQ, Strengths and Difficulties Questionnaire; w, wave.

^a^ Results are from implementing the *Full* strategy in R as this strategy failed in Stata.

**FIGURE 4:**
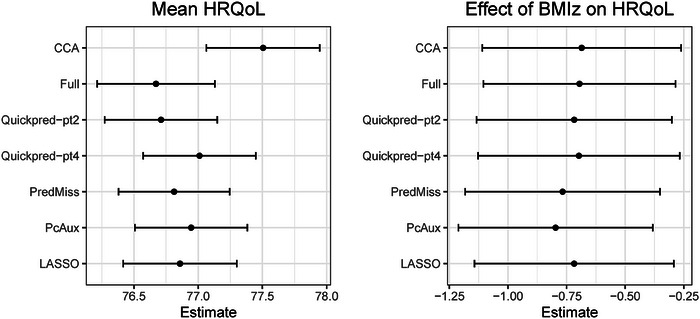
Estimates of the mean HRQoL (left panel) and the effect of BMI*z* on HRQoL (right panel), with 95% confidence intervals, from seven analysis strategies.

Figure 4 presents estimates, with 95% confidence intervals, for the effect of BMI*z* on HRQoL (β_1_ in model (1)) and the mean HRQoL for the LSAC case study, for the analysis strategies that produced estimates. All analysis strategies led to similar estimates of $\beta_{X}$ (ranging from −0.80 to −0.69) and therefore similar conclusions. That is, a one‐unit increase in BMI *z*‐score in Australian children aged 4 to 5 is associated with a decrease in HRQoL at age 10 to 11 of around 0.7 units. Estimates of the mean HRQoL ranged from 76.7 to 77.5, with estimates produced by all MI strategies noticeably lower than estimates from the complete case analysis.

## 7 DISCUSSION

Although data‐driven auxiliary variable selection is commonly used in the application of MI, there has been limited investigation of proposed strategies. We used a simulation study to compare the performance of eight data‐based strategies for selecting auxiliary variables for inclusion in the imputation procedure when MI was used to estimate the mean outcome and an exposure–outcome association, for an incomplete outcome. We also compared the approaches with an analysis of complete cases and an MI analysis with all auxiliary variables in the imputation model. MI with the full imputation model performed well across all four scenarios considered, regardless of whether the target of analysis was the exposure–outcome association or the mean outcome. All auxiliary variable selection strategies generally led to smaller absolute bias than the complete case analysis across scenarios and for both estimands. The *LASSO* was the best performing and most inclusive auxiliary variable selection strategy overall (based on bias, empirical SE and coverage probability across all simulation scenarios). This strategy had the advantage of incorporating all auxiliary variable information when selecting variables for the imputation model (rather than information from multiple pairwise comparisons). However, it was not clear how to best extend this approach to the case study where there were the additional complexities of missing data in auxiliary variables and highly skewed ordinal variables. The *PcAux* and *PredMiss* strategies also had minimal bias across all simulation scenarios, but had higher SE relative to the *LASSO*. The *Forward‐FMI* strategy was less biased than the other two forward selection strategies for estimating the mean of the incomplete outcome, but all three strategies led to similar bias when estimating the exposure–outcome association. This may be explained by the fact that the *Forward‐FMI* strategy selects variables based on minimizing the FMI in the mean of the incomplete outcome.

This study adds to the limited body of research on auxiliary variable selection for MI. Early work in this area illustrated that auxiliary variables can improve the performance of MI and led to the recommendation of an inclusive strategy for variable selection (Collins et al., 2001). However, the study by Collins et al. (2001) considered just two auxiliary variables that were correlated with either the incomplete variable or the missingness of the incomplete variable. In large‐scale longitudinal studies there are often many potential auxiliary variables to choose from. The results of the current study provide support for the inclusive auxiliary variable strategy when there are a larger number of auxiliary variables and a mix of realistic relationships between these variables and an incomplete outcome. This is illustrated, for example, by the *Full* strategy performing better than the *Quickpred‐pt2* strategy, which in turn performs better than the *Quickpred‐pt4* strategy in terms of bias across scenarios.

When applied to the LSAC case study, all MI strategies led to similar estimates, but strategies using a subset of the auxiliary variables were substantially faster in terms of computational time. For example, *Quickpred‐pt2* included 61 (72%) of the 85 auxiliary variables but required approximately one‐fifth of the computational time of the *Full* strategy, while *LASSO* included 17 (20%) of the 85 auxiliary variables and was over 90 times faster than *Full*. The quickpred strategies are attractive in practice due to their simplicity, the ease at which they extend to more complicated problems (such as the LSAC case study) and their implementation in statistical software. Based on the results of our simulation study, we would recommend the use of a low correlation cutoff when using quickpred. In contrast, there is currently no software freely available to apply the *Forward‐FMI* strategy. Combined with the availability of other auxiliary variable selection strategies that performed better in our simulation study and the difficulties that arise when trying to extend this strategy to more complex scenarios, the practical utility of the *Forward‐FMI* strategy is questionable.

Although appealing, employing an inclusive strategy may be problematic when there are many auxiliary variables available. We encountered convergence problems when analyzing the case study with Stata using the *Full*, *Forward*, and *Forward‐sw* strategies. Results were obtained when the *Full* strategy was implemented in R, but with a number of logged events reported on each iteration of the imputation algorithm. Others have also noted convergence problems when using MICE in Stata (Nguyen et al., 2021; Simons et al., 2015), and one simulation study using MICE in both Stata and R found that, although R did not encounter the same convergence problems as Stata, the estimates obtained in R were less accurate than estimates from a MVNI‐based approach (De Silva et al., 2021). A comprehensive comparison of the MI algorithms in these two popular statistical software packages is an area for future research. The lack of convergence problems in our simulation study is likely due to the simulated data sets containing only continuous variables (and only one incomplete variable), with no very high correlations between these variables. Instead of reducing or modifying the set of auxiliary variables, one could try several alternative approaches to produce imputations when the MI algorithm fails. These include changing the imputation model form, for example, instead of ordinal logistic regression to impute ordinal variables, one could try linear regression or PMM; changing the imputation method, for example, for missing data in more than one variable, MVNI could be used instead of MI by chained equations (Lee & Carlin, 2010); or changing the level at which variables are imputed (applicable for derived variables or scale scores, Mainzer et al., 2021; Nguyen et al., 2021). Alternatively, one may attempt to identify and then remove from the imputation model the variables that appear to be responsible for the convergence problems. However, these approaches also have limitations. We did not consider such alternative approaches in this study.

There are a number of challenges in evaluating auxiliary variable selection strategies due to the large amount of data that needs to be generated. One challenge in evaluating auxiliary variable selection strategies is generating realistic data, with a mix of relationships between variables. One study by Hardt et al. (2012) considered two values of the correlations between all auxiliary variables and other variables in the study: low (0.1) and moderate (0.5). Another study by Howard et al. (2015) took a similar approach, varying correlation values from 0.1 to 0.6. In practice, it is highly unlikely for all auxiliary variables to have the same relationship with the variable with missing values. Our simulation study was designed to assess the performance of strategies for selecting auxiliary variables when there was a mix of relationships (i) between auxiliary variables and the incomplete variable, and (ii) between auxiliary variables and the missingness indicator of the incomplete variable. In contrast to our study, both Collins et al. (2001) and Hardt et al. (2012) showed that including many auxiliary variables in the imputation model can lead to bias in MI estimates. We did not observe bias from our *Full* analysis strategy in any scenario considered. However, our study was not set up to address limits on the number of auxiliary variables that should be included. We also acknowledge that some bias is expected from estimation procedures that involve auxiliary variable selection in settings such as ours because, by design, they do not condition on the full set of auxiliary variables associated with both *Y* and $M_{Y}$. In this paper, we considered an auxiliary variable strategy to perform better than the complete case analysis if it reduced bias and/or increased precision compared to the complete case analysis. However, we note that there are cases in which MI will (appropriately) result in larger SEs than a complete case analysis as the uncertainty in the missing values is taken into account.

There are many variations on the ways in which the auxiliary variable selection strategies may be implemented that were not considered in this paper. For the quickpred strategies, one could consider adjusting the correlation cutoff such that a specified number of auxiliary variables with the highest correlations are chosen or exclude auxiliary variables with too many missing values (van Buuren, 2018; van Buuren et al., 1999). For the *PcAux* strategy, the number of principal components to include in the imputation model was chosen such that the principal component scores explained ≥40% of the variance in the auxiliary variables. One may consider a different proportion of variance explained or employ other methods for specifying the number of principal components to be used. The pcaux function provides an option (nComps = Inf) to use the smallest number of component scores such that adding one more component score does not make a discernable difference in the amount of variance explained. Also, the *p*‐value threshold may be changed in the *Forward*, *Forward‐sw*, and *PredMiss* strategies to include different subsets of auxiliary variables. We used the LASSO for variable selection only, since the aim of the study was to compare methods used to select auxiliary variables for MI. We note that it would be impossible to apply the *LASSO* strategy as it was implemented in the case study if there are few to no cases for which all analysis and auxiliary variables are completely observed. We also note that there are alternative ways to incorporate the LASSO with MI that may perform better (Zhao & Long, 2016). Finally, it would be interesting to examine auxiliary variable selection strategies in other contexts where auxiliary variables are utilized, for example, survival analysis (Hsu et al., 2006).

This study considered a natural starting point for evaluating a range of auxiliary variable selection strategies for MI. However, several limitations of this study should be noted. We considered just one incompletely observed variable in our simulation study. The extent to which the conclusions from our simulation study extend to the case where there are multiple incomplete variables is not clear. Further work is needed to explore the performance of auxiliary variable selection strategies in the presence of multivariable missingness. All variables in our simulation study were normally distributed and the strength of the missing data mechanism was the same for each variable associated with missingness. These simplifying aspects are unlikely to hold in practice. However, we have purposefully chosen to start with this simpler scenario to avoid the results being obscured by complexities such as mixed variable types. When there are a large number of categorical auxiliary variables (such as in the case study), perfect prediction can cause problems with MI. Others have considered how to do MI in this scenario (Mainzer et al., 2021; Nguyen et al., 2021). We assumed the existence of a candidate set of auxiliary variables. A reviewer observed that determining this set is also challenging. We agree and see this as a topic for future research. We also acknowledge that, although not the case in the current study, including auxiliary variables in the imputation procedure may introduce bias into the MI estimates (Thoemmes & Rose, 2014). Although certain auxiliary variables were excluded from the missingness model by design, exploration revealed there were often small marginal correlations induced between these auxiliary variables and the missingness of the incomplete outcome. (This fact, combined with the moderate to large sample sizes, may explain the inclusiveness of the *PredMiss* strategy.) Finally, we chose to focus on six scenarios. By doing so, we may have overlooked a range of scenarios under which the analysis strategies perform poorly. We note that data‐based variable selection strategies are known to have limitations in other contexts (Greenland, 1989; Kabaila, 2009). However, this study can be used to inform further research in this area.

In conclusion, this study evaluated a range of strategies for selecting auxiliary variables for MI models. MI using all auxiliary variables in the imputation model performed well in all scenarios considered, providing further support for adopting an inclusive auxiliary variable strategy where possible. Auxiliary variable selection using the LASSO was the best performing auxiliary variable selection strategy overall and may be a promising alternative when the full model fails or is too burdensome. Quick data‐based selection of auxiliary variable, as implemented in the mice R package, performed reasonably in the simulation study when used with a low correlation cutoff and was straightforward to extend to the complexities of a real case study.

## CONFLICT OF INTERESTS STATEMENT

The authors declare no conflict of interest.

### OPEN RESEARCH BADGES

This article has earned an Open Data badge for making publicly available the digitally‐shareable data necessary to reproduce the reported results. The data is available in the Supporting Information section.

This article has earned an open data badge “**Reproducible Research**” for making publicly available the code necessary to reproduce the reported results. The results reported in this article were reproduced partially due to undisclosed data.

## Supporting information

Supporting Information

Supporting Information

## Data Availability

The data that support the findings of this study are available from the DSS. Restrictions apply to the availability of these data, which were used under license for this study. Data are available at https://dataverse.ada.edu.au with the permission of the DSS. All code used to produce the results in this manuscript is available on GitHub: https://github.com/rheanna‐mainzer/MI‐aux‐var‐selection.
