# Supplementary material for: A comparison of strategies for selecting auxiliary variables for multiple imputation[image]
Source: Biom J. Author manuscript; Available in PMC 2024 Mar 8. (PMC7615727; doi:10.1002/bimj.202200291)
Supplement: Supporting Information 1 [file EMS194352-supplement-Supporting_Information_1.zip › code_resubmitted/sim_study/results/figures/suppfig5.pdf]

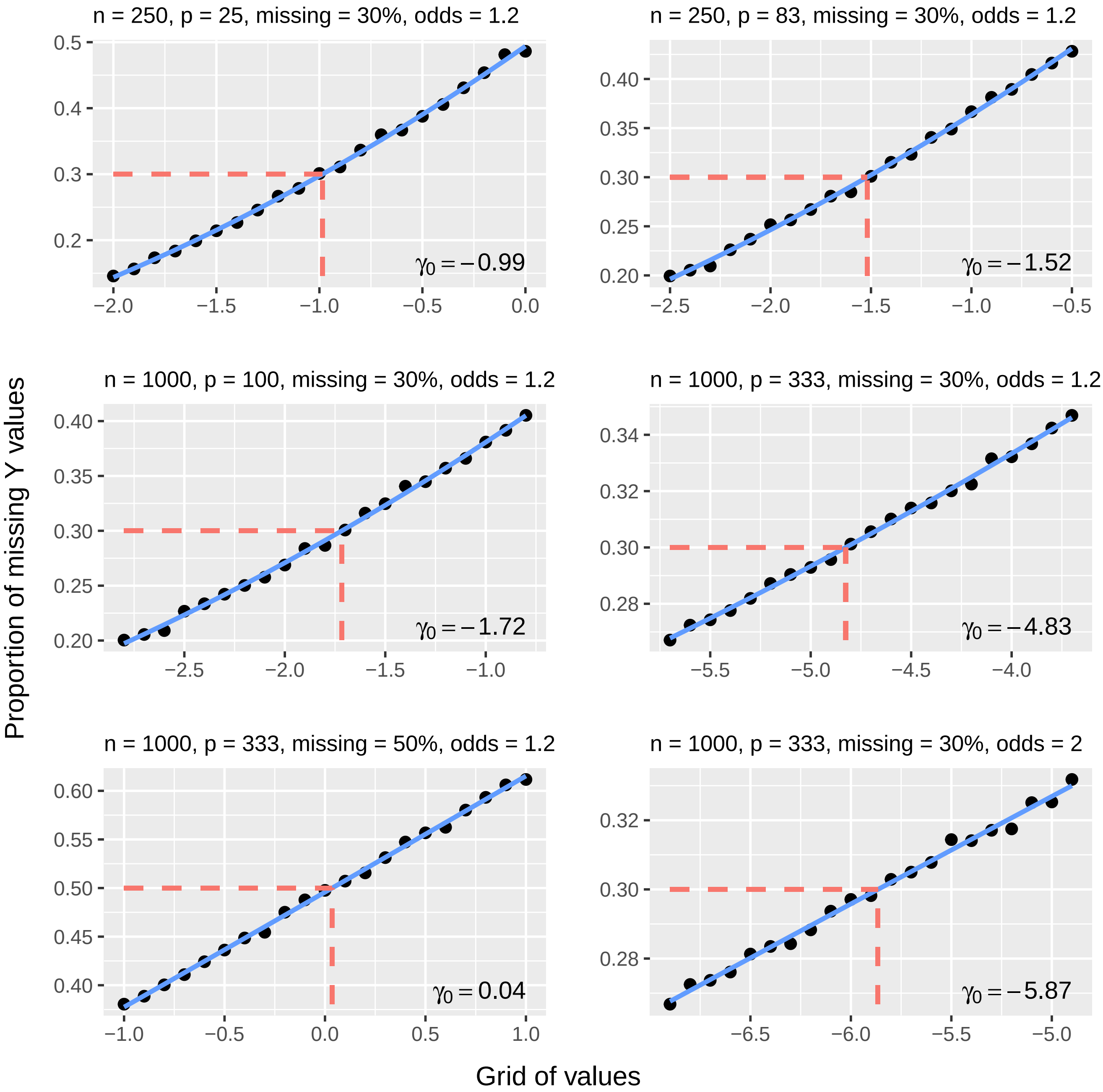
n = 250, p = 25, missing = 30%, odds = 1.2
γ0 = −0.99
n = 250, p = 83, missing = 30%, odds = 1.2
γ0 = −1.52
n = 1000, p = 100, missing = 30%, odds = 1.2
γ0 = −1.72
n = 1000, p = 333, missing = 30%, odds = 1.2
γ0 = −4.83
n = 1000, p = 333, missing = 50%, odds = 1.2
γ0 = 0.04
n = 1000, p = 333, missing = 30%, odds = 2
γ0 = −5.87
Proportion of missing Y values
Grid of values
