# Supplementary material for: A comparison of strategies for selecting auxiliary variables for multiple imputation[image]
Source: Biom J. Author manuscript; Available in PMC 2024 Mar 8. (PMC7615727; doi:10.1002/bimj.202200291)
Supplement: Supporting Information 1 [file EMS194352-supplement-Supporting_Information_1.zip › code_resubmitted/sim_study_MWE/results/figures/suppfig5.pdf]

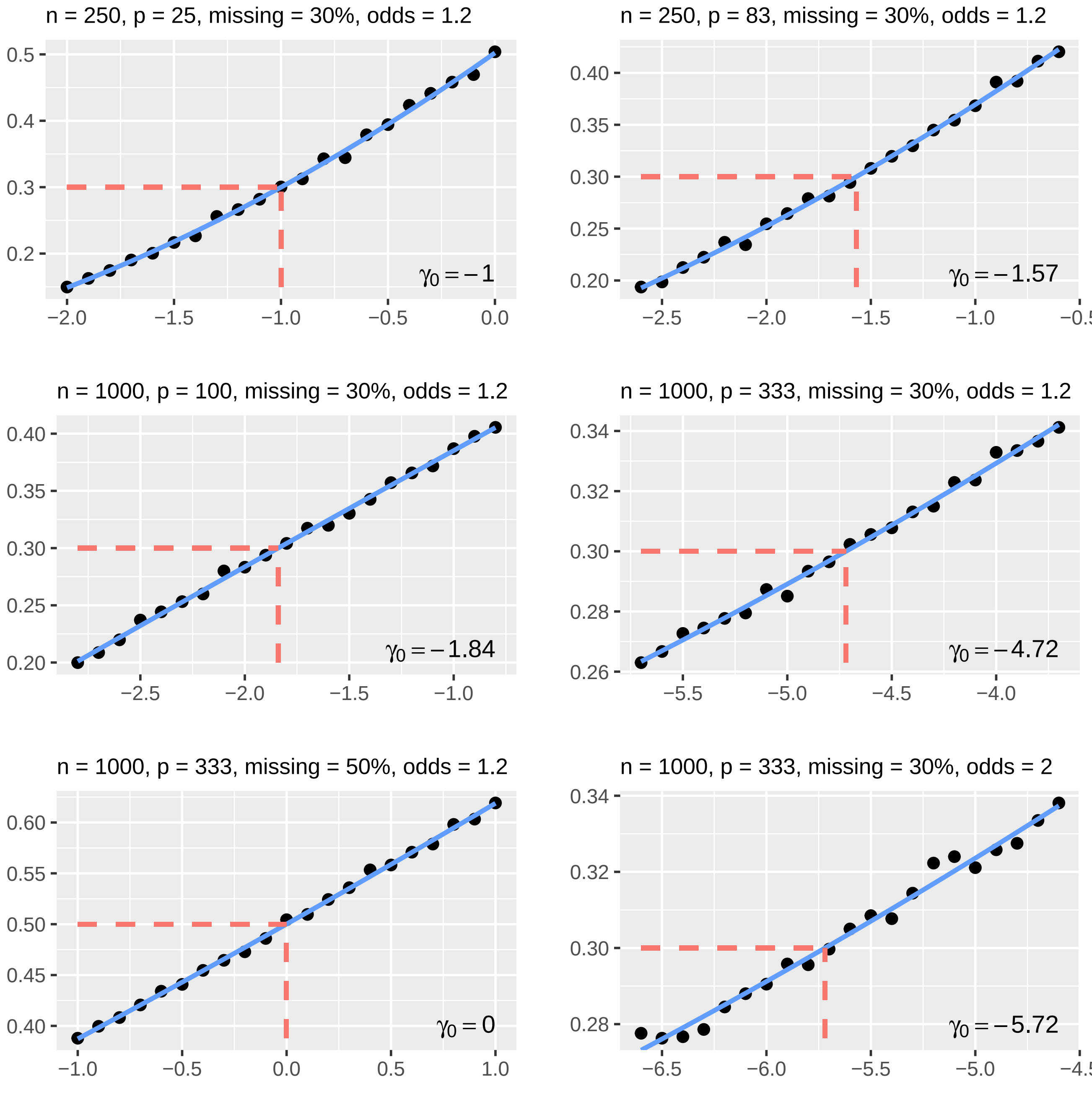

n = 250, p = 25, missing = 30%, odds = 1.2
0.5
0.4
0.3
0.2
γ0 = −1
−2.0
−1.5
−1.0
−0.5
0.0
n = 250, p = 83, missing = 30%, odds = 1.2
0.40
0.35
0.30
0.25
0.20
γ0 = −1.57
−2.5
−2.0
−1.5
−1.0
n = 1000, p = 100, missing = 30%, odds = 1.2
0.40
0.35
0.30
0.25
0.20
γ0 = −1.84
−2.5
−2.0
−1.5
−1.0
n = 1000, p = 333, missing = 30%, odds = 1.2
0.34
0.32
0.30
0.28
0.26
γ0 = −4.72
−5.5
−5.0
−4.5
−4.0
n = 1000, p = 333, missing = 50%, odds = 1.2
0.60
0.55
0.50
0.45
0.40
γ0 = 0
−1.0
−0.5
0.0
0.5
1.0
n = 1000, p = 333, missing = 30%, odds = 2
0.34
0.32
0.30
0.28
γ0 = −5.72
−6.5
−6.0
−5.5
−5.0
