# Supplementary figures and images for: A comparison of strategies for selecting auxiliary variables for multiple imputation[image]
Source: Biom J. Author manuscript; Available in PMC 2024 Mar 8. (PMC7615727; doi:10.1002/bimj.202200291)

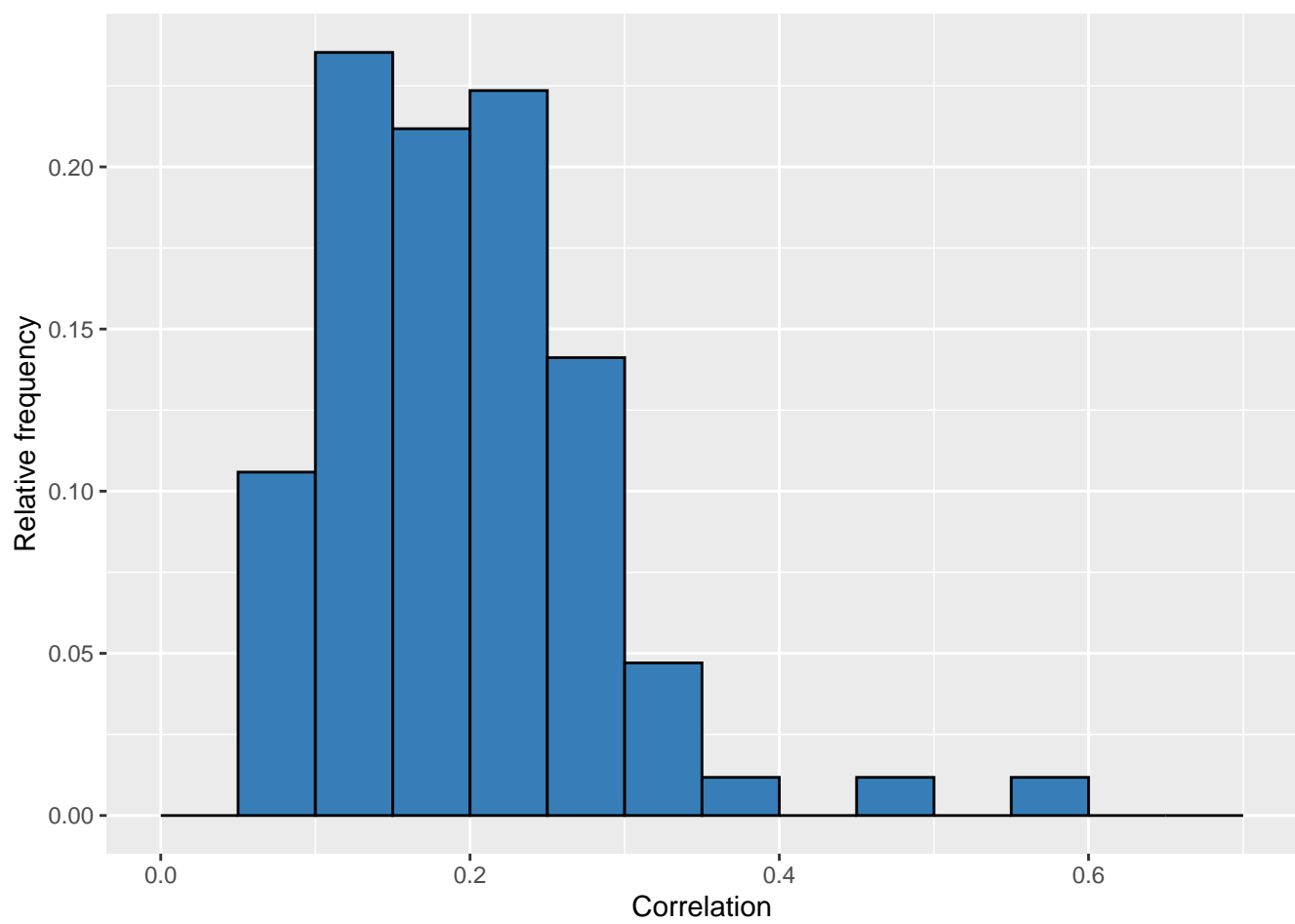

Supplement: Supporting Information 1 [file EMS194352-supplement-Supporting_Information_1.zip › code_resubmitted/case_study_synth/results/SuppFig_corrs_synth.pdf]

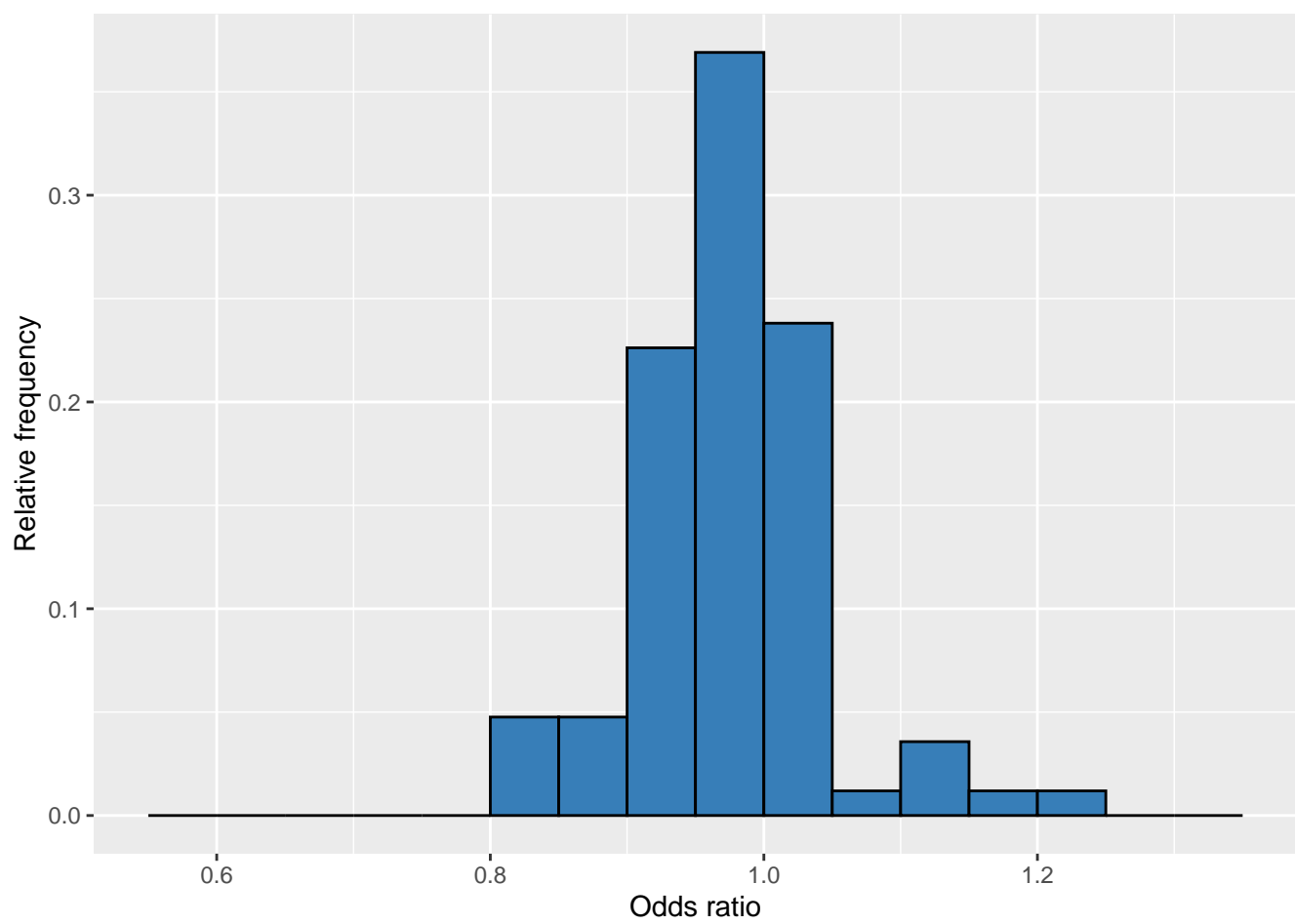

Supplement: Supporting Information 1 [file EMS194352-supplement-Supporting_Information_1.zip › code_resubmitted/case_study_synth/results/SuppFig_ORs_synth.pdf]

# Bias

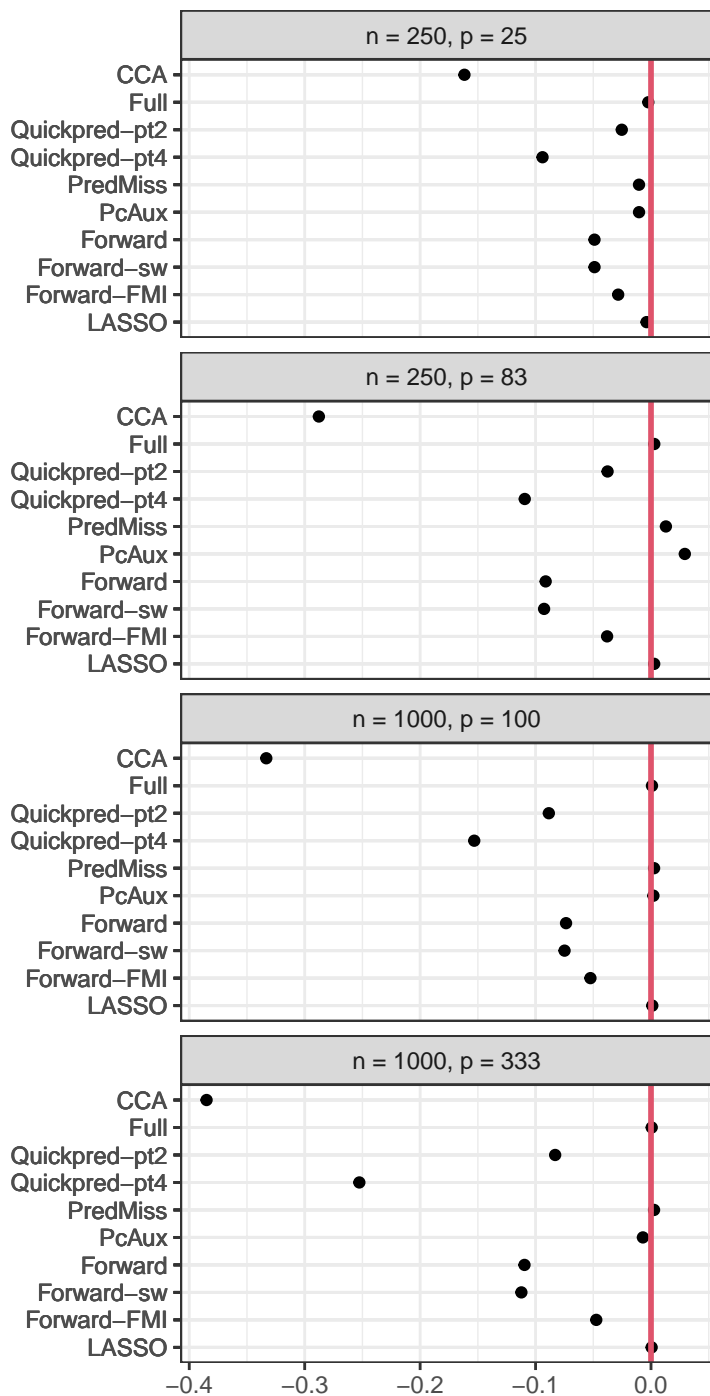

# Empirical SE

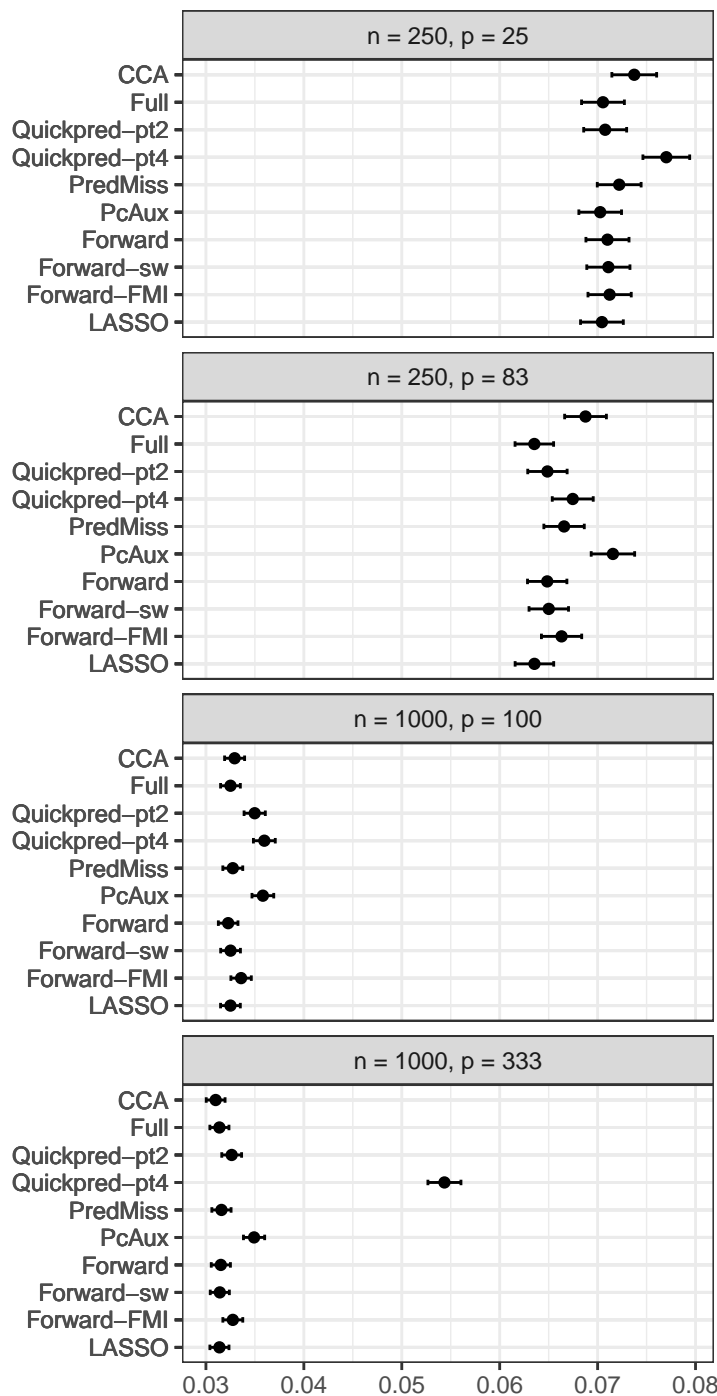

Supplement: Supporting Information 1 [file EMS194352-supplement-Supporting_Information_1.zip › code_resubmitted/sim_study/results/figures/Fig1_meany.pdf]

# Bias

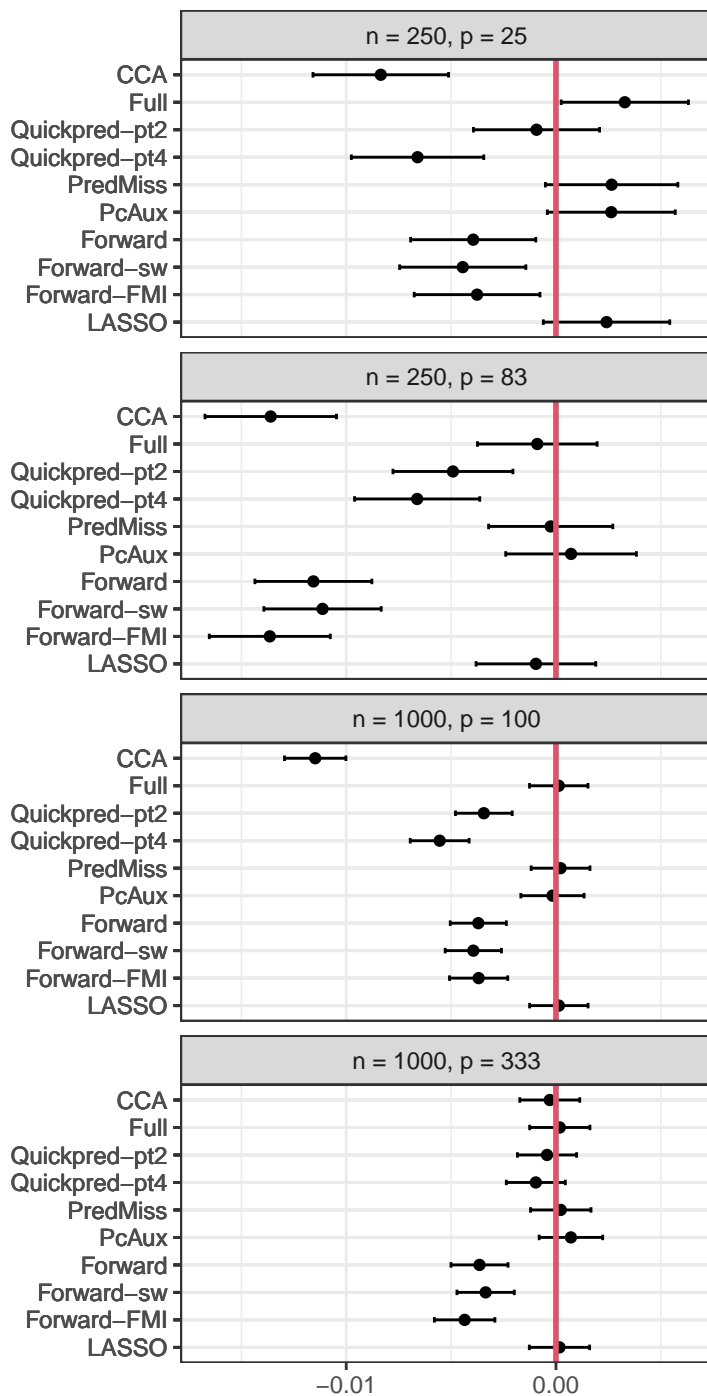

# Empirical SE

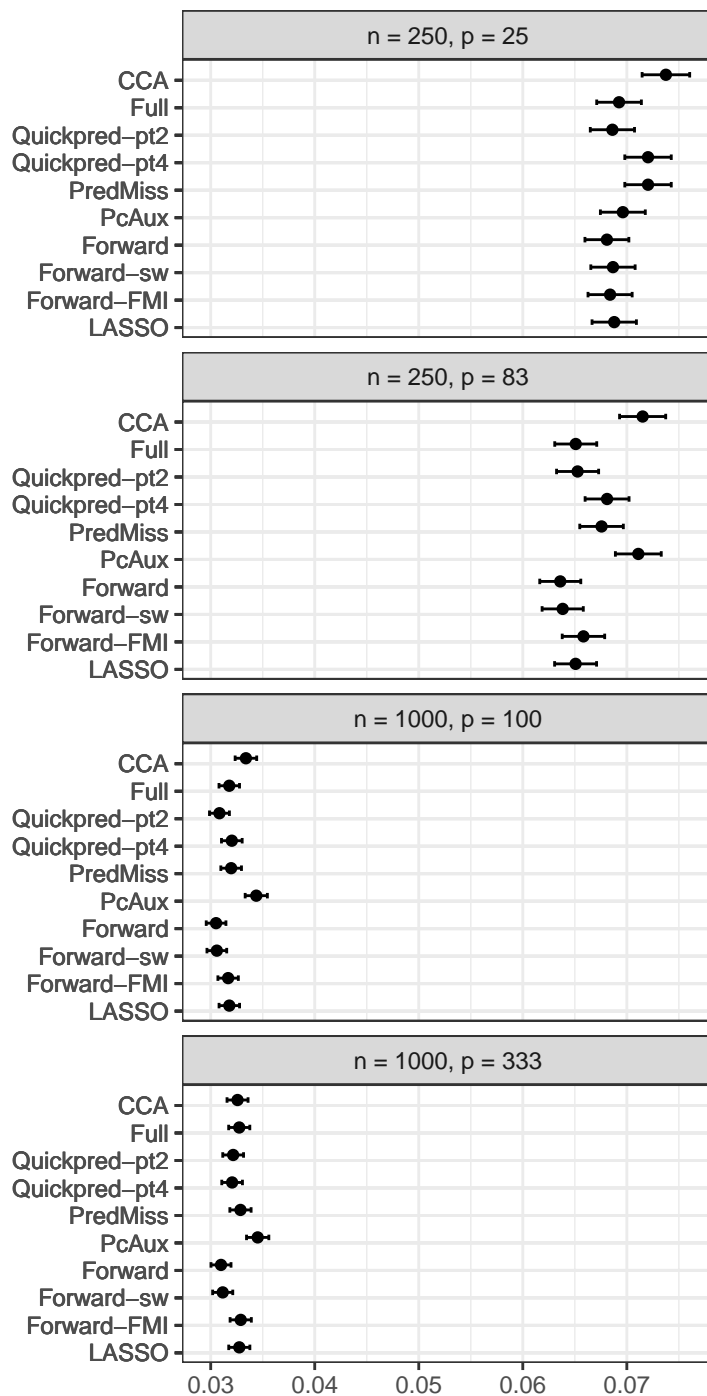

Supplement: Supporting Information 1 [file EMS194352-supplement-Supporting_Information_1.zip › code_resubmitted/sim_study/results/figures/Fig2_betax.pdf]

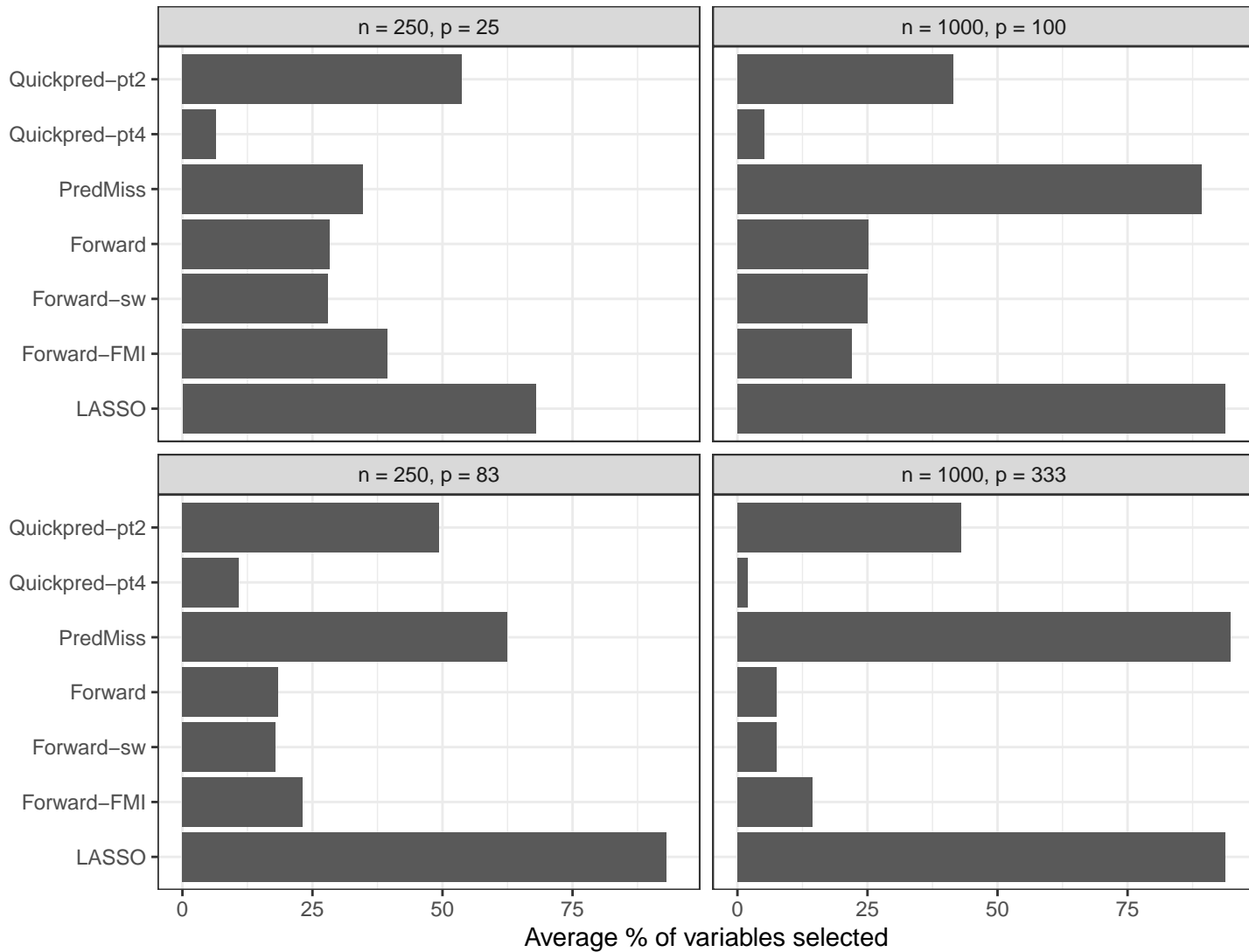

Supplement: Supporting Information 1 [file EMS194352-supplement-Supporting_Information_1.zip › code_resubmitted/sim_study/results/figures/Fig3_TotalAuxVars.pdf]

$n = 1000$ ,  $p = 100$

**A**

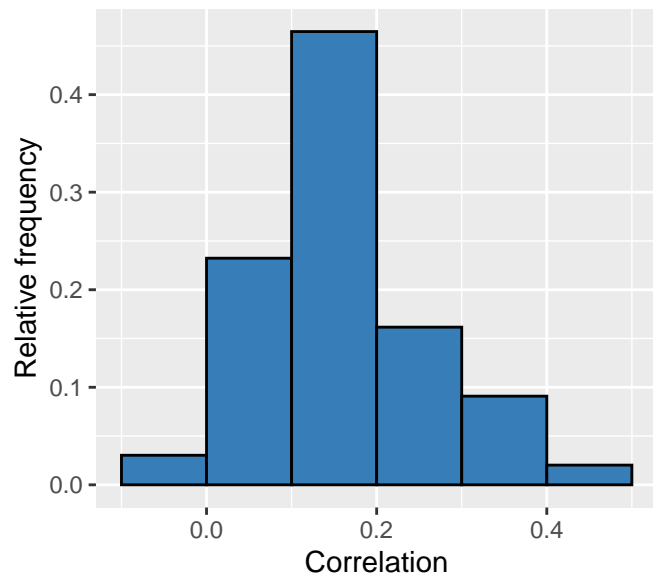

**B**

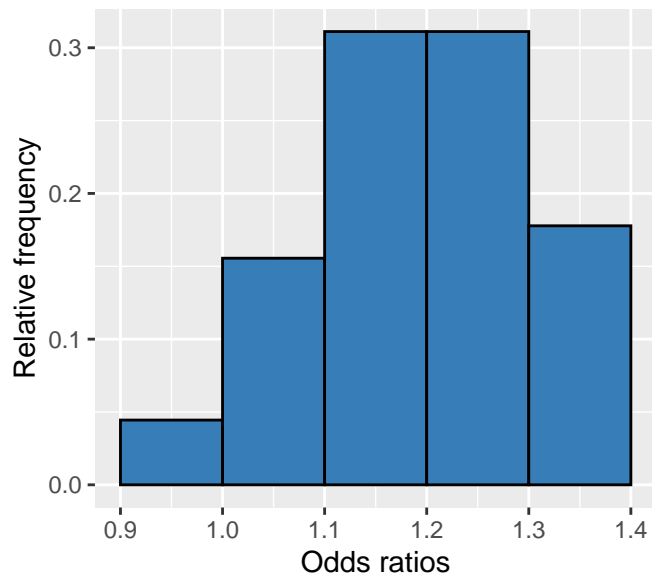

**C**

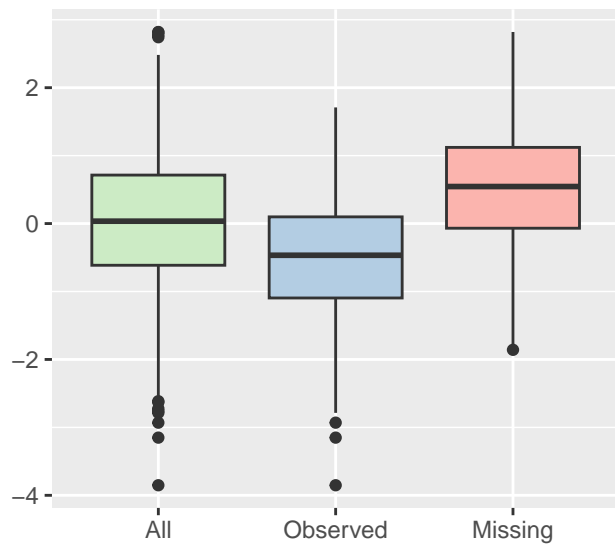

**D**

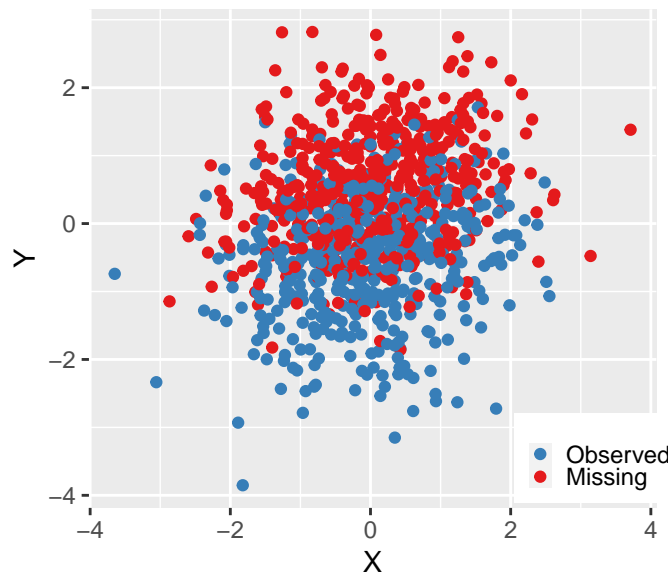

Supplement: Supporting Information 1 [file EMS194352-supplement-Supporting_Information_1.zip › code_resubmitted/sim_study/results/figures/suppfig10_n1000_p100_pmy50_mod.pdf]

$n = 1000$ ,  $p = 100$

**A**

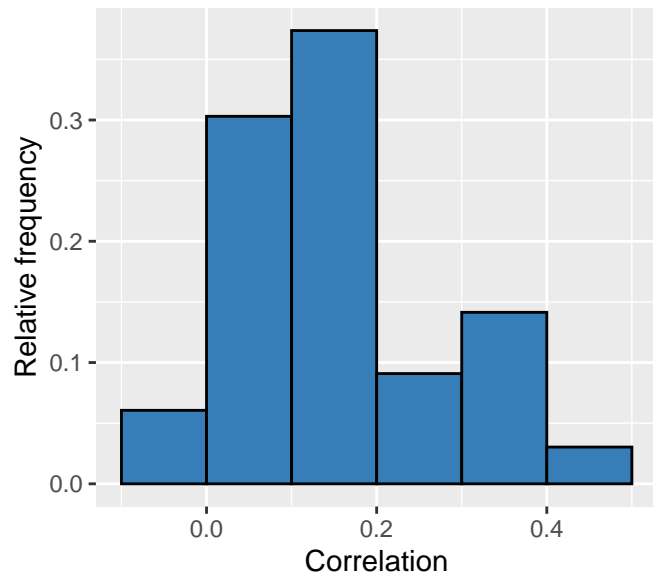

**B**

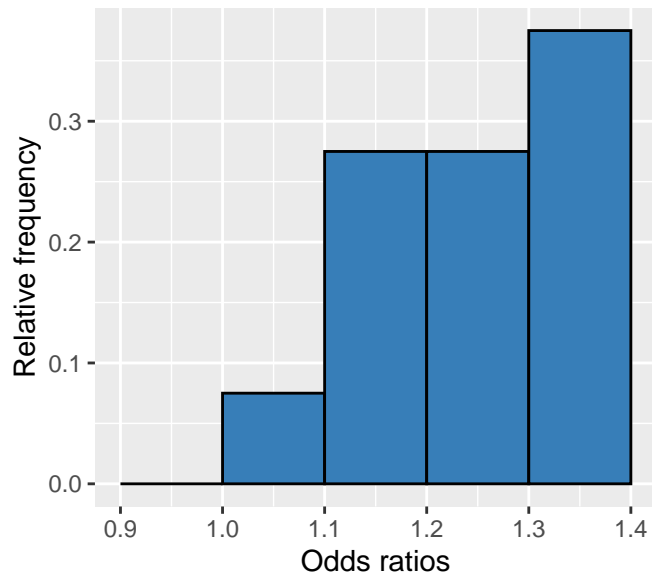

**C**

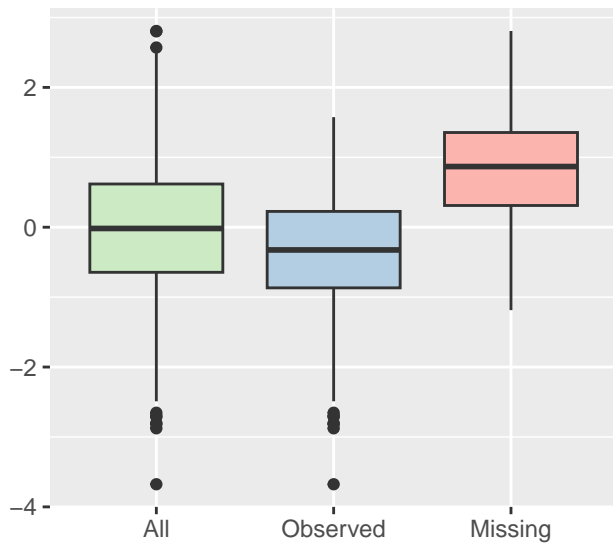

**D**

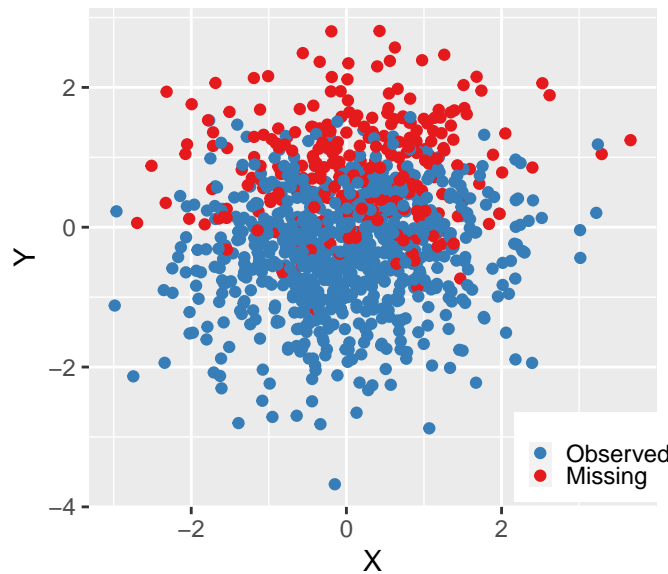

Supplement: Supporting Information 1 [file EMS194352-supplement-Supporting_Information_1.zip › code_resubmitted/sim_study/results/figures/suppfig11_n1000_p100_pmy30_str.pdf]

Average % of variables selected

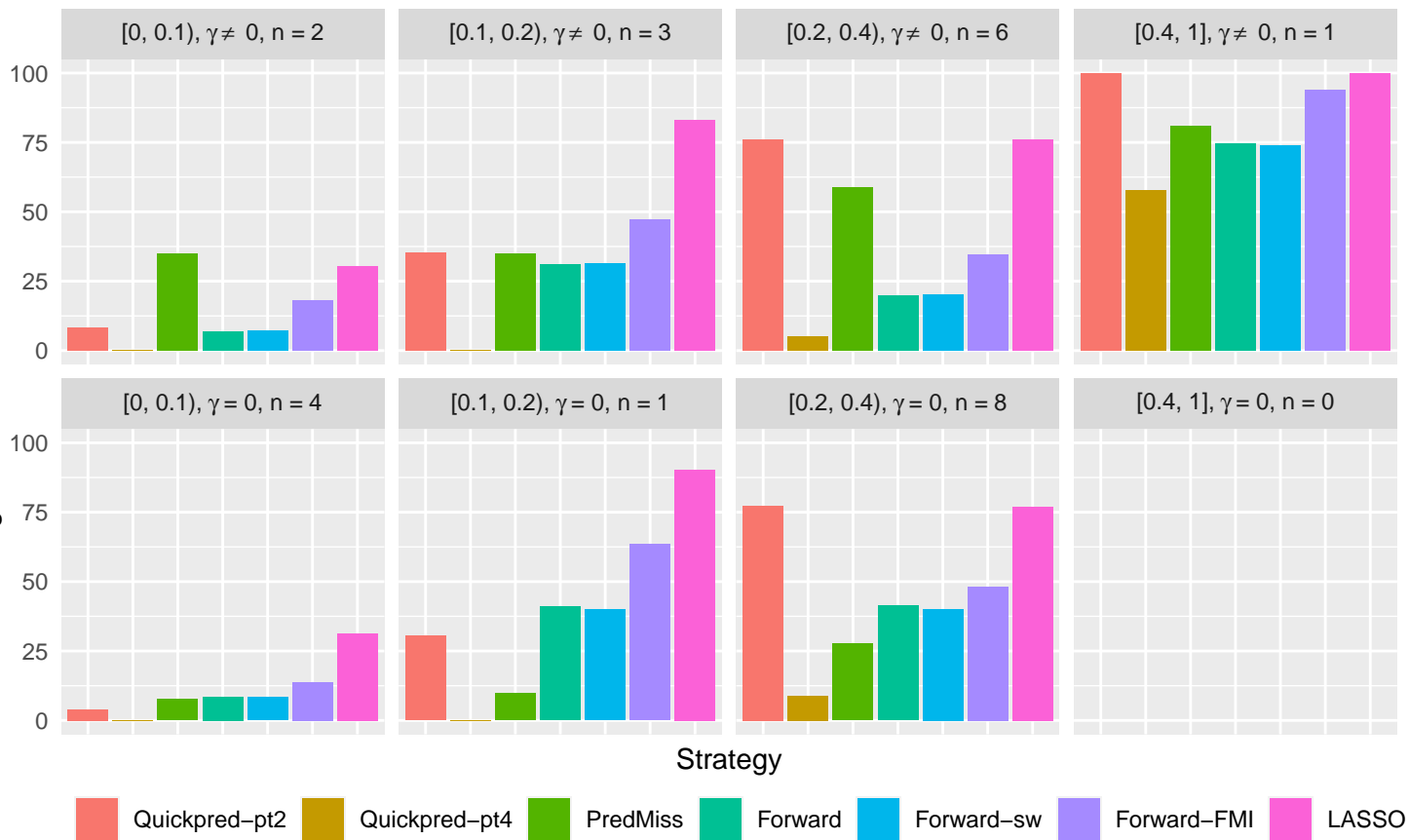

Supplement: Supporting Information 1 [file EMS194352-supplement-Supporting_Information_1.zip › code_resubmitted/sim_study/results/figures/SuppFig13_n250_p25_pmy30_mod.pdf]

Average % of variables selected

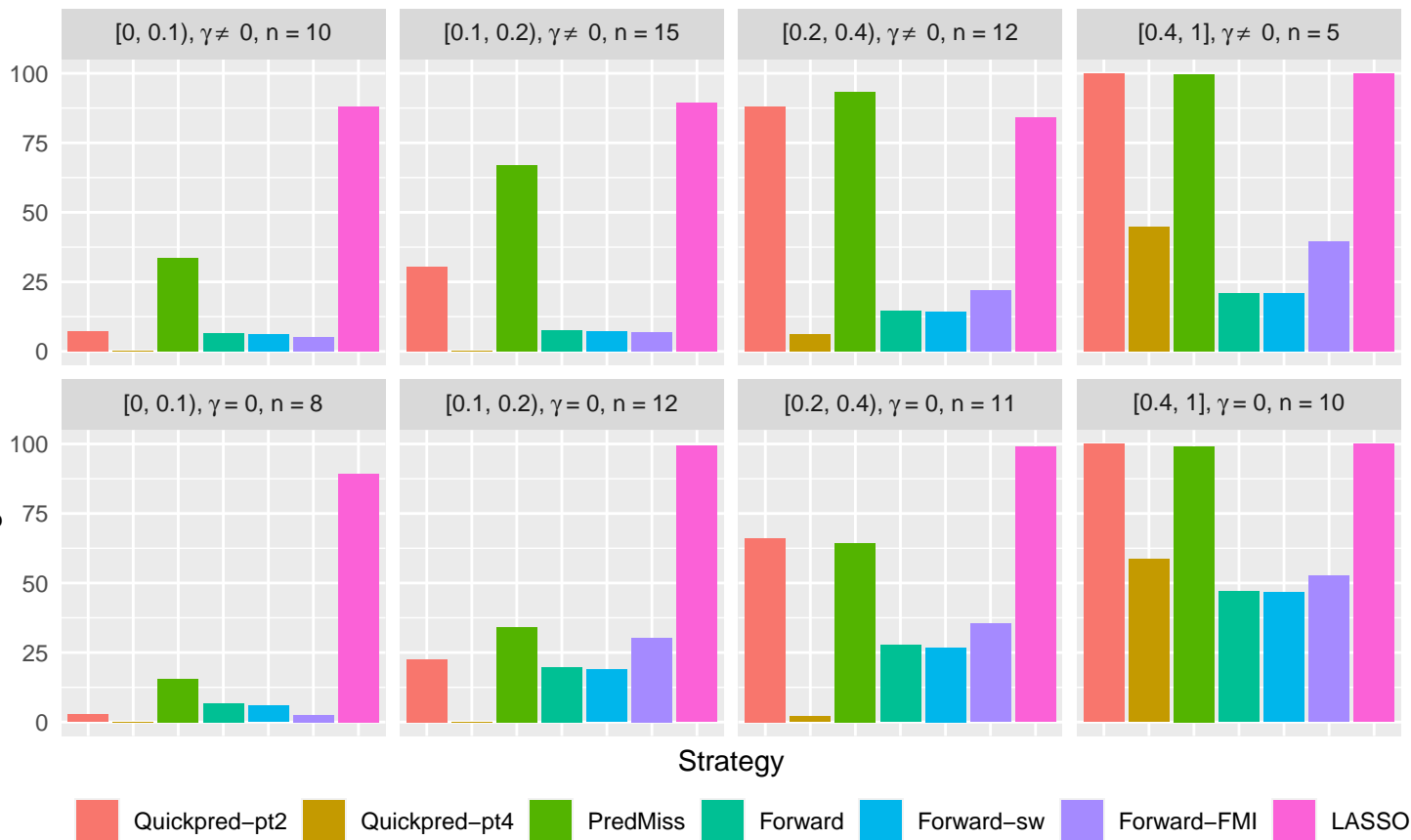

Supplement: Supporting Information 1 [file EMS194352-supplement-Supporting_Information_1.zip › code_resubmitted/sim_study/results/figures/SuppFig14_n250_p83_pmy30_mod.pdf]

Average % of variables selected

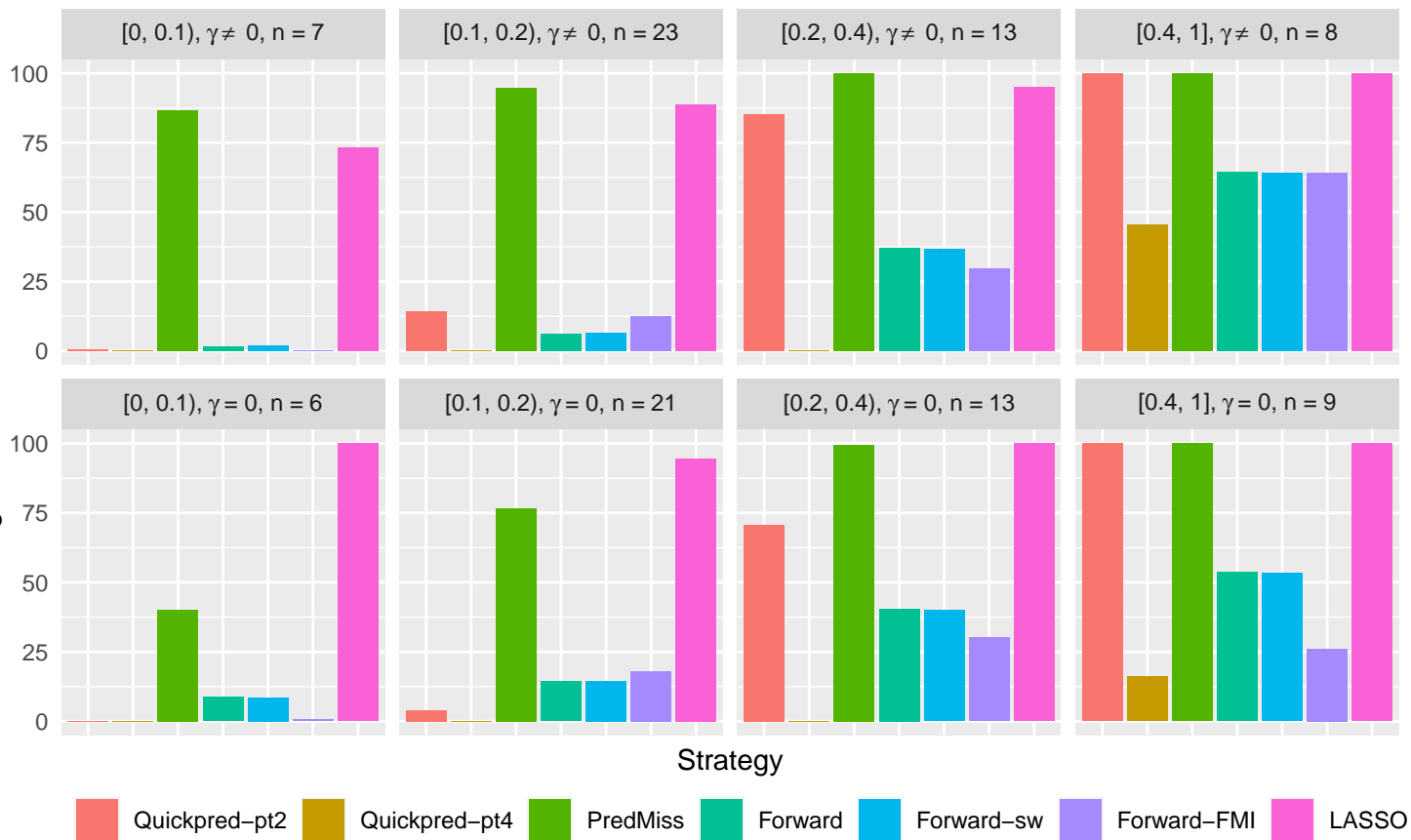

Supplement: Supporting Information 1 [file EMS194352-supplement-Supporting_Information_1.zip › code_resubmitted/sim_study/results/figures/SuppFig15_n1000_p100_pmy30_mod.pdf]

Average % of variables selected

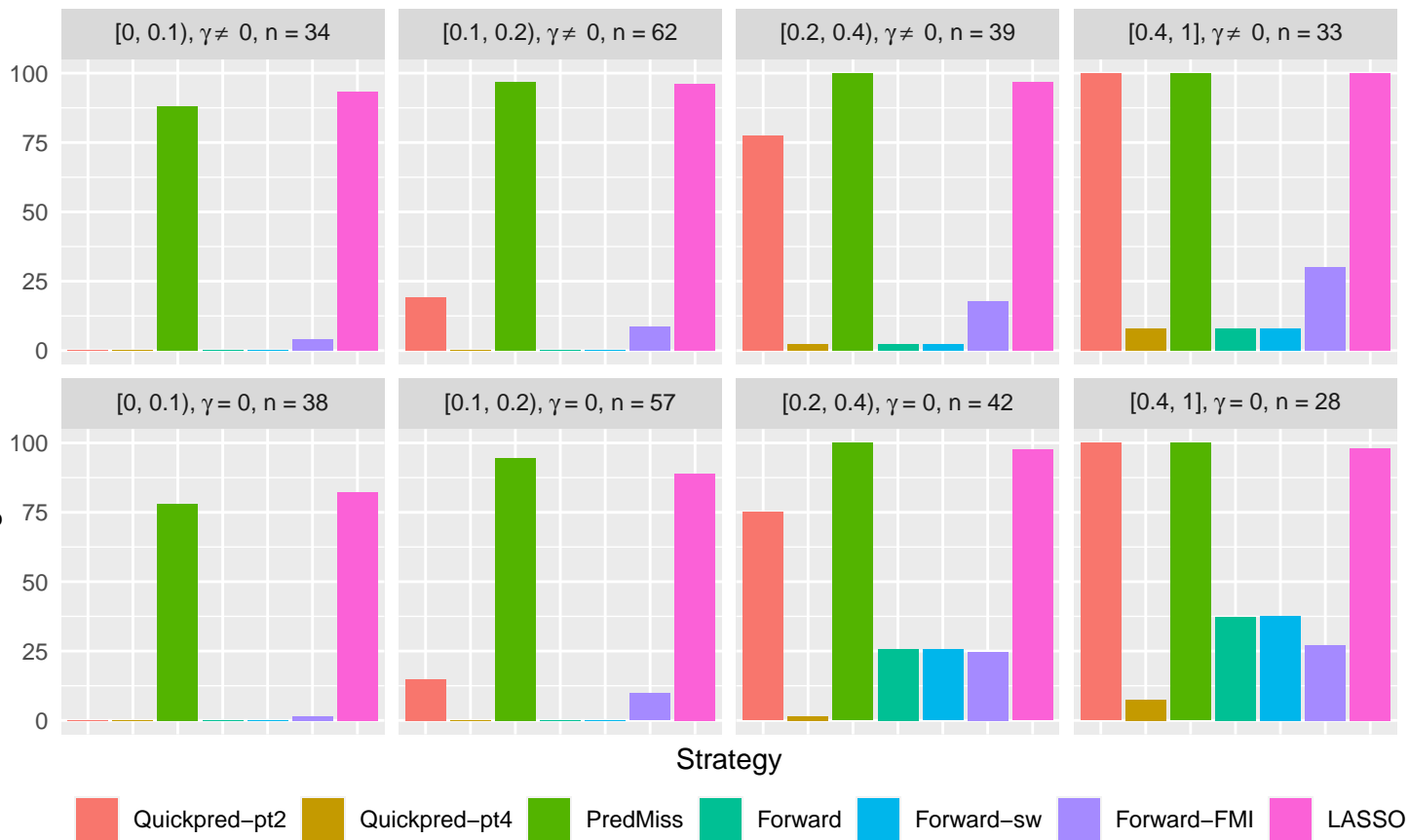

Supplement: Supporting Information 1 [file EMS194352-supplement-Supporting_Information_1.zip › code_resubmitted/sim_study/results/figures/SuppFig16_n1000_p333_pmy30_mod.pdf]

Average % of variables selected

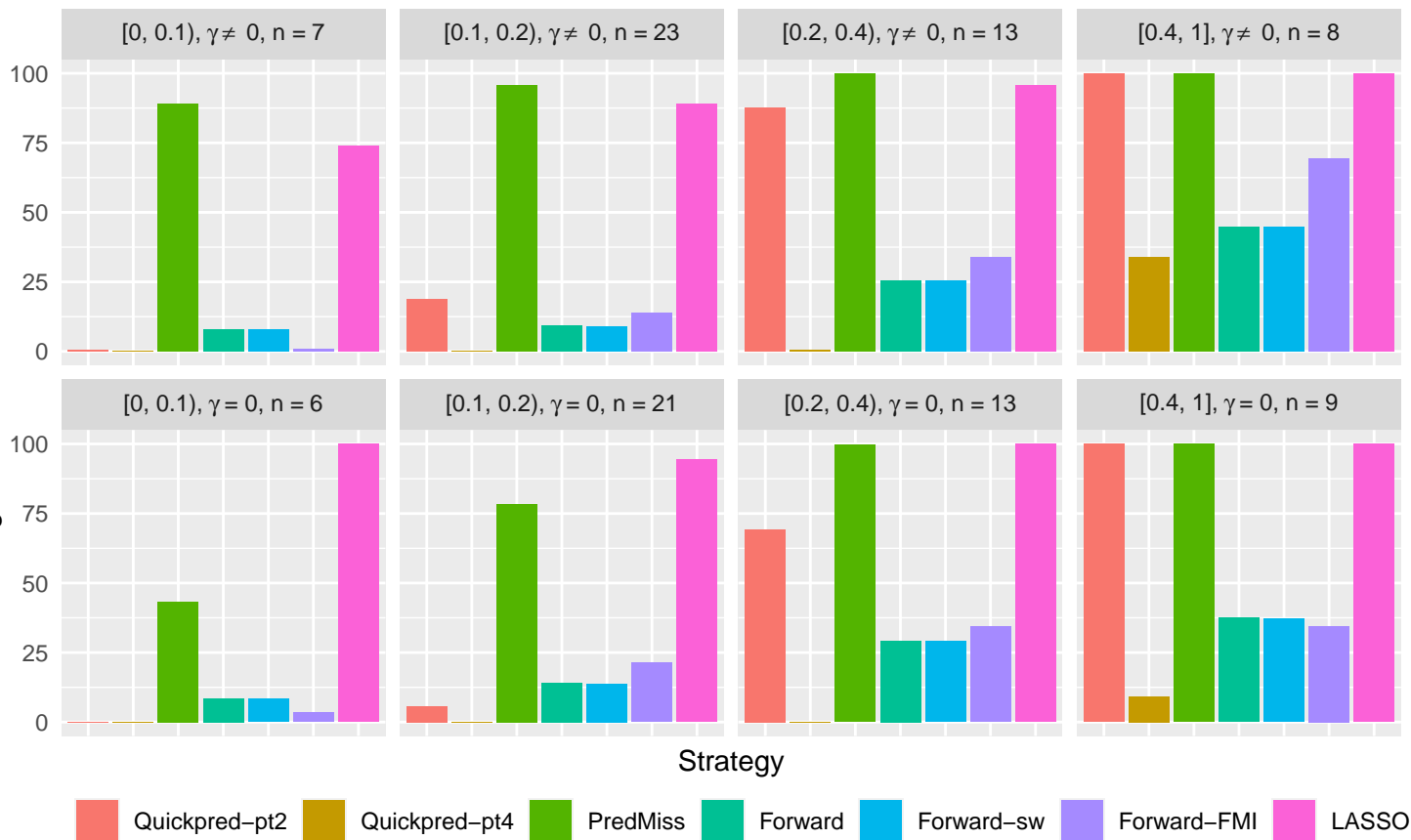

Supplement: Supporting Information 1 [file EMS194352-supplement-Supporting_Information_1.zip › code_resubmitted/sim_study/results/figures/SuppFig17_n1000_p100_pmy50_mod.pdf]

Average % of variables selected

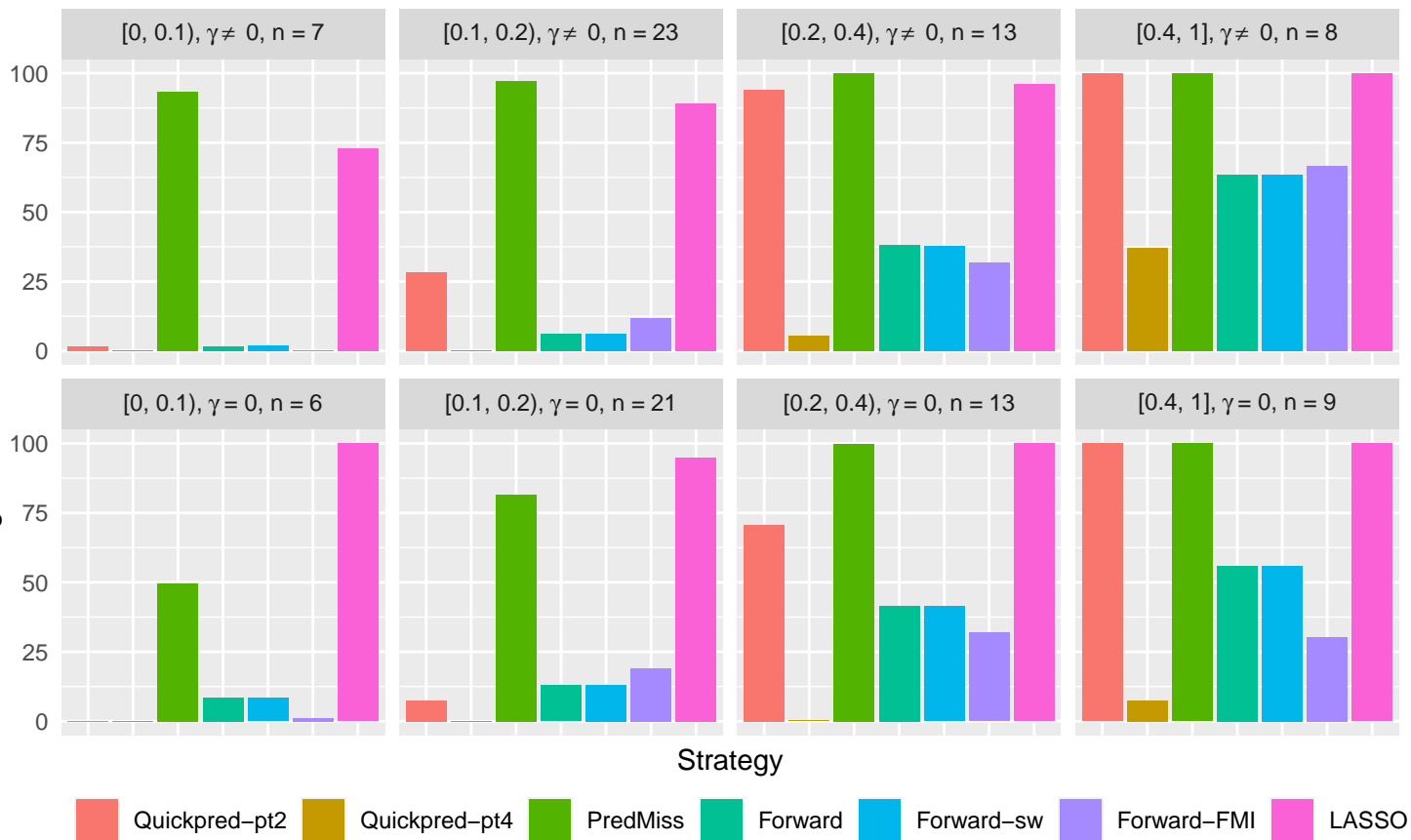

Supplement: Supporting Information 1 [file EMS194352-supplement-Supporting_Information_1.zip › code_resubmitted/sim_study/results/figures/SuppFig18_n1000_p100_pmy30_str.pdf]

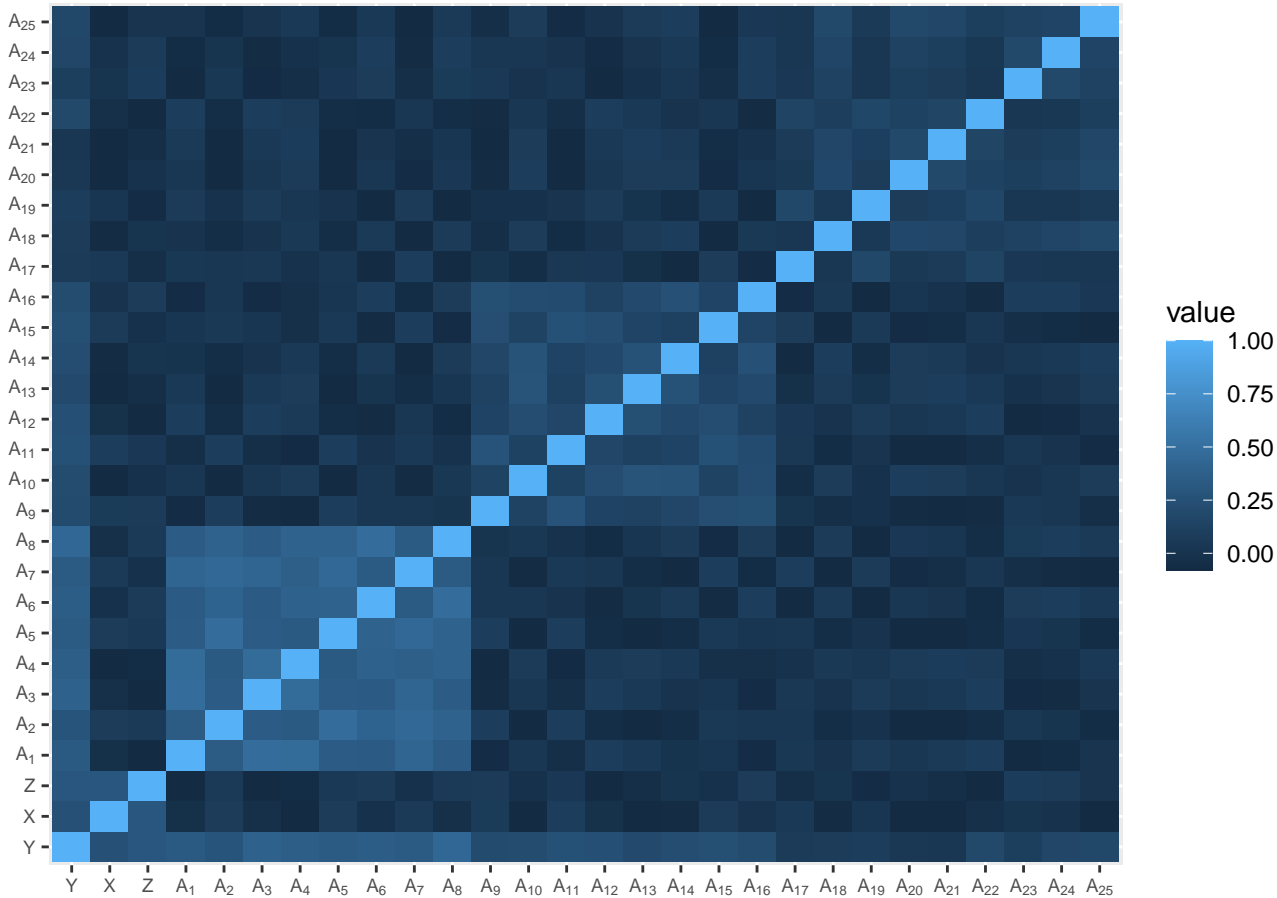

Supplement: Supporting Information 1 [file EMS194352-supplement-Supporting_Information_1.zip › code_resubmitted/sim_study/results/figures/suppfig3.pdf]

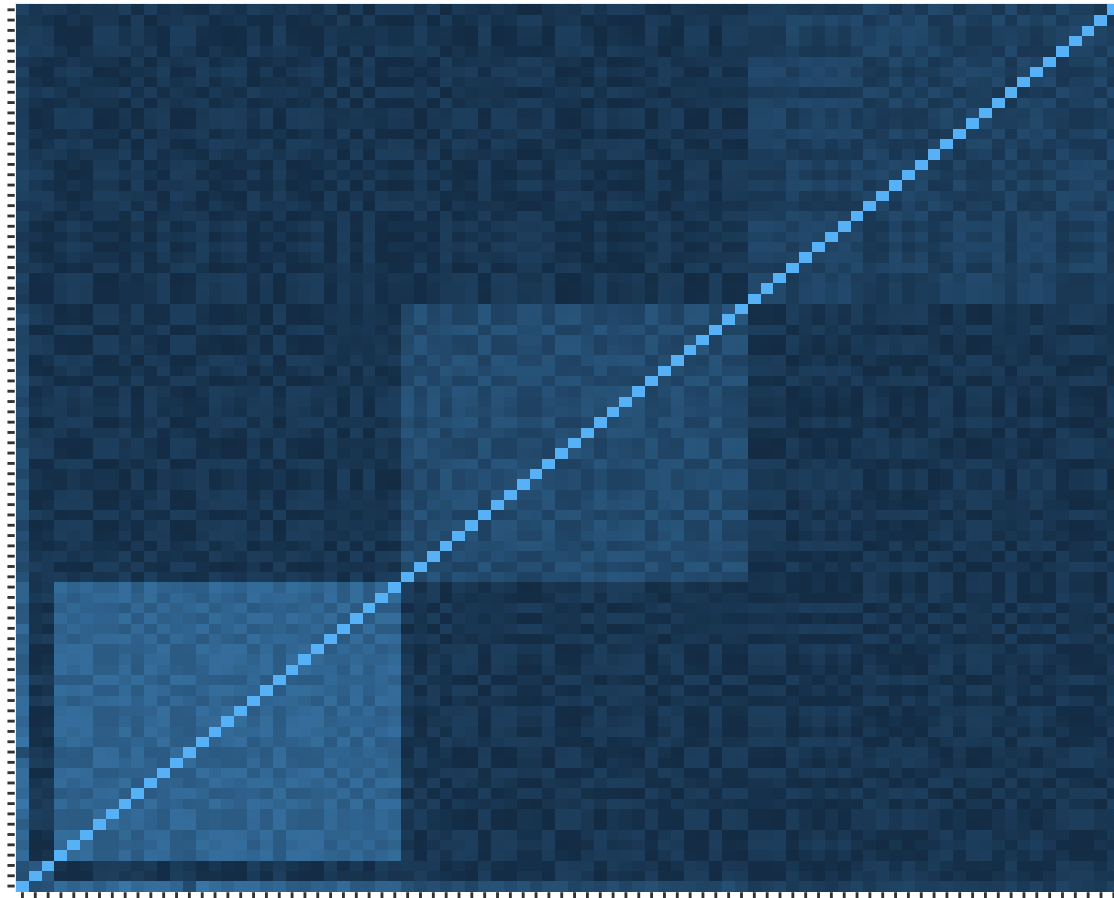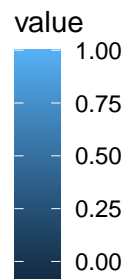

Supplement: Supporting Information 1 [file EMS194352-supplement-Supporting_Information_1.zip › code_resubmitted/sim_study/results/figures/suppfig4.pdf]

$n = 250$ ,  $p = 25$

**A**

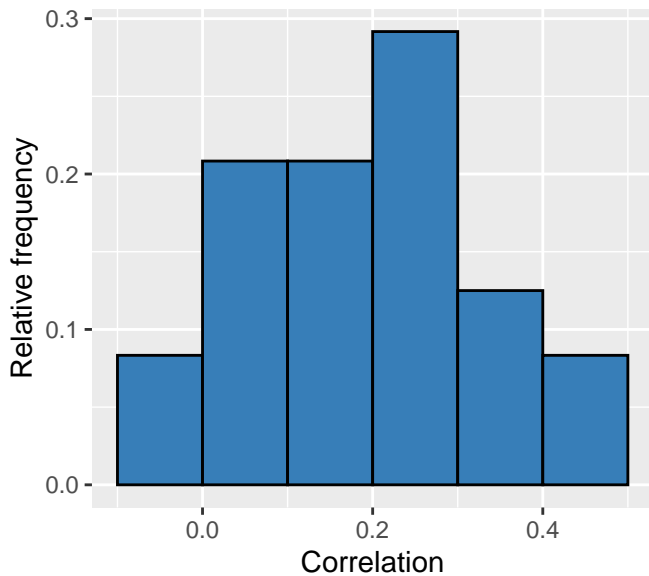

**B**

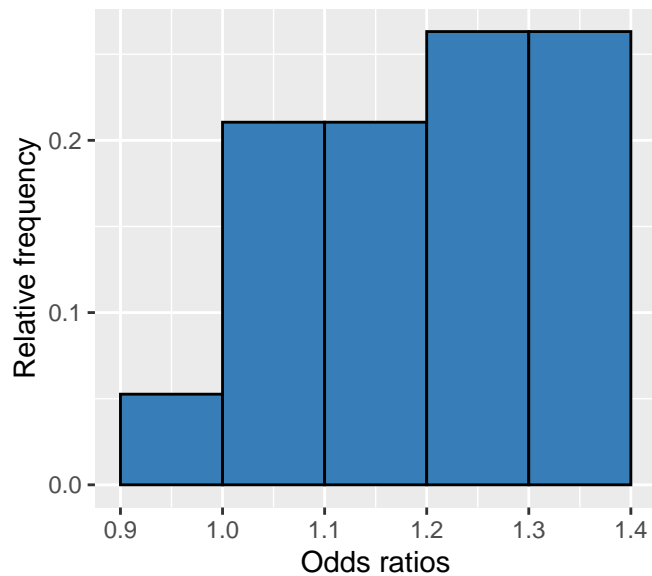

**C**

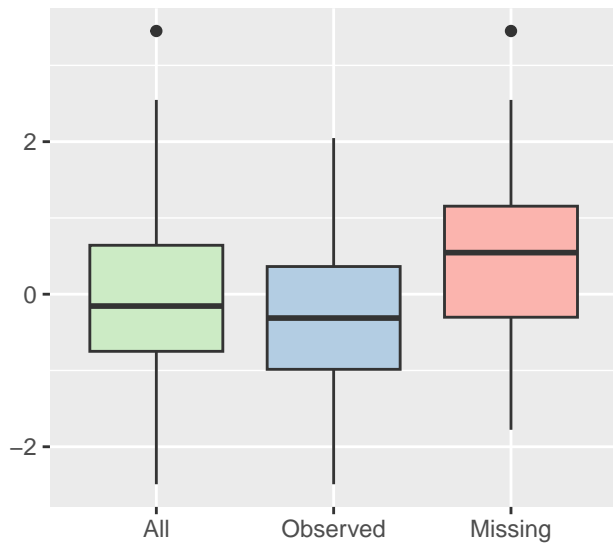

**D**

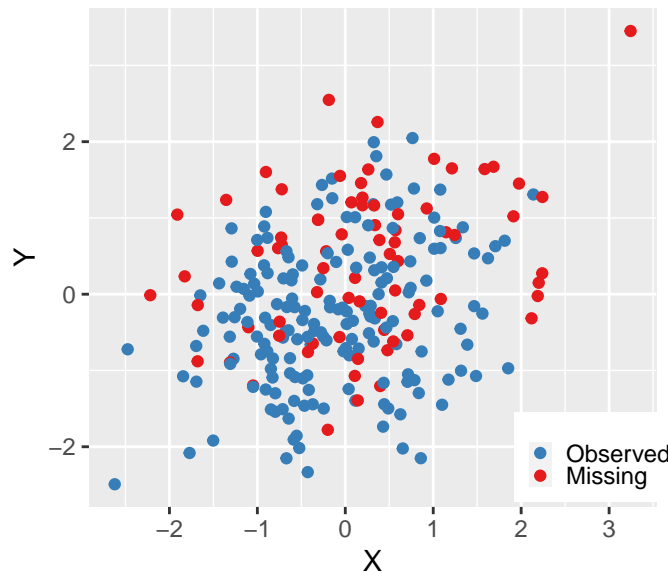

Supplement: Supporting Information 1 [file EMS194352-supplement-Supporting_Information_1.zip › code_resubmitted/sim_study/results/figures/suppfig6_n250_p25_pmy30_mod.pdf]

$n = 250$ ,  $p = 83$

**A**

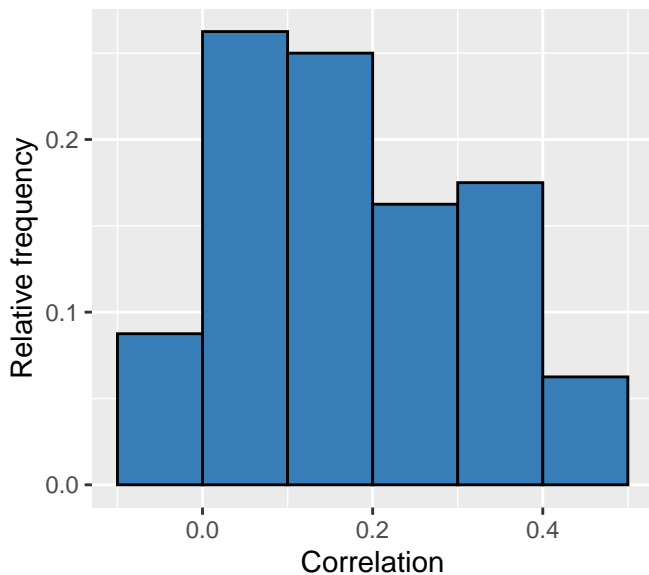

**B**

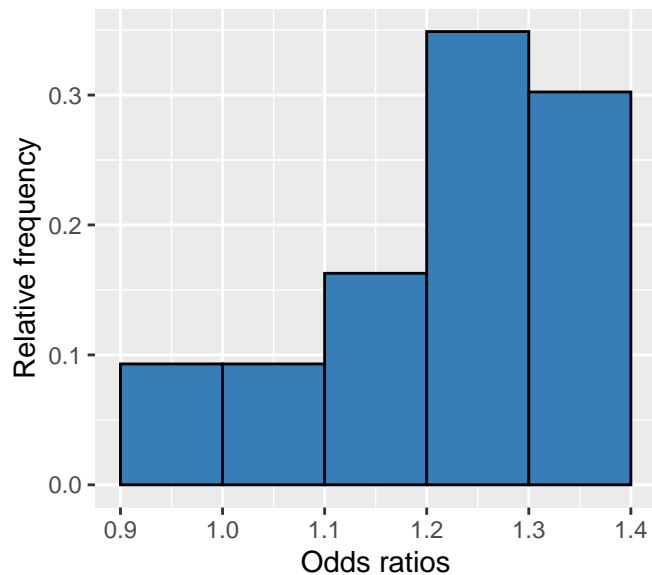

**C**

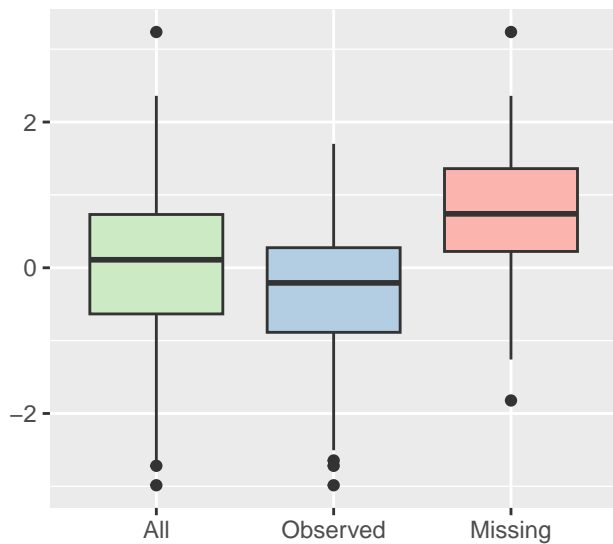

**D**

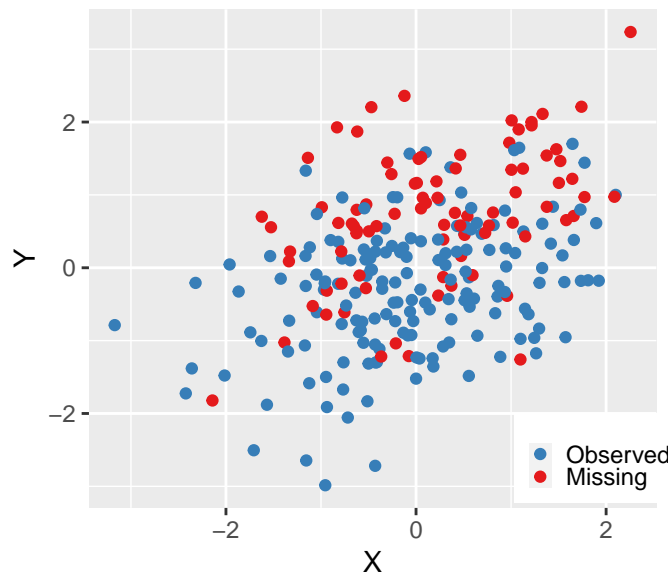

Supplement: Supporting Information 1 [file EMS194352-supplement-Supporting_Information_1.zip › code_resubmitted/sim_study/results/figures/suppfig7_n250_p83_pmy30_mod.pdf]

$n = 1000$ ,  $p = 100$

**A**

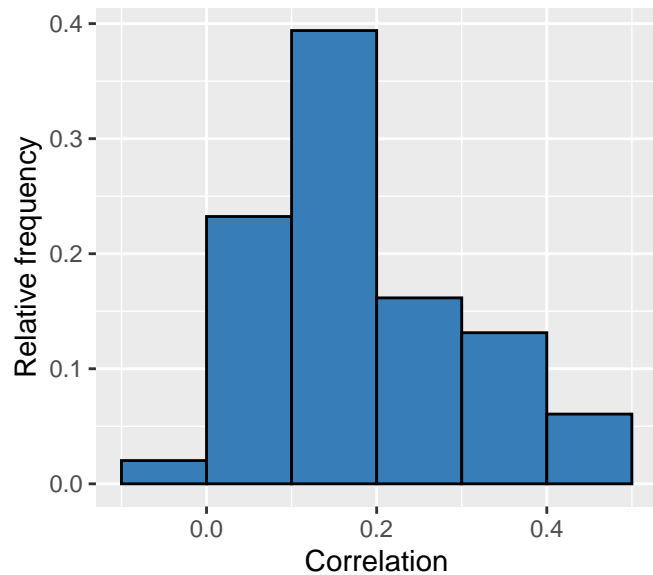

**B**

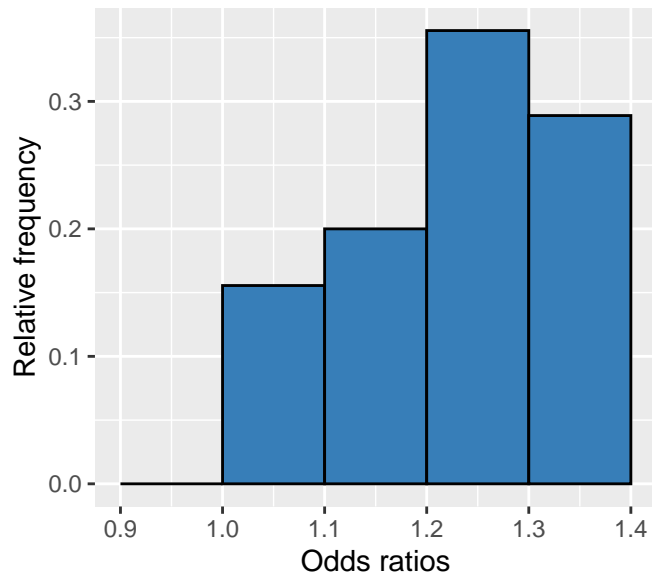

**C**

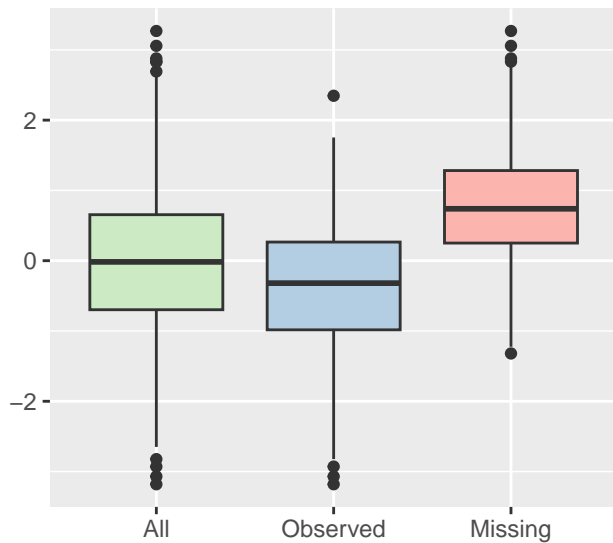

**D**

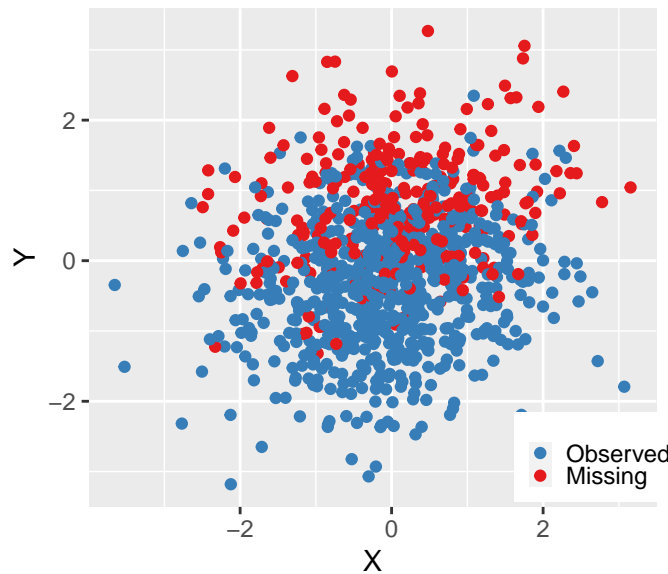

Supplement: Supporting Information 1 [file EMS194352-supplement-Supporting_Information_1.zip › code_resubmitted/sim_study/results/figures/suppfig8_n1000_p100_pmy30_mod.pdf]

n = 1000 , p = 333

**A**

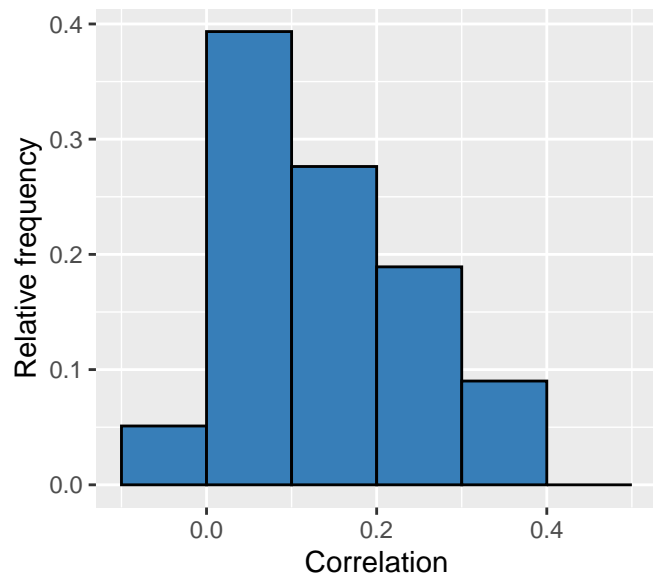

**B**

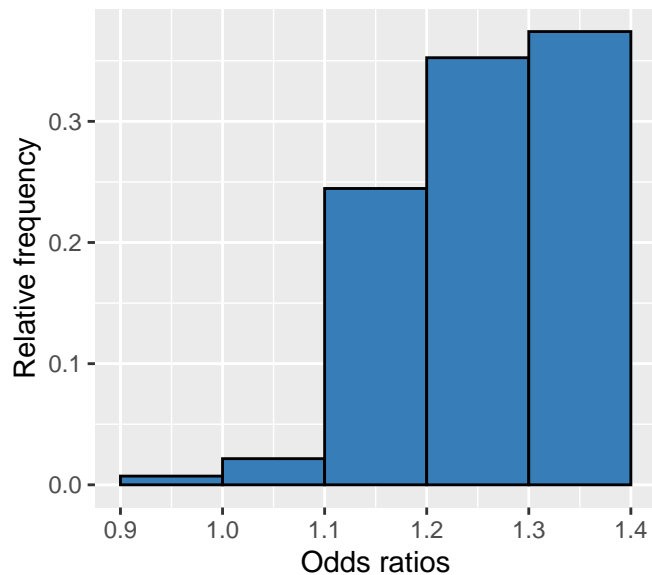

**C**

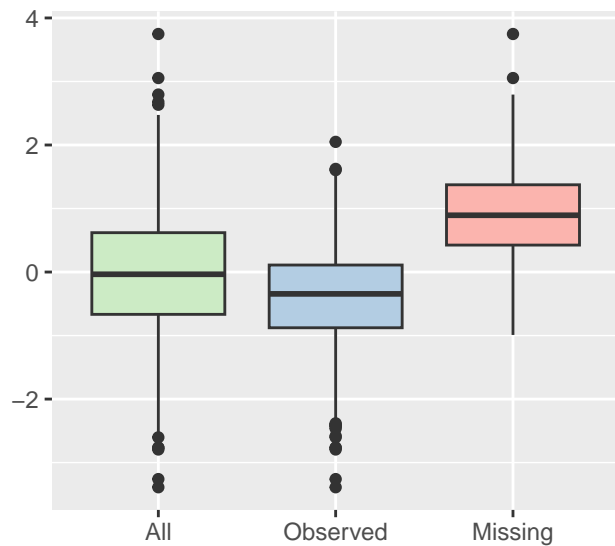

**D**

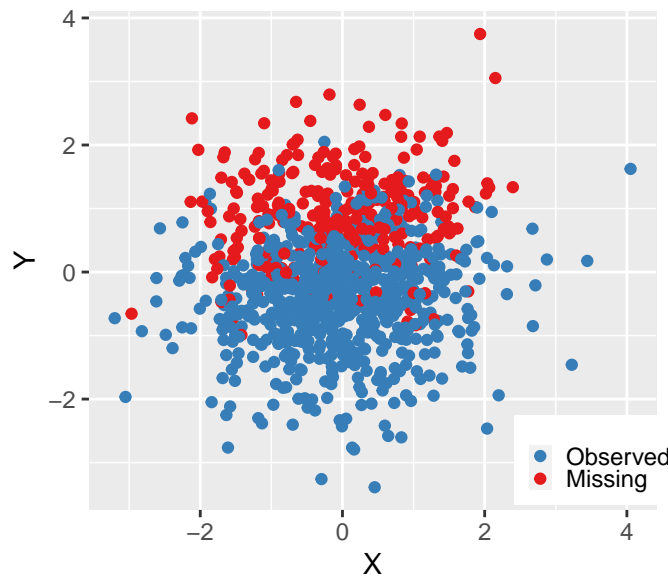

Supplement: Supporting Information 1 [file EMS194352-supplement-Supporting_Information_1.zip › code_resubmitted/sim_study/results/figures/suppfig9_n1000_p333_pmy30_mod.pdf]

# Bias

# Empirical SE

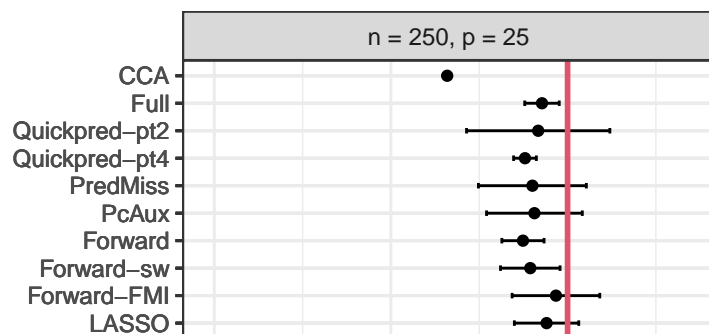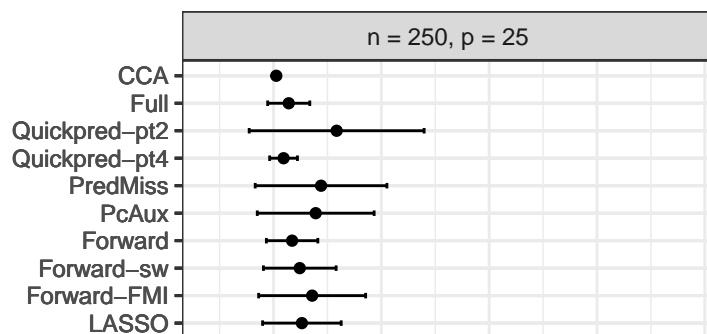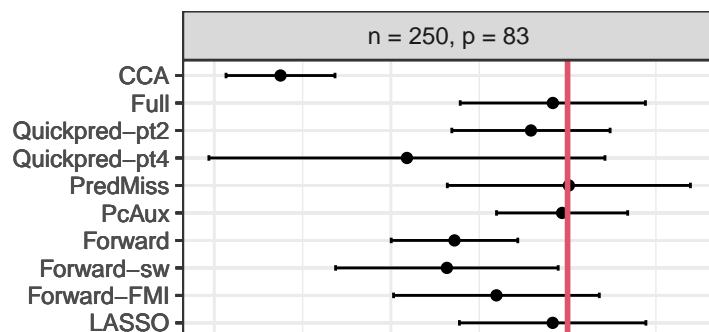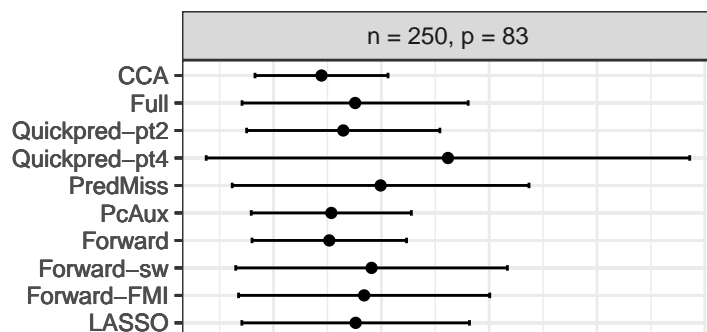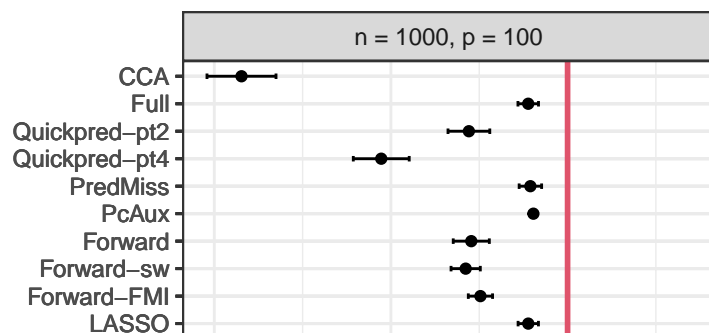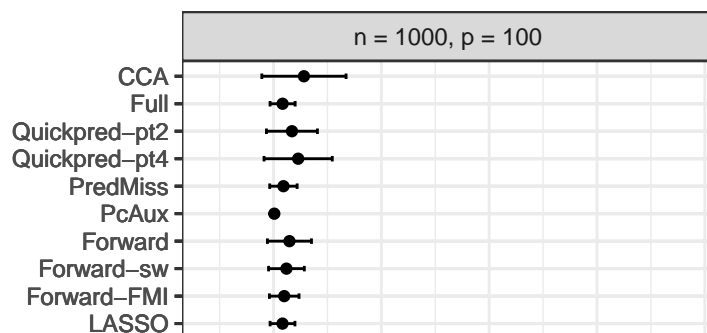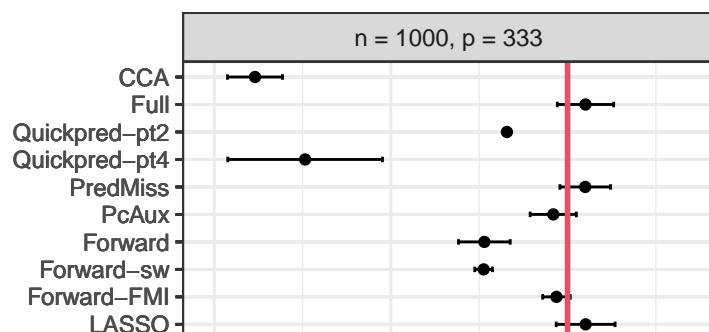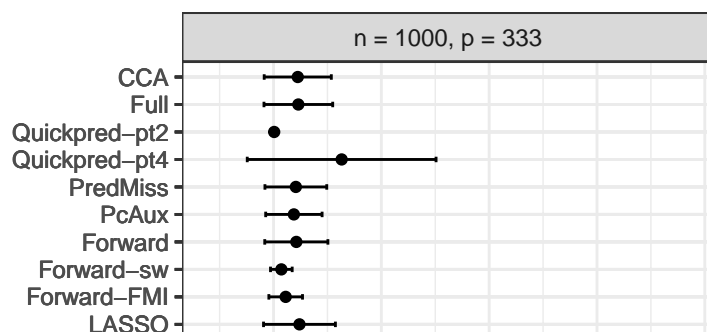

Supplement: Supporting Information 1 [file EMS194352-supplement-Supporting_Information_1.zip › code_resubmitted/sim_study_MWE/results/figures/Fig1_meany.pdf]

# Bias

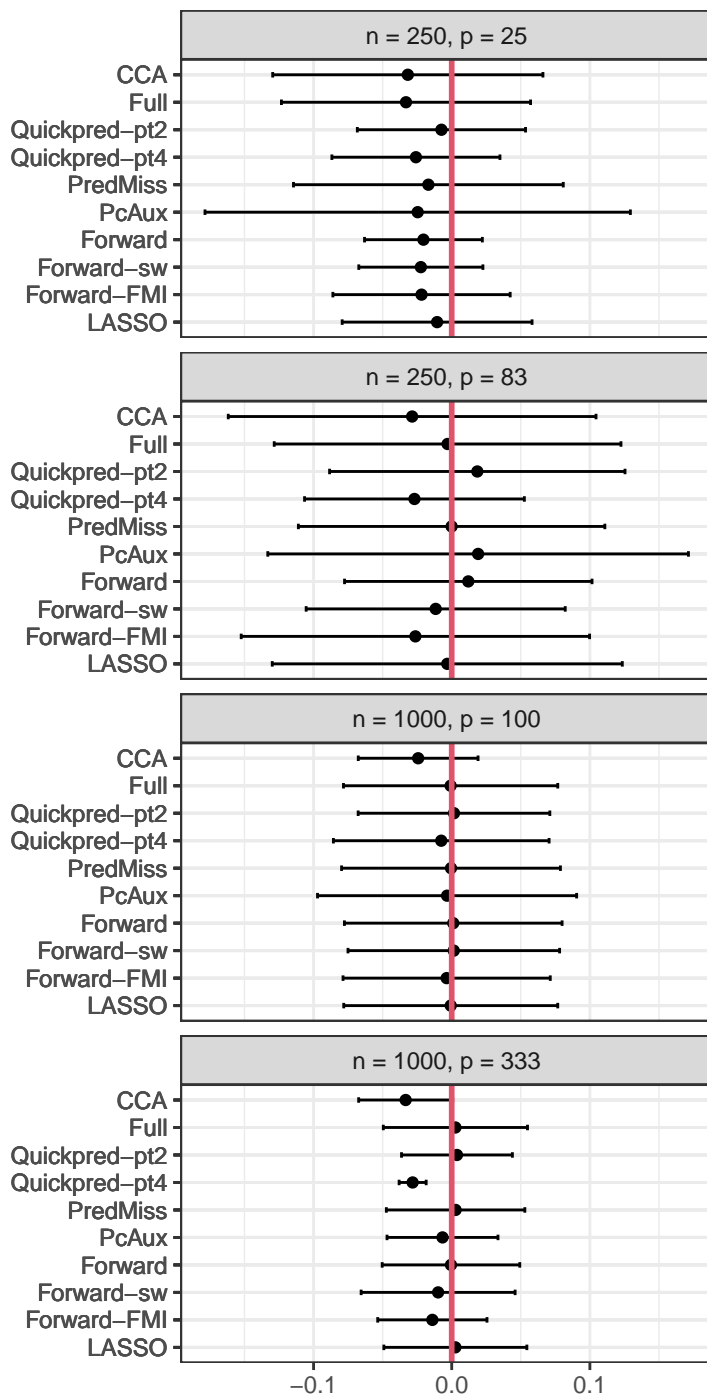

# Empirical SE

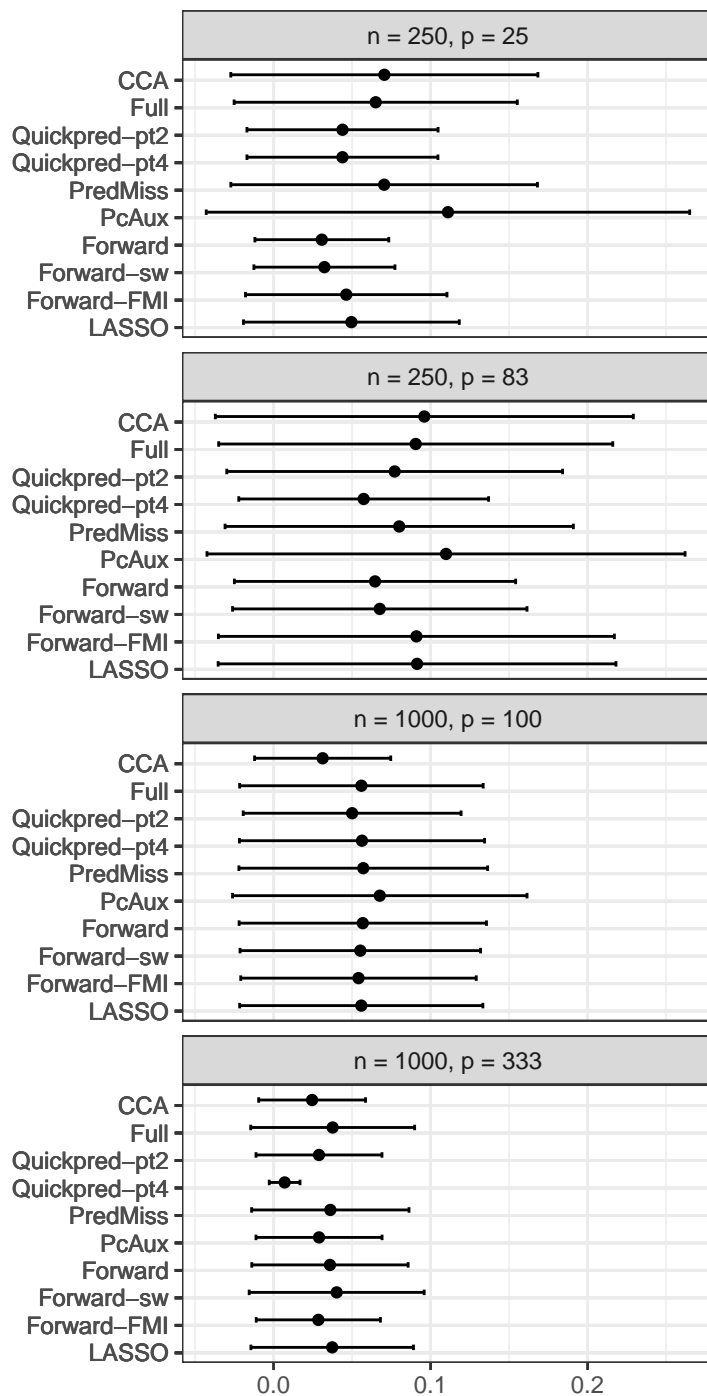

Supplement: Supporting Information 1 [file EMS194352-supplement-Supporting_Information_1.zip › code_resubmitted/sim_study_MWE/results/figures/Fig2_betax.pdf]

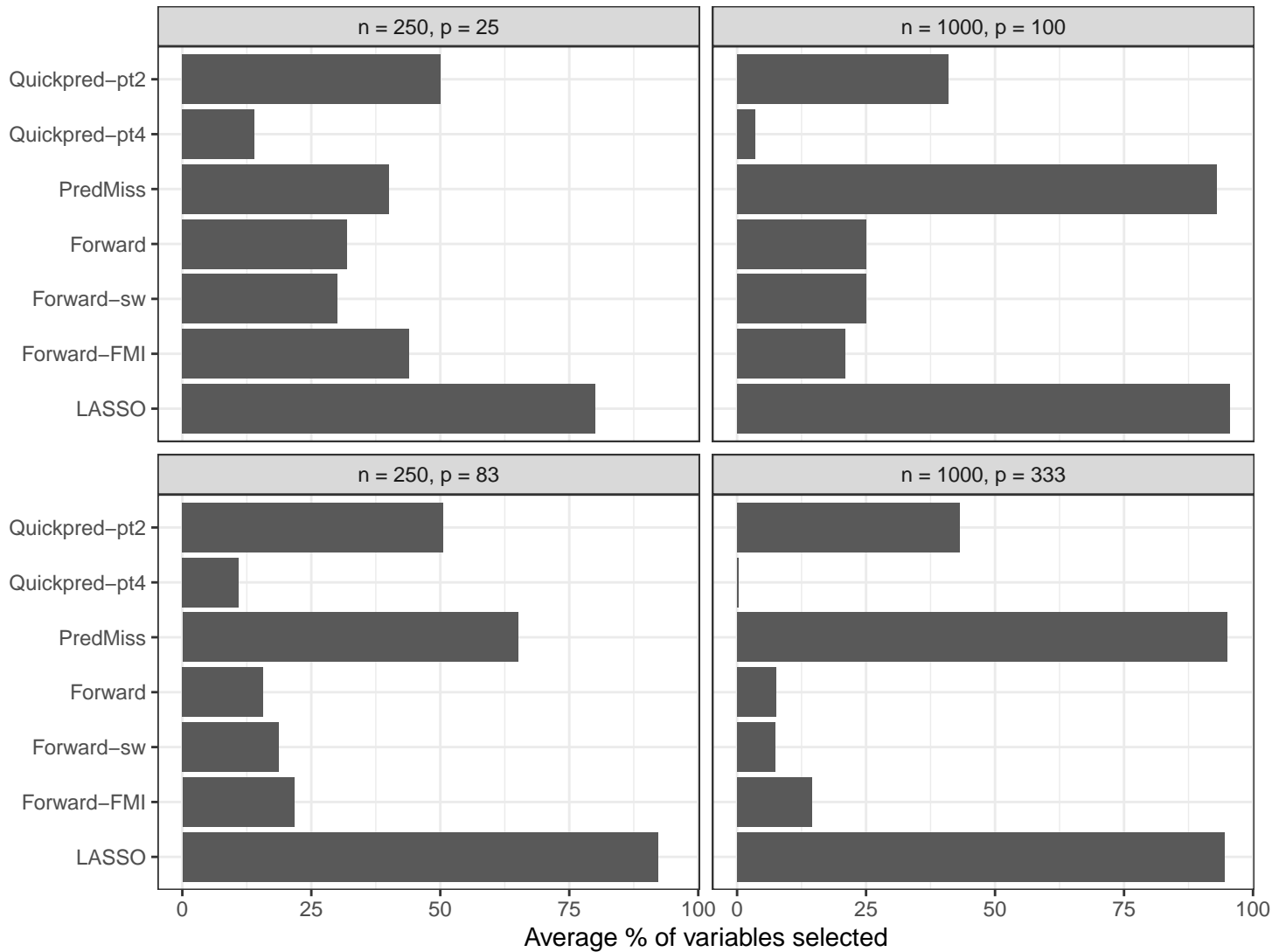

Supplement: Supporting Information 1 [file EMS194352-supplement-Supporting_Information_1.zip › code_resubmitted/sim_study_MWE/results/figures/Fig3_TotalAuxVars.pdf]

$n = 1000$ ,  $p = 100$

**A**

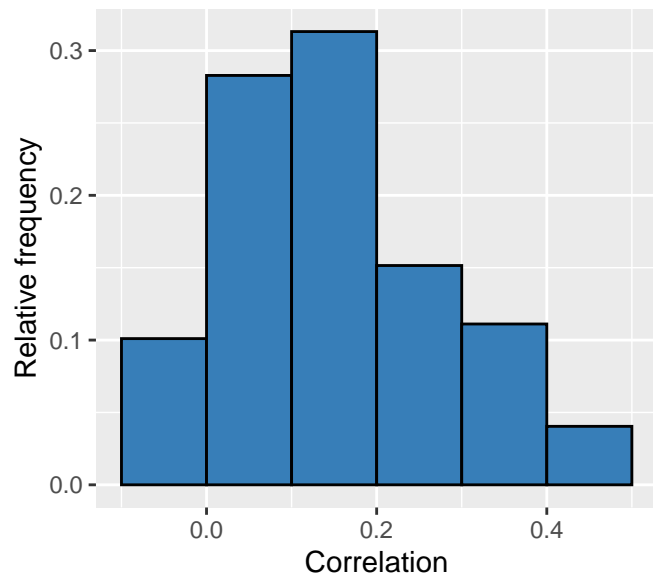

**B**

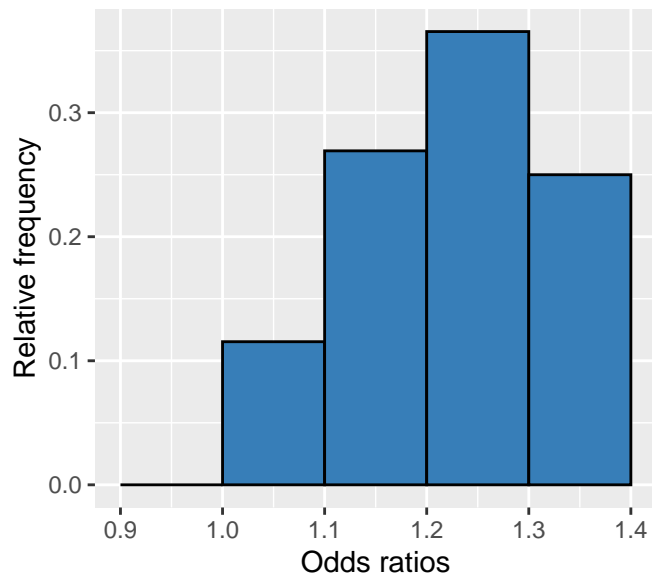

**C**

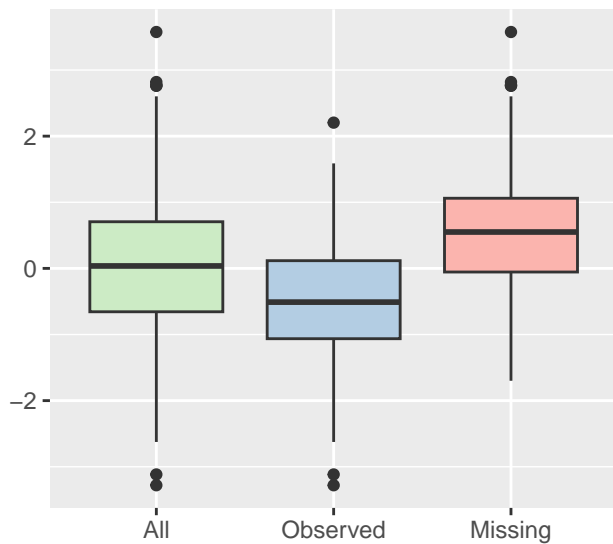

**D**

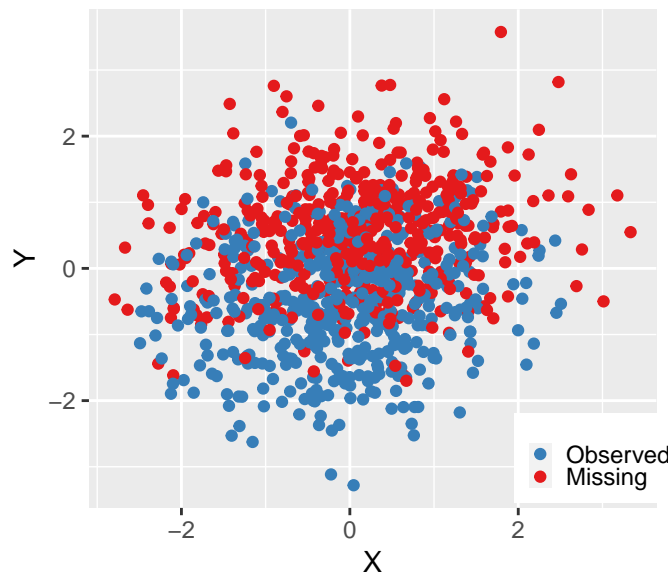

Supplement: Supporting Information 1 [file EMS194352-supplement-Supporting_Information_1.zip › code_resubmitted/sim_study_MWE/results/figures/suppfig10_n1000_p100_pmy50_mod.pdf]

$n = 1000$ ,  $p = 100$

**A**

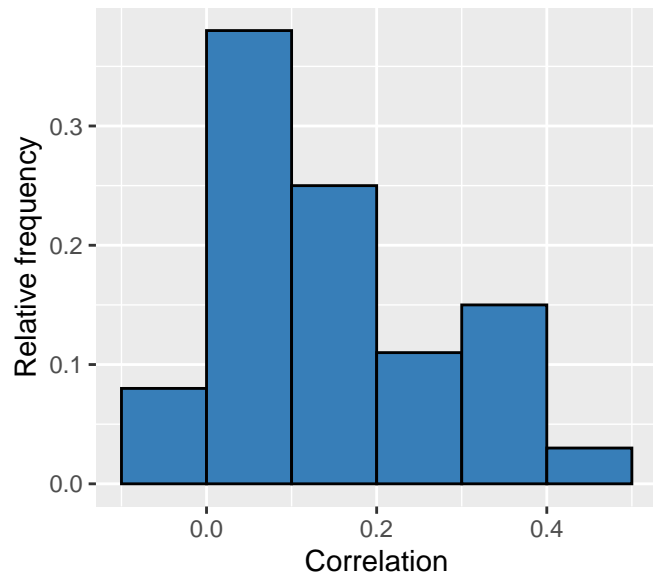

**B**

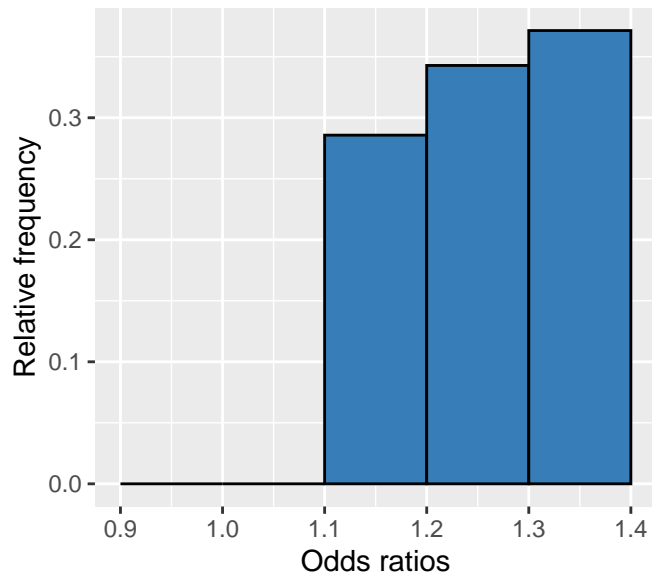

**C**

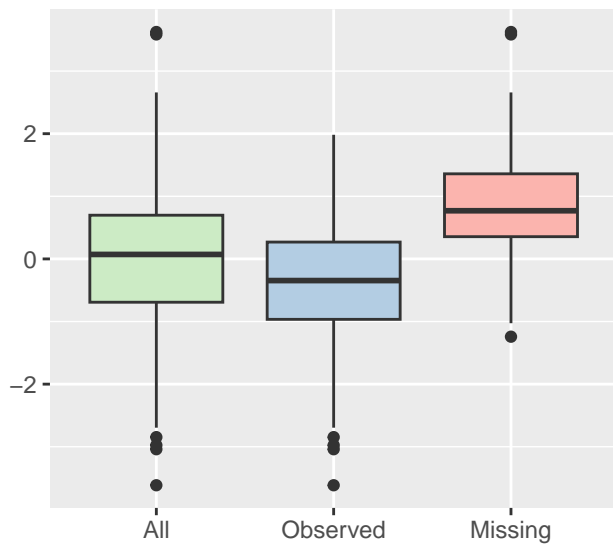

**D**

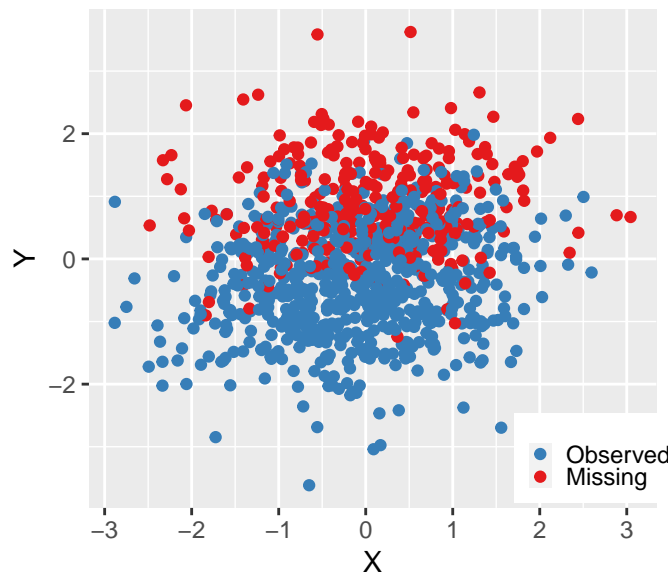

Supplement: Supporting Information 1 [file EMS194352-supplement-Supporting_Information_1.zip › code_resubmitted/sim_study_MWE/results/figures/suppfig11_n1000_p100_pmy30_str.pdf]

Average % of variables selected

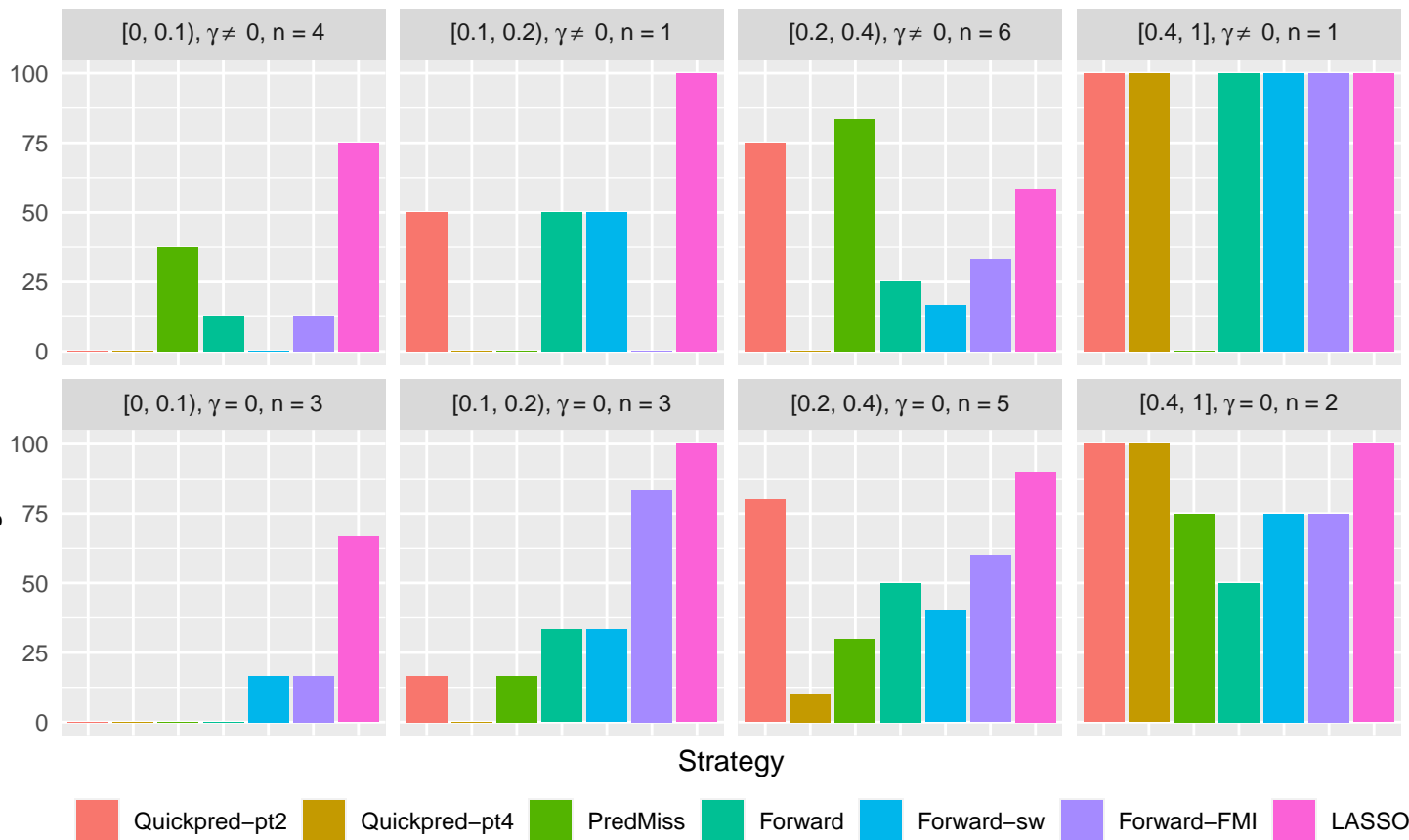

Supplement: Supporting Information 1 [file EMS194352-supplement-Supporting_Information_1.zip › code_resubmitted/sim_study_MWE/results/figures/SuppFig13_n250_p25_pmy30_mod.pdf]

Average % of variables selected

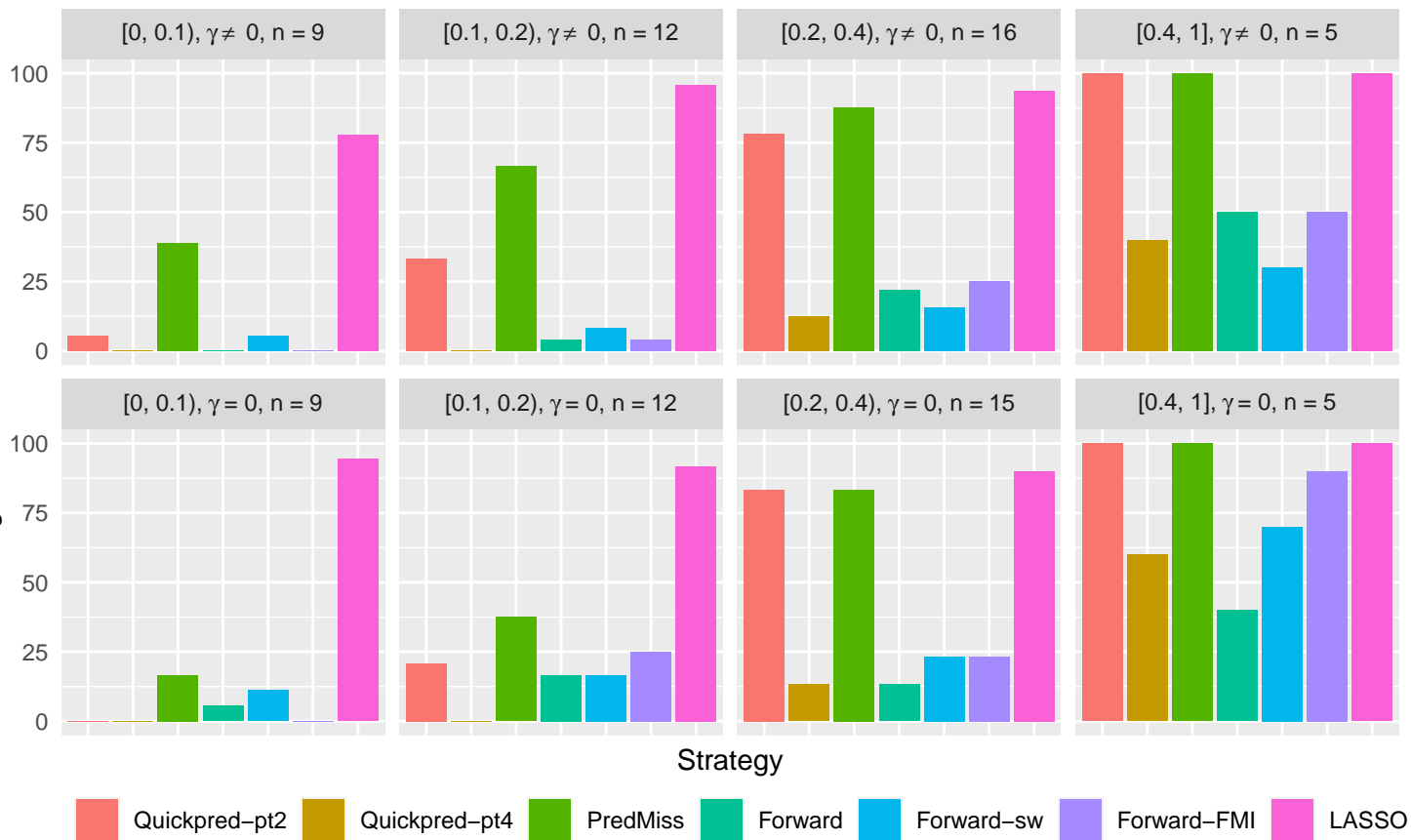

Supplement: Supporting Information 1 [file EMS194352-supplement-Supporting_Information_1.zip › code_resubmitted/sim_study_MWE/results/figures/SuppFig14_n250_p83_pmy30_mod.pdf]

Average % of variables selected

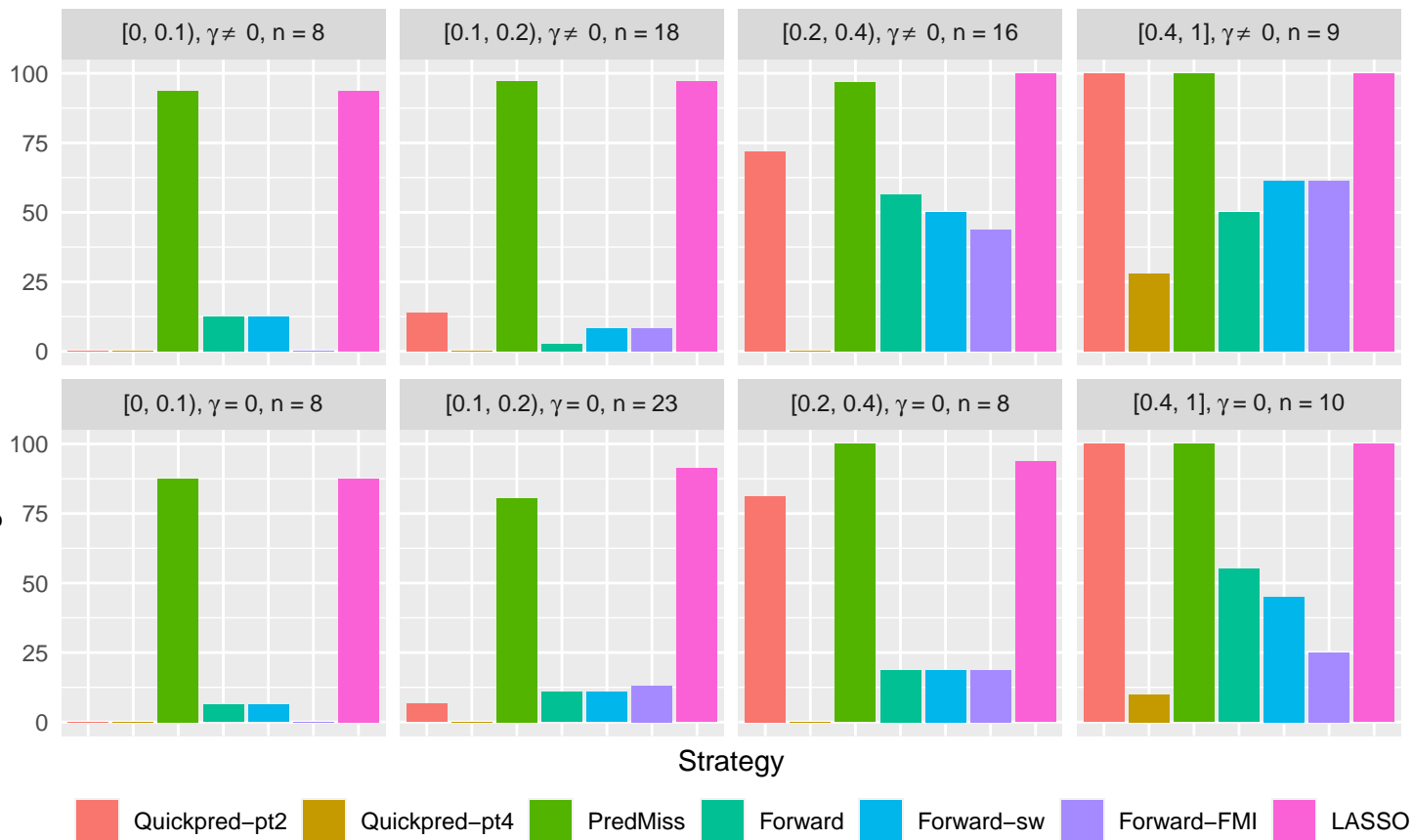

Supplement: Supporting Information 1 [file EMS194352-supplement-Supporting_Information_1.zip › code_resubmitted/sim_study_MWE/results/figures/SuppFig15_n1000_p100_pmy30_mod.pdf]

Average % of variables selected

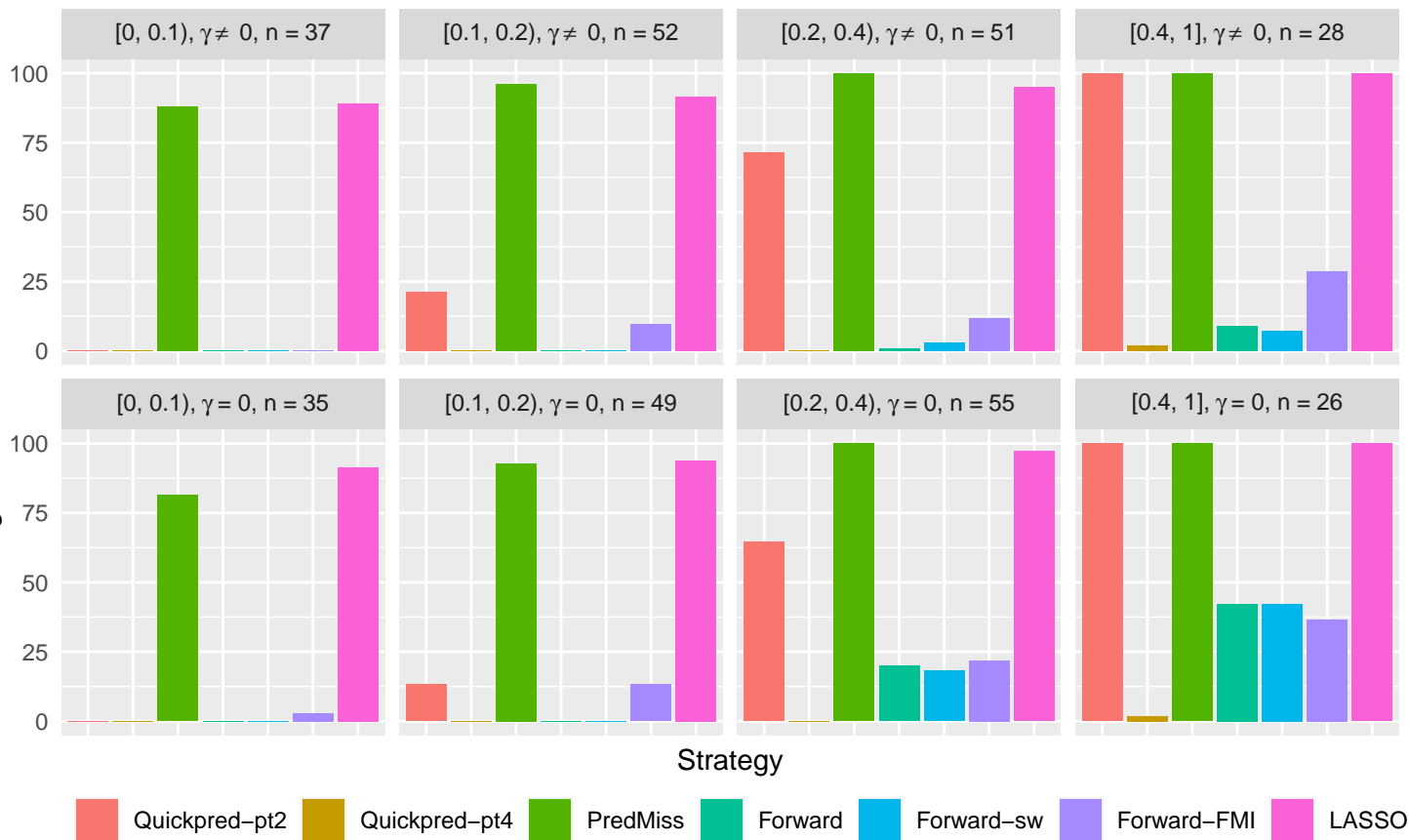

Supplement: Supporting Information 1 [file EMS194352-supplement-Supporting_Information_1.zip › code_resubmitted/sim_study_MWE/results/figures/SuppFig16_n1000_p333_pmy30_mod.pdf]

Average % of variables selected

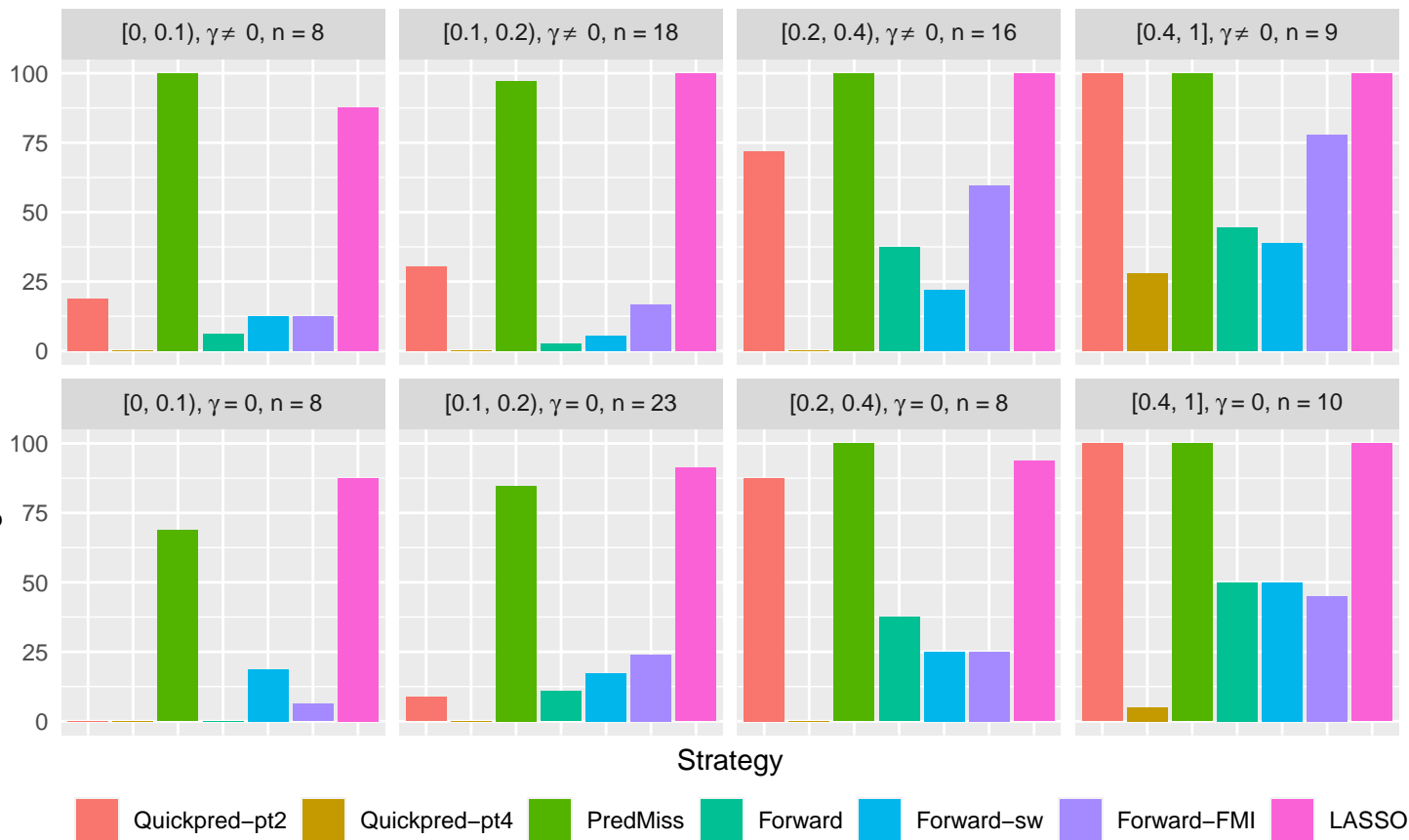

Supplement: Supporting Information 1 [file EMS194352-supplement-Supporting_Information_1.zip › code_resubmitted/sim_study_MWE/results/figures/SuppFig17_n1000_p100_pmy50_mod.pdf]

Average % of variables selected

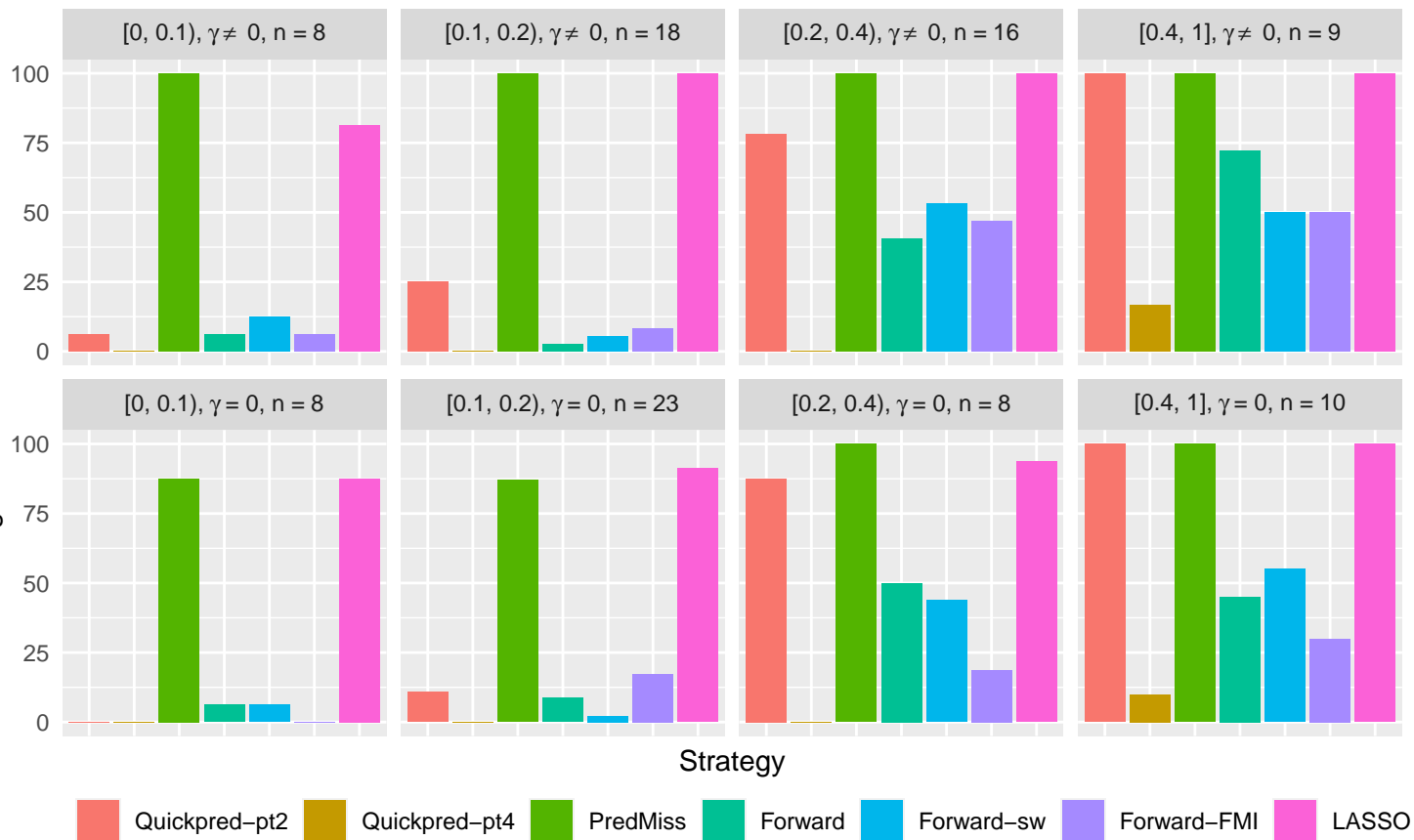

Supplement: Supporting Information 1 [file EMS194352-supplement-Supporting_Information_1.zip › code_resubmitted/sim_study_MWE/results/figures/SuppFig18_n1000_p100_pmy30_str.pdf]

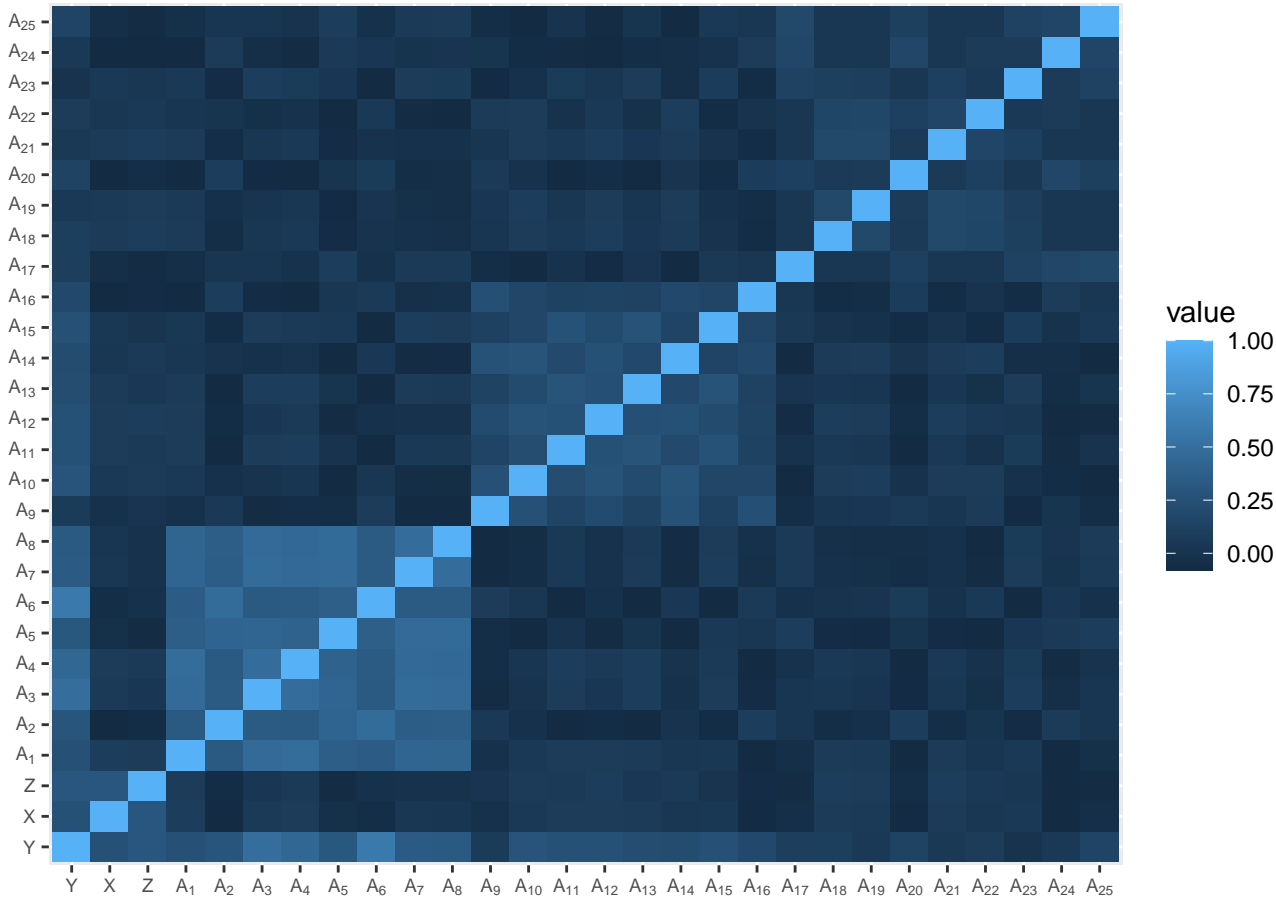

Supplement: Supporting Information 1 [file EMS194352-supplement-Supporting_Information_1.zip › code_resubmitted/sim_study_MWE/results/figures/suppfig3.pdf]

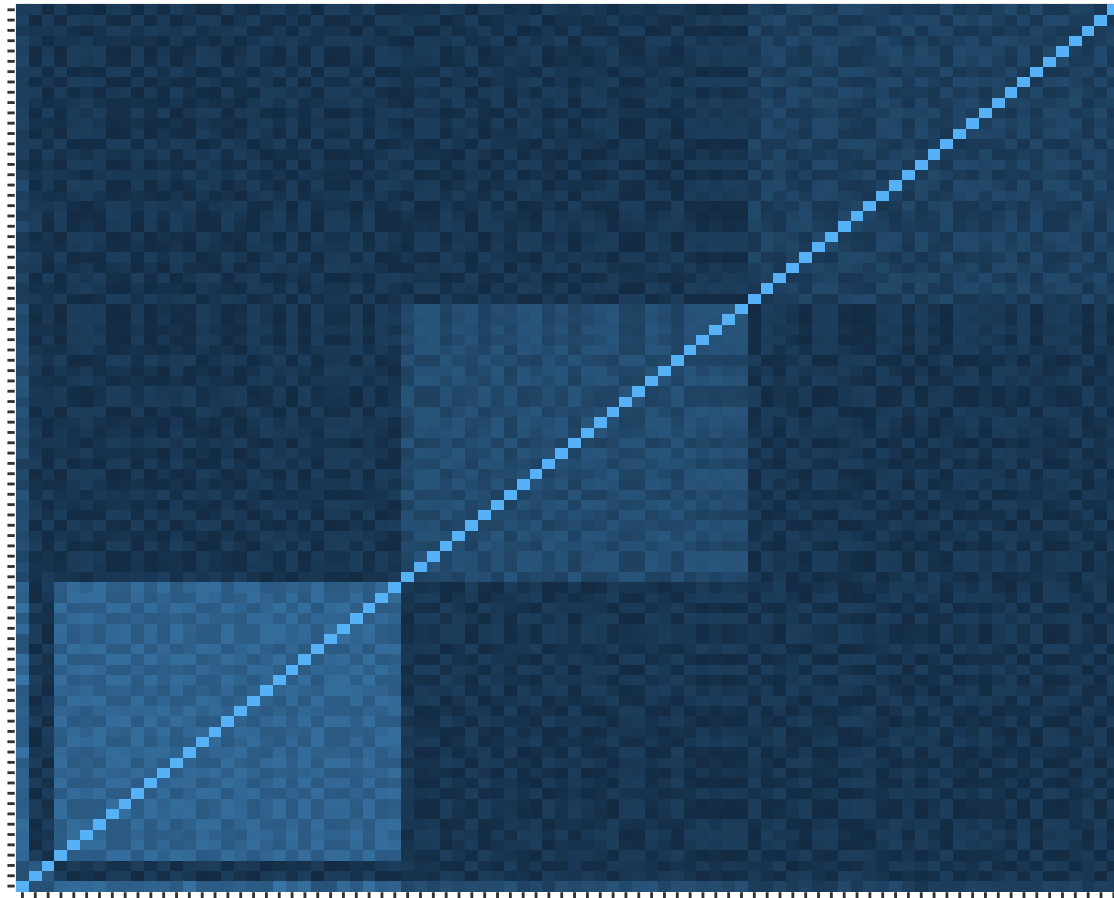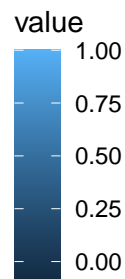

Supplement: Supporting Information 1 [file EMS194352-supplement-Supporting_Information_1.zip › code_resubmitted/sim_study_MWE/results/figures/suppfig4.pdf]

$n = 250$ ,  $p = 25$

**A**

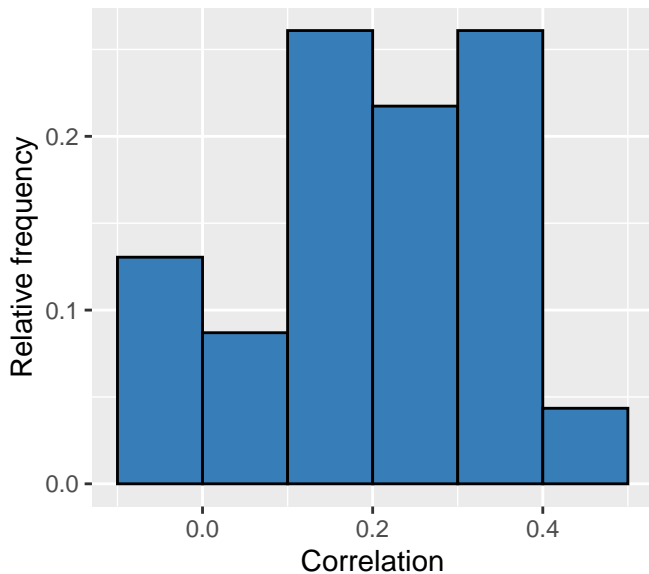

**B**

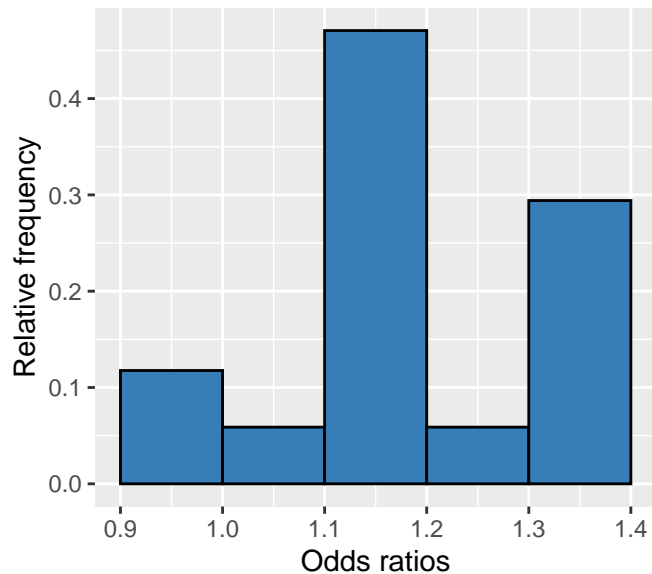

**C**

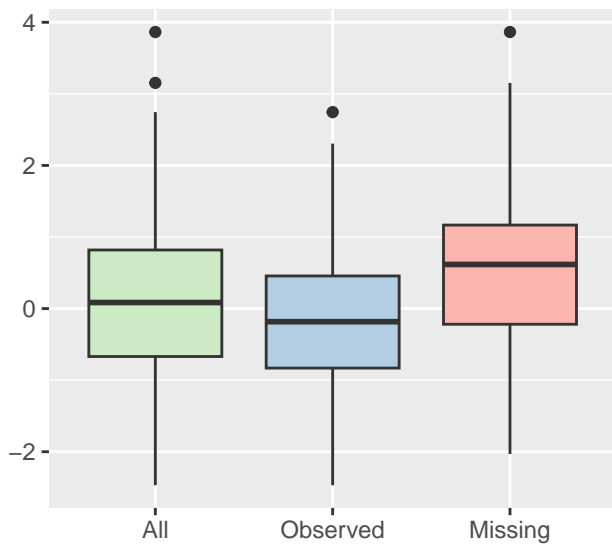

**D**

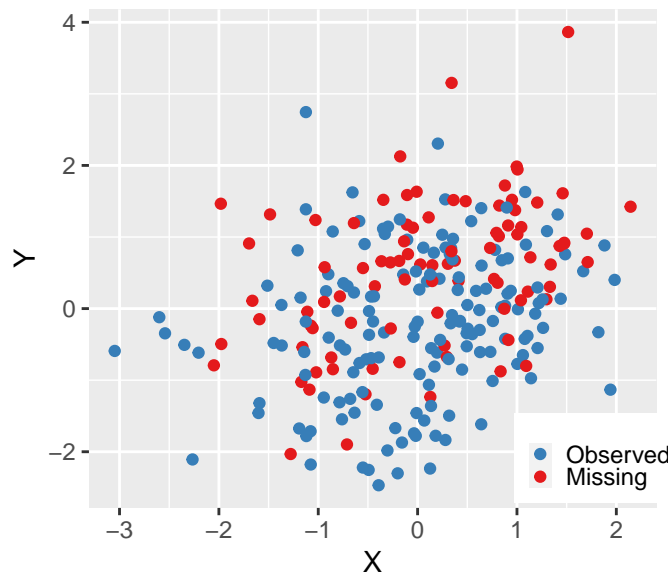

Supplement: Supporting Information 1 [file EMS194352-supplement-Supporting_Information_1.zip › code_resubmitted/sim_study_MWE/results/figures/suppfig6_n250_p25_pmy30_mod.pdf]

$n = 250$ ,  $p = 83$

**A**

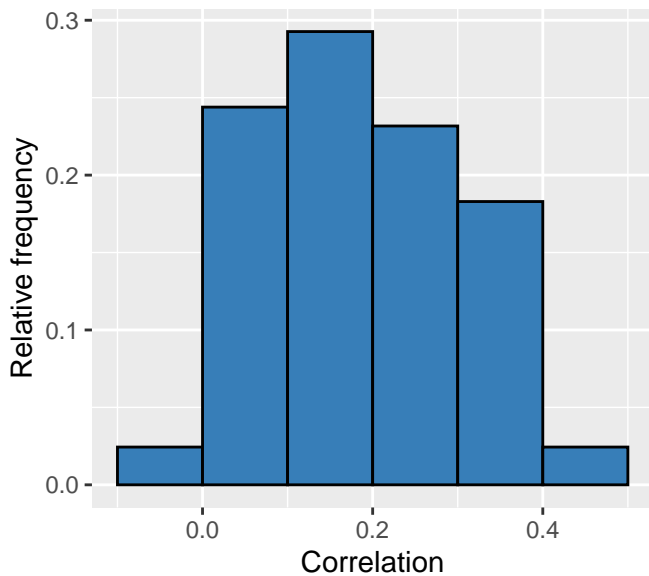

**B**

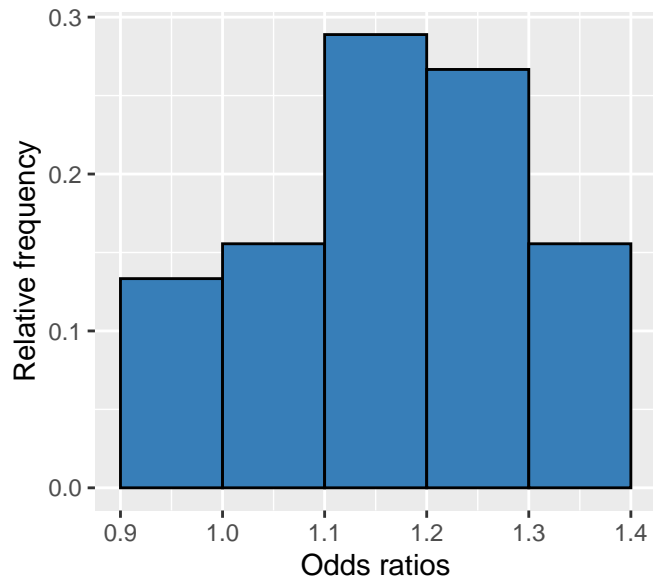

**C**

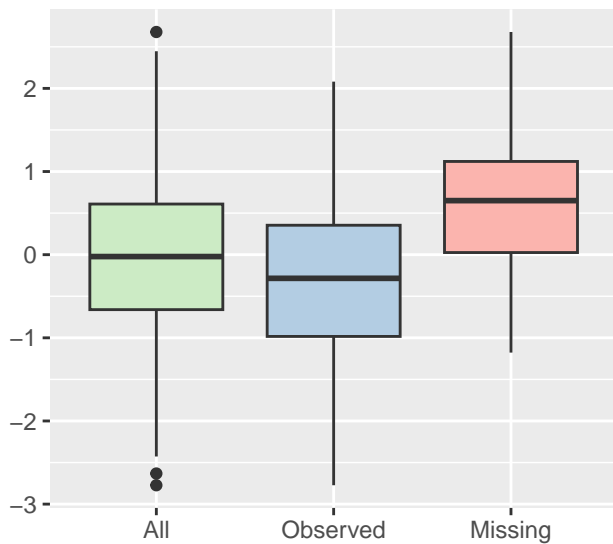

**D**

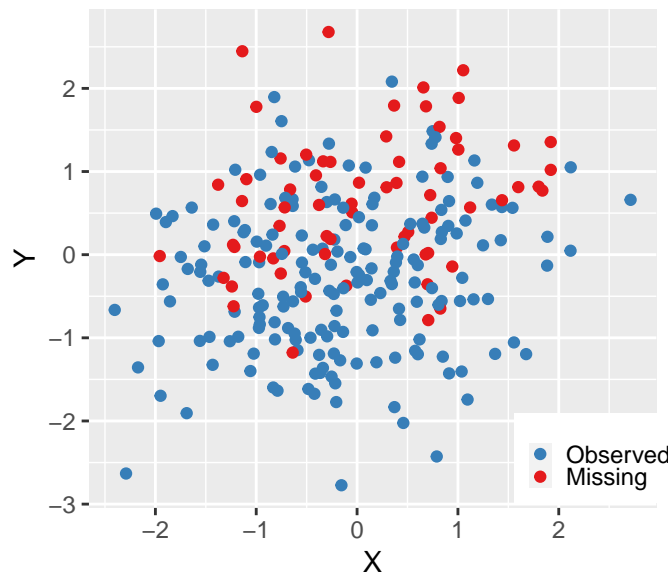

Supplement: Supporting Information 1 [file EMS194352-supplement-Supporting_Information_1.zip › code_resubmitted/sim_study_MWE/results/figures/suppfig7_n250_p83_pmy30_mod.pdf]

$n = 1000$ ,  $p = 100$

**A**

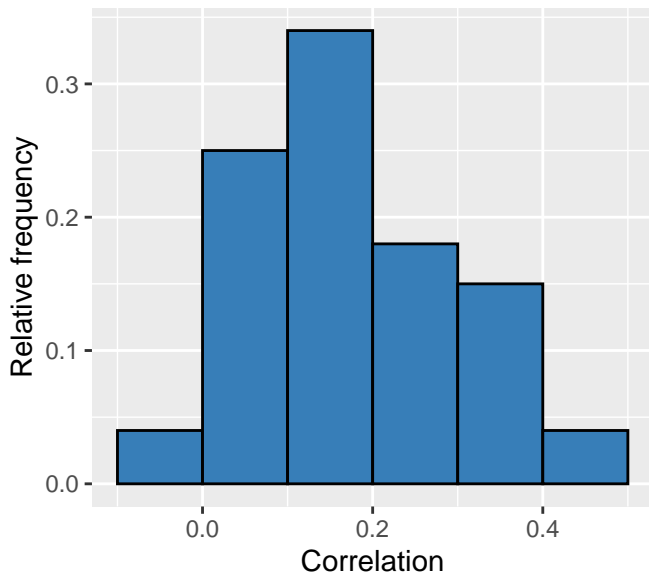

**B**

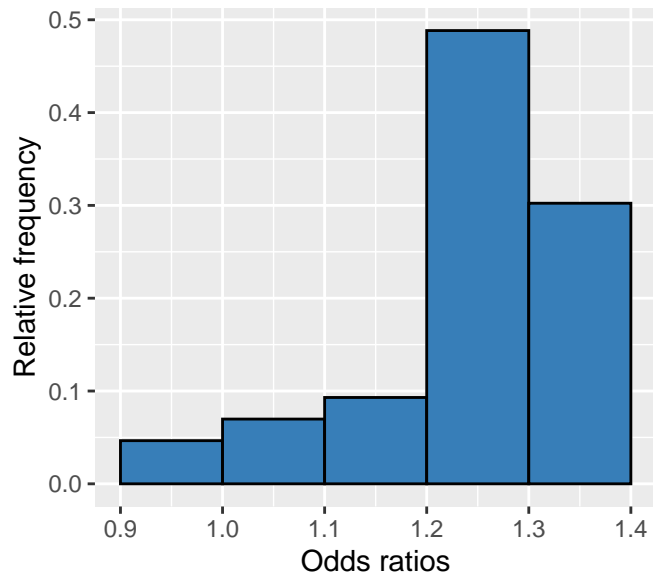

**C**

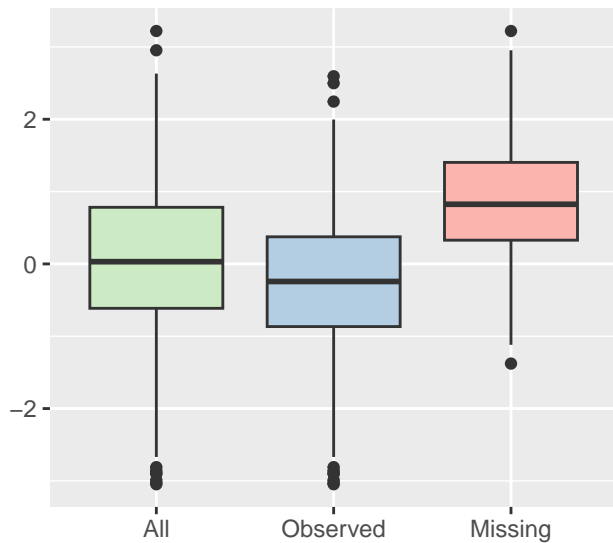

**D**

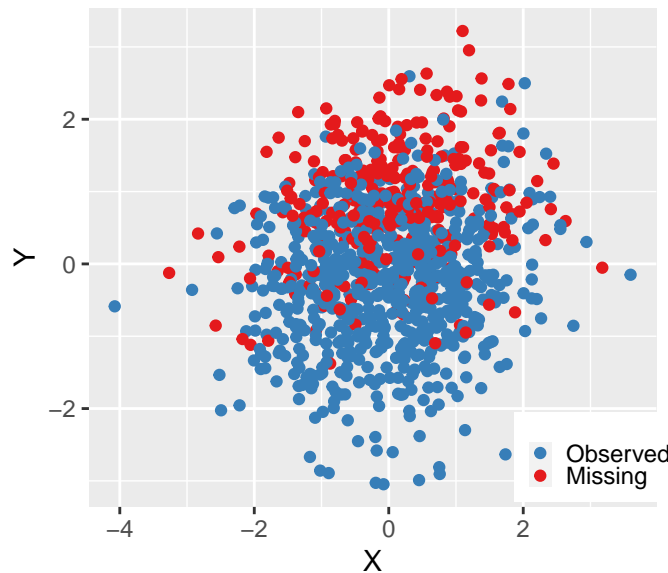

Supplement: Supporting Information 1 [file EMS194352-supplement-Supporting_Information_1.zip › code_resubmitted/sim_study_MWE/results/figures/suppfig8_n1000_p100_pmy30_mod.pdf]

n = 1000 , p = 333

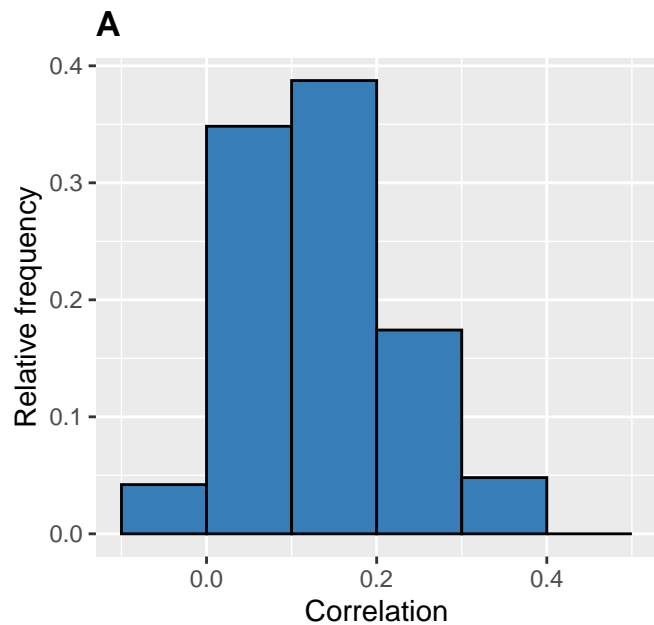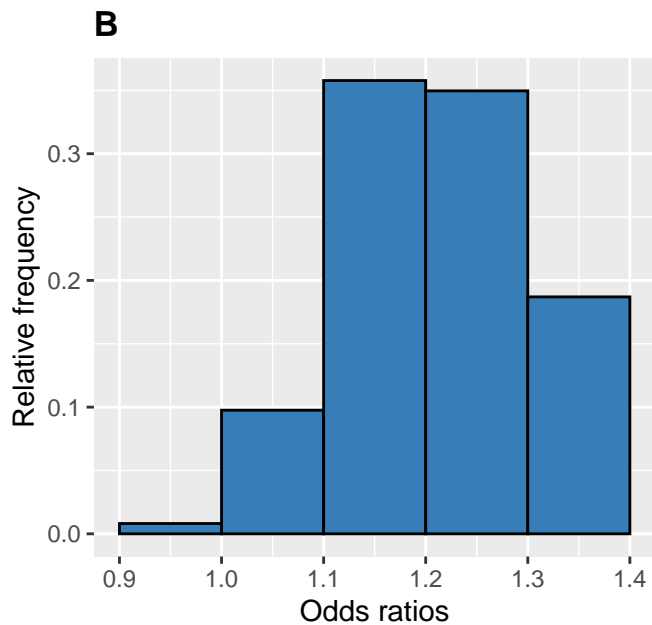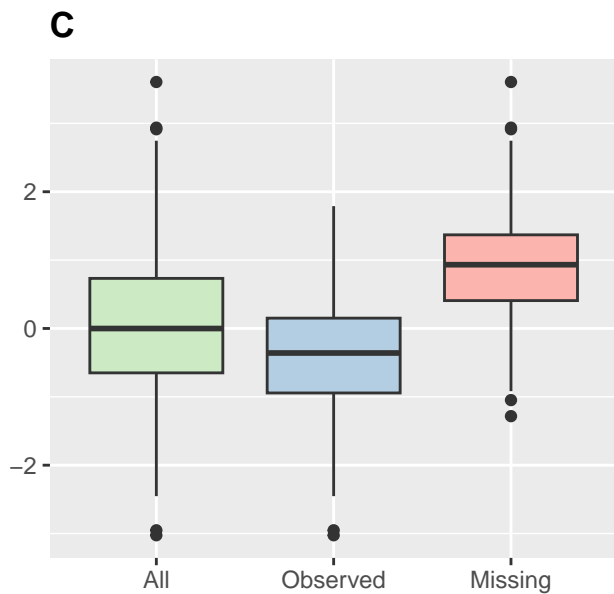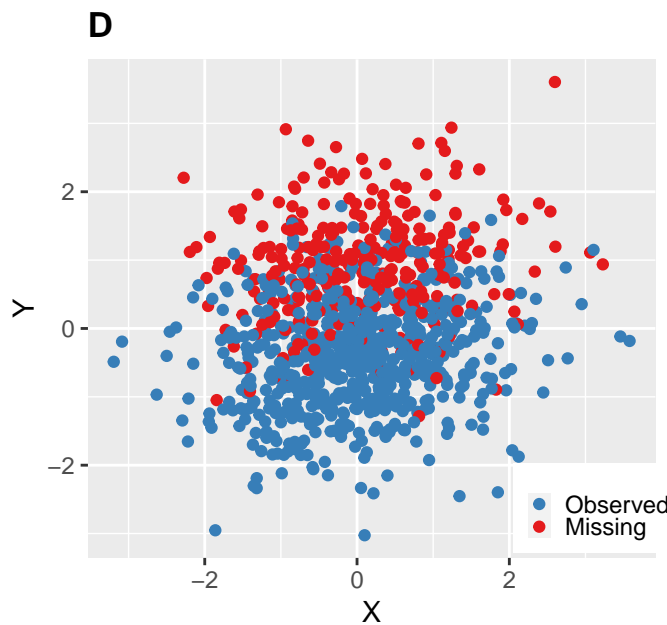

Supplement: Supporting Information 1 [file EMS194352-supplement-Supporting_Information_1.zip › code_resubmitted/sim_study_MWE/results/figures/suppfig9_n1000_p333_pmy30_mod.pdf]
