# Supplementary material for: A comparison of strategies for selecting auxiliary variables for multiple imputation[image]
Source: Biom J. Author manuscript; Available in PMC 2024 Mar 8. (PMC7615727; doi:10.1002/bimj.202200291)
Supplement: Supporting Information 2 [file EMS194352-supplement-Supporting_Information_2.pdf]

A comparison of strategies for selecting auxiliary variables for multiple imputation

SUPPORTING INFORMATION

Rheanna M. Mainzer[1,2*], Cattram D. Ngyuen[1,2], John B. Carlin[1,3], Margarita Moreno-Betancur[1,2], Ian R. White[4] and Katherine J. Lee[1,2]

[1]Clinical Epidemiology and Biostatistics Unit, Murdoch Children's Research Institute, Victoria, Australia

[2]Department of Paediatrics, The University of Melbourne, Victoria, Australia

[3]Centre for Epidemiology and Biostatistics, Melbourne School of Population and Global Health, The University of Melbourne, Victoria, Australia

[4]MRC Clinical Trials Unit, University College London, 90 High Holborn, London, UK

[*] Corresponding author: rheanna.mainzer@mcri.edu.au

Abbreviations: HRQoL, health-related quality of life; PedsQL: Paediatric Quality of Life Inventory; BMI, body mass index; BMIz, BMI z score; IndStat, indigenous status; NonEng, non-english speaking background; SES, socioeconomic status; GHM, Global Health Measure; SHCN, Special Health Care Needs; SDQ, Strengths and Difficulties questionnaire; MR, Matrix Reasoning test, PPVT: Peabody Picture Vocabulary test.

Contents

1 Description of variables in the LSAC case study **2**

2 Simulation study **4**

2.1 Correlation matrix of $(Y, X, Z, \boldsymbol{A})$ 4

2.2 Calculation of γ_0 6

2.3 Illustration of simulation scenarios 6

2.4 Additional details for the *Forward-FMI* strategy 14

2.5 Full results 16

2.6 Selected variables 19

1 Description of variables in the LSAC case study

Table 1 provides descriptions of all variables used in the LSAC case study and summarises the association with the HRQoL outcome variable. Figure 1 shows the distribution of the correlations between HRQoL and auxiliary variables. Figure 2 shows the distribution of odds ratios for missingness in HRQoL obtained from univariate logistic regressions of the missingness indicator for HRQoL on the auxiliary variables.

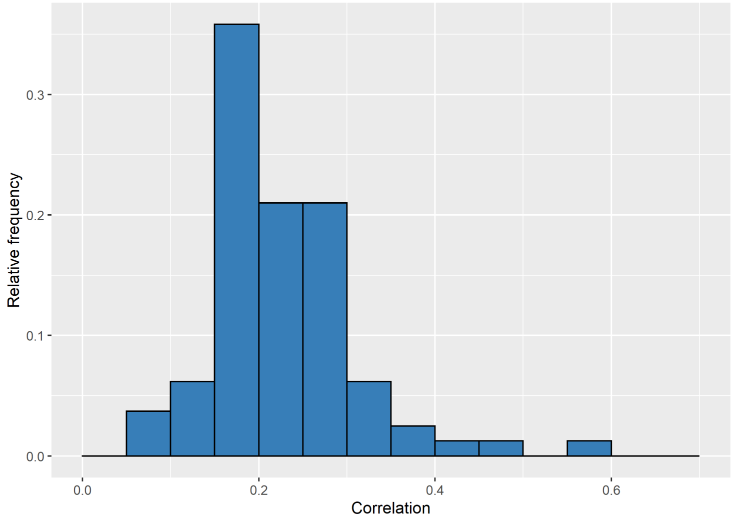

Figure 1: Histogram of the pairwise absolute Pearson correlation coefficients between auxiliary variables (continuous and ordinal) and HRQoL for the LSAC case study. Correlations are calculated for individuals with data on both variables.

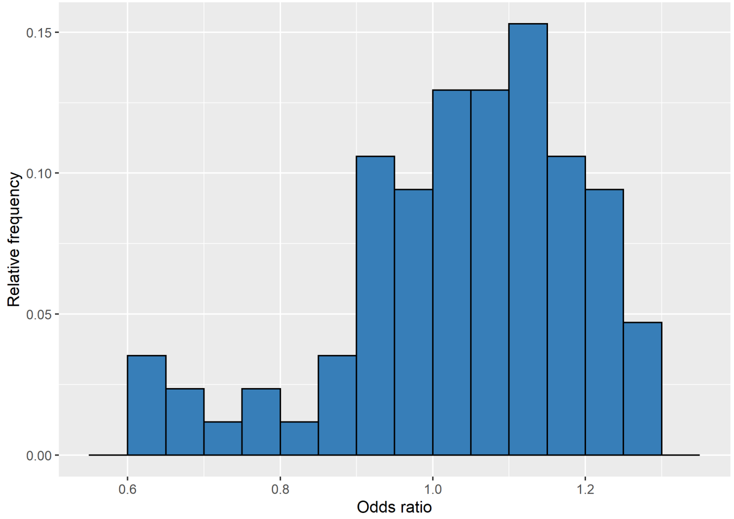

Figure 2: Histogram of estimated odds ratios of missingness in HRQoL, obtained from univariate logistic regressions of missingness indicator for HRQoL on auxiliary variables, for the LSAC case study.

Variable	Wave	Type	Details	Missing data, n (%)	Correlation with HRQoL	OR for missingness in HRQoL (95% CI)
HRQoL	4	Outcome	Continuous variable derived from PedsQL items; range 18.75 to 100; higher scores indicate better HRQoL	867 (17.4)	-	-
BMIz	1	Exposure	BMI, standardized by age and gender; range -4.6 to 4.9	49 (1)	-0.05	1 (0.93,1.08)
Female	-	Covariate	0 = male, 1 = female	0 (0)	0.05	1.03 (0.95,1.1)
Age	1	Covariate	Child age in months; range 48 to 67	0 (0)	-0.01	1.02 (0.95,1.1)
IndStat	-	Covariate	0 = non-indigenous, 1 = indigenous	2 (0)	-0.09	1.25 (1.18,1.32)
NonEng	-	Covariate	0 = english speaking background, 1 = non-english speaking background	37 (0.7)	-0.14	1.43 (1.35,1.53)
SES	1	Covariate	Standardized socioeconomic status of the child's family; range -3.7 to 3; higher scores indicate less disadvantage	18 (0.4)	0.16	0.55 (0.51,0.6)
PedsQL items	1	Auxiliary	5-point Likert scale response, ranges from 1 = "never a problem" to 5 = "always a problem"	789 (15.8), 979 (19.6)[1]	-0.28, -0.14[1]	0.8 (0.73,0.87), 1.19 (1.1,1.29)[1]
	2			1513 (30.4), 1602 (32.1)[1]	-0.33, -0.2[1]	0.85 (0.74,0.97), 1.2 (1.07,1.34)[1]
	3			1184 (23.8), 1284 (25.8)[1]	-0.37, -0.23[1]	0.89 (0.78,1.02), 1.29 (1.15,1.45)[1]
GHM	1	Auxiliary	Rating of child's current health, ranges from 1 = "Excellent" to 5 = "Poor"	1 (0)	-0.17	1.11 (1.03,1.19)
	2			519 (10.4)	-0.23	1.13 (1.03,1.23)
	3			652 (13.1)	-0.25	1.15 (1.03,1.27)
	4			823 (16.5)	-0.35	1.05 (0.79,1.38)
SHCN	1	Auxiliary	0 = child does not have a health condition that requires special care, 1 = child has a health condition that requires special care	49 (1)	-0.13	1.03 (0.96,1.11)
	2			668 (13.4)	-0.15	0.97 (0.88,1.07)
	3			652 (13.1)	-0.17	0.95 (0.84,1.07)
	4			826 (16.6)	-0.18	1.01 (0.76,1.33)
SDQ	1	Auxiliary	Scale score providing information on child behaviour; range 0 - 35; higher scores represent worse problems	15 (0.3)	-0.37	1.3 (1.21,1.39)
	2			646 (13)	-0.44	1.22 (1.12,1.34)
	3			1185 (23.8)	-0.49	1.24 (1.1,1.39)
	4			867 (17.4)	-0.59	0.65 (0.06,7.64)
MR	2	Auxiliary	Measure of child's non-verbal intelligence range 1 to 19; higher scores indicate better performance	570 (11.4)	0.11	0.79 (0.72,0.88)
	3			713 (14.3)	0.12	0.75 (0.67,0.84)
	4			880 (17.7)	0.16	0.6 (0.44,0.81)
PPVT	1	Auxiliary	Measure of child's vocabulary; range 28 - 106; higher scores indicate better performance	577 (11.6)	0.17	0.64 (0.59,0.7)
	2			666 (13.4)	0.13	0.7 (0.63,0.77)
	3			710 (14.2)	0.15	0.67 (0.59,0.75)

[1] min, max of the 21 (K cohort at wave 1) or 23 (K cohort at waves 2-4) items that make up the Generic Core Scales module of the PedsQL.

Table 1: Description of variables in the LSAC case study (n = 4983) and summary of associations with HRQoL. Correlations are pairwise Pearson correlation coefficients for the available cases. Odds ratios (ORs) are from univariate logistic regressions of the missingness indicator of HRQoL, where predictor variables are scaled to have mean 0 and variance 1. All analysis variables were completely observed for 81% of cases. All analysis and auxiliary variables were completely observed for 37% of cases.

2 Simulation study

2.1 Correlation matrix of $(Y, X, Z, \boldsymbol{A})$

The correlation matrices of $(Y, X, Z, \boldsymbol{A})$ used for data generation were designed to have the following desirable features:

1. A mix of realistic, marginal correlations between Y and the auxiliary variables.
2. Approximately 80% power to reject the null hypothesis that the coefficient of X, β_X, in the analysis model (see equation (2) in the manuscript) is equal to 0 at the 5% level of significance.
3. A similar structure across each different scenario.

This was achieved in two steps as follows. In the first step, we specified the desired correlations between all variables. Let ρ_{AB} denote the desired marginal correlation between A and B. We set $\rho_{XZ} = \rho_{YZ} = 0.3$ and calculated ρ_{XY} such that there was 80% power to reject the null hypothesis that $\beta_X = 0$ in the main analysis model at the 5% level of significance, with the power estimated by simulation. To allow for a variety of realistic correlations between Y and the auxiliary variables, we considered three groups of auxiliary variables, with each group consisting of approximately $p/3$ variables (any remaining variables due to p not being divisible by 3 were assigned to the third group). For the first group, the desired correlations between Y and auxiliary variables were set to random draws from the $N(0.4, 0.1^2)$; for the second group these correlations were set to random draws from the $N(0.2, 0.05^2)$ distribution; and for the third group these correlations were set to random draws from the $N(0.1, 0.05^2)$ distribution. Within each group, desired correlations between auxiliary variables had an exchangeable correlation structure, with off-diagonal elements equal to 0.4 for group 1, 0.2 for group 2 and 0.1 for group 3. All other correlations (e.g. ρ_{XA_1}) were set to 0. In the second step, we created a correlation matrix using the values above. We ensured that each matrix was positive definite using the procedure and R code provided by Hardin et al., adapted for our purposes, and by utilising functions in the `Matrix` package.[1] Note that this process of ensuring the correlation matrix was positive definite changed the values in the matrix. Therefore the power was recalculated and ρ_{XY} was updated iteratively until the value of ρ_{XY} which gave the desired 80% power was obtained. Figures 3 and 4 below illustrate the correlation matrix of $(Y, X, Z, \boldsymbol{A})$ for the scenarios where $p = 25$ and $p = 83$, respectively. All code is available at the first author's github page.

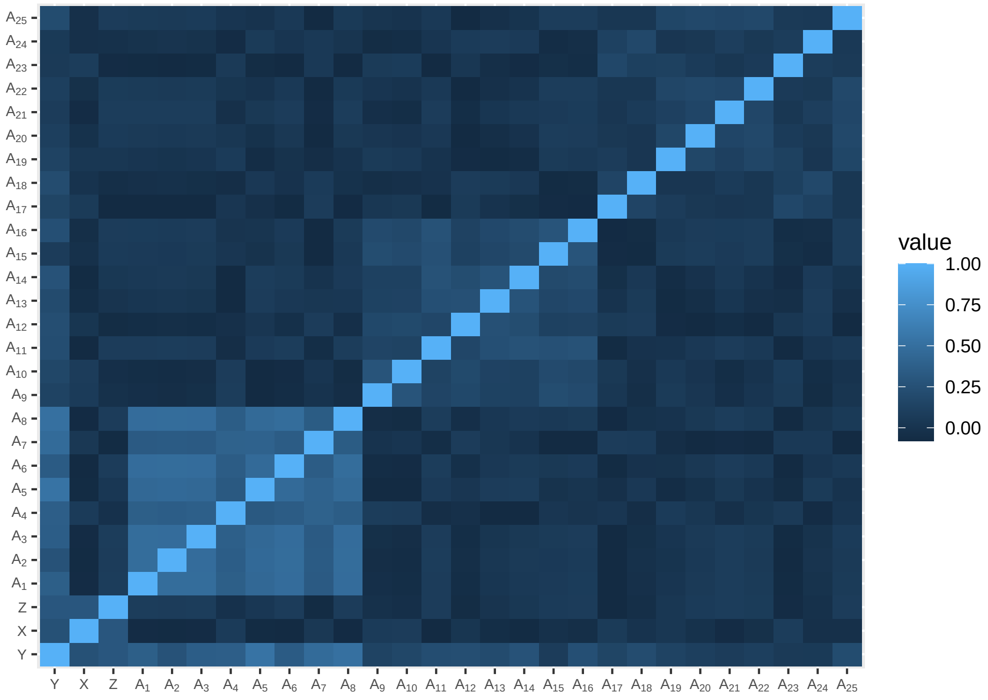

Figure 3: Correlation matrix of $(Y, X, Z, \boldsymbol{A})$ for the scenario where $p = 25$.

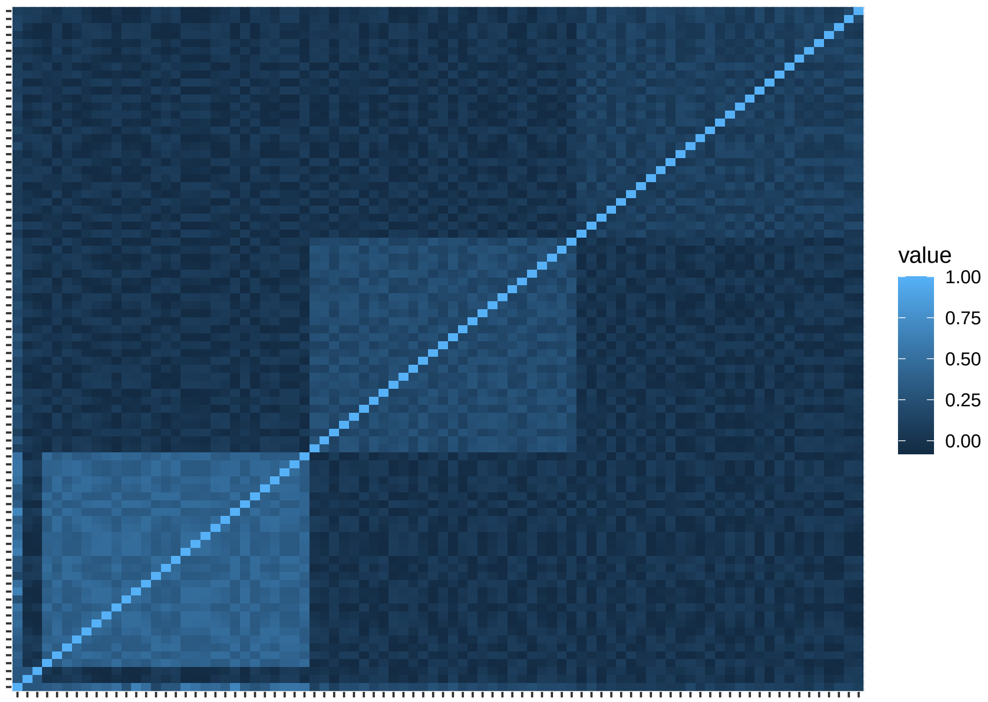

Figure 4: Correlation matrix of $(Y, X, Z, \boldsymbol{A})$ for the scenario where $p = 83$. Variable labels are omitted for readability, but follow the same order as Figure 3, i.e., $Y, X, Z, A_1, A_2, \ldots, A_{83}$ left to right on the x axis and bottom to top on the y axis.

2.2 Calculation of γ_0

Consider a grid of evenly spaced values $g = (g_1, \ldots, g_J)$ of length J. The value of γ_0 used in each simulation scenario was obtained as follows. First, we generated data for $(Y, X, Z, \boldsymbol{A})$ for $n = 10000$ individuals using the data generating process described in the paper. Next, for $j = 1, \ldots, J$, we set values of Y missing with a probability determined by the logistic regression model

$$\operatorname{logit} P(M_Y) = g_j + \gamma_X X + \gamma_Z Z + \boldsymbol{\gamma}_{\boldsymbol{A}}^{\top}, \boldsymbol{A},$$

where γ_X, γ_Z and $\boldsymbol{\gamma}_A{}^{\top}$ are specified for each scenario as described in the manuscript, and calculated the resulting proportion of missing values in Y. Let p_j denote the proportion of missing values in Y corresponding to g_j. To account for variability in the simulated data, we fit a linear regression model to the points $(g_1, p_1), \ldots, (g_J, p_J)$ which included a quadratic term for g. This quadratic term was included after investigation of the residuals of the fitted simple linear regression model. The value of γ_0 was obtained from the fitted model by setting the proportion of missing values equal to the desired proportion (0.3 or 0.5) and solving for g. This process is illustrated for each scenario in Figure 5.

2.3 Illustration of simulation scenarios

To visualise and check the simulated data, the following plots were created for a randomly selected data set for each scenario: a histogram of the correlations between Y and auxiliary variables, a histogram of odds ratios from univariate logistic regressions of M_Y on auxiliary variables, box plots of all (both observed and missing), observed and missing Y values, and scatter plots of X and Y, with points grouped by observed and missing values of Y. These plots are presented in Figures 6 to 11 for each of the simulation scenarios.

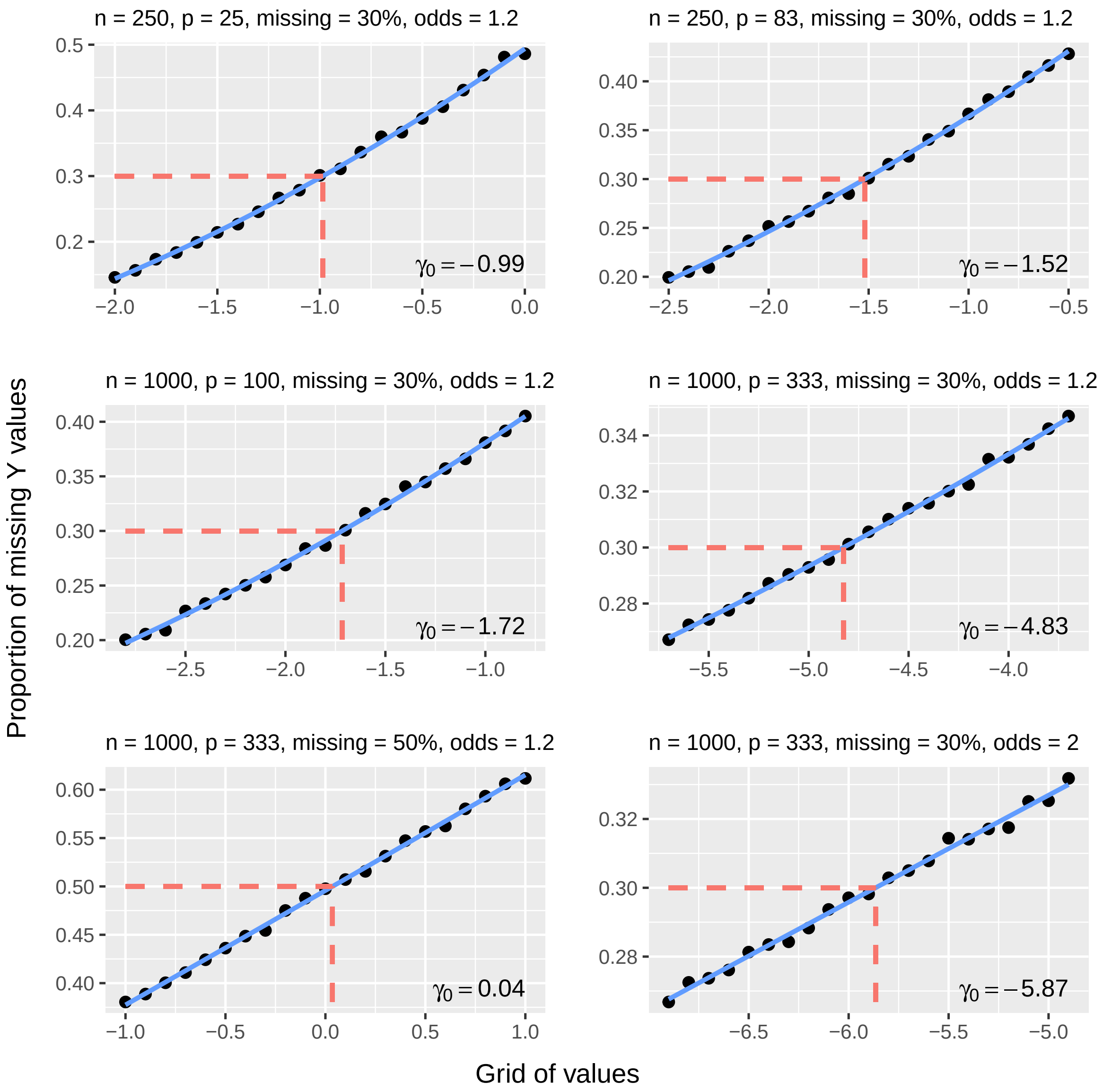

Figure 5: Illustration of the method used to calculate γ_0 for each of the simulation scenarios. The dashed horizontal line has y-intercept at the desired proportion of missing data (0.3 or 0.5) and the dashed vertical line has x-intercept at the value of γ_0.

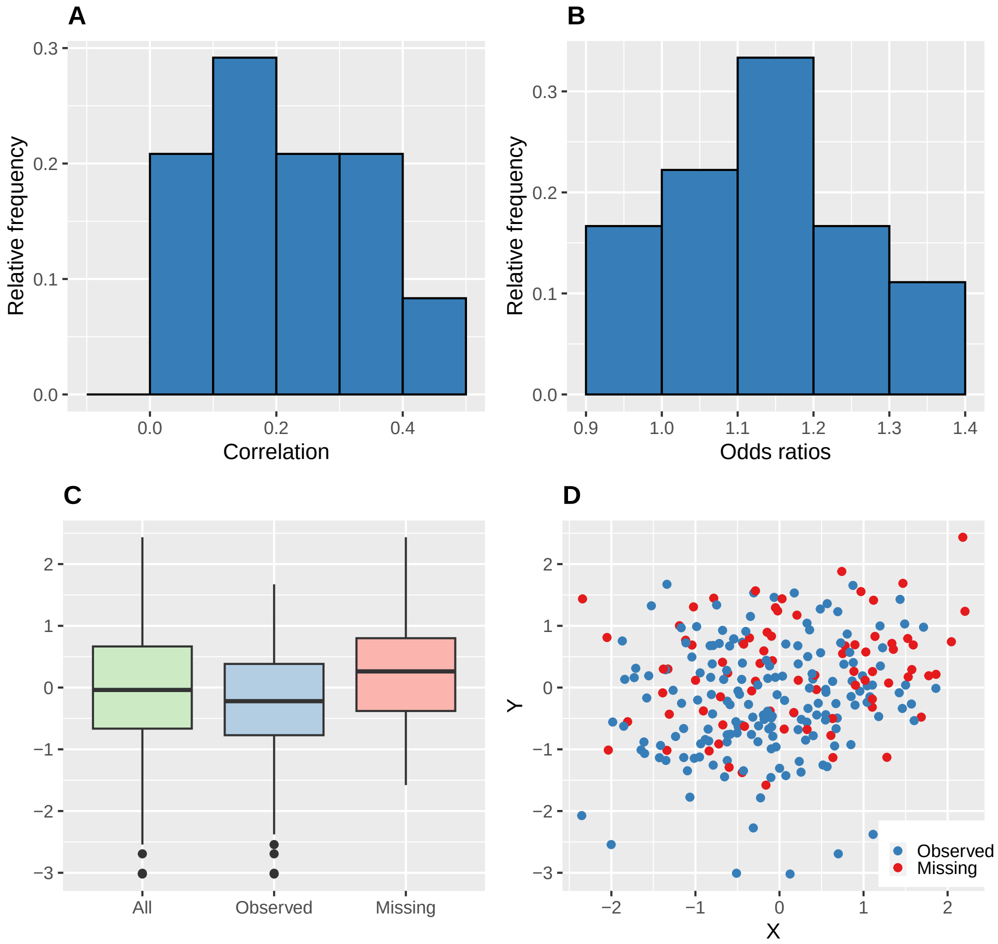

Figure 6: Histogram of correlations between Y and auxiliary variables (A), histogram of odds ratios from univariate logistic regressions of M_Y on auxiliary variables (B), box plots of all, observed and missing Y values (C), and scatter plots of X and Y including both observed and missing values of Y (D) for one simulated data set and for the scenario where $n = 250$ and $p = 25$.

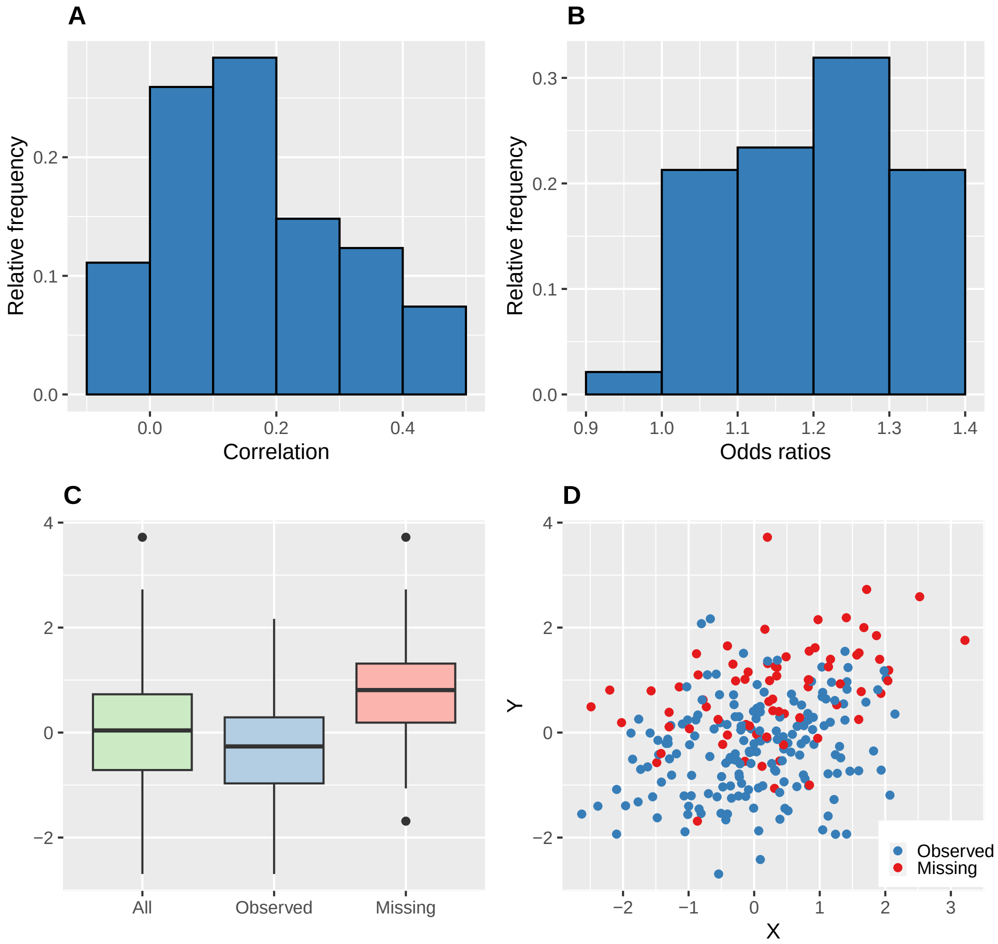

Figure 7: Histogram of correlations between Y and auxiliary variables (A), histogram of odds ratios from univariate logistic regressions of M_Y on auxiliary variables (B), box plots of all, observed and missing Y values (C), and scatter plots of X and Y including both observed and missing values of Y (D) for one simulated data set and for the scenario where $n = 250$ and $p = 83$.

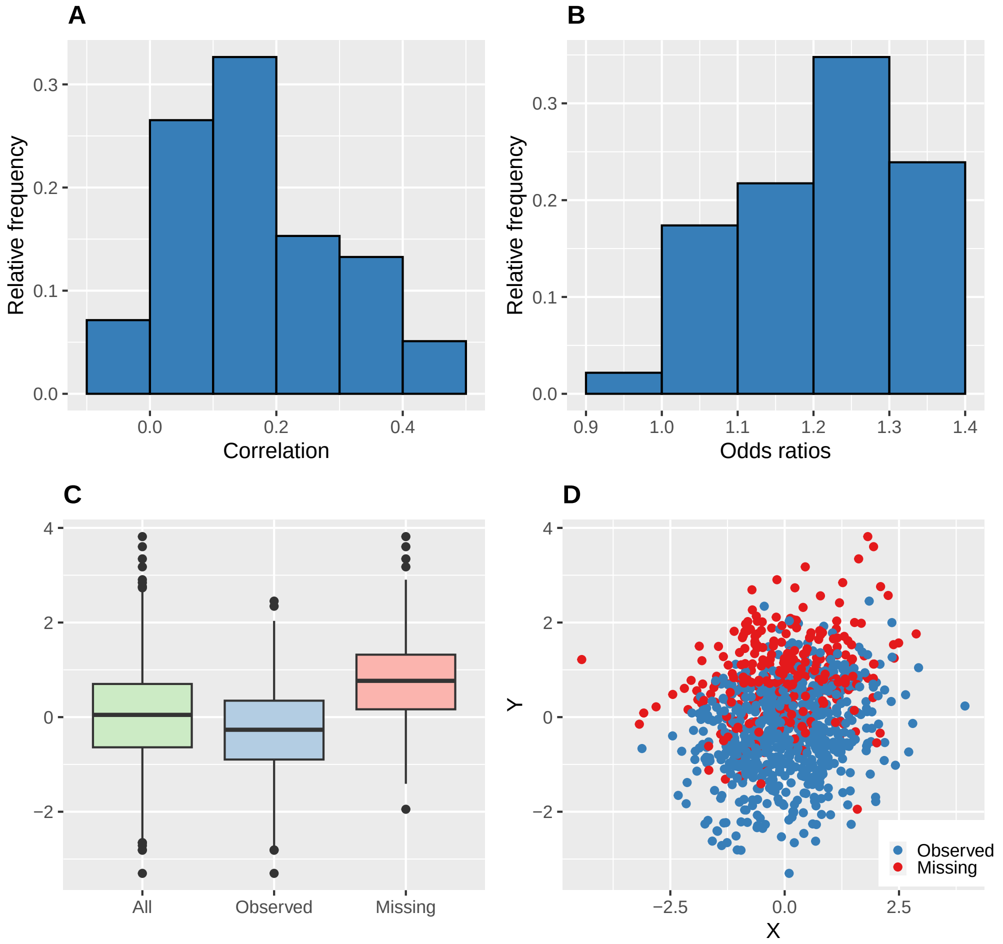

Figure 8: Histogram of correlations between Y and auxiliary variables (A), histogram of odds ratios from univariate logistic regressions of M_Y on auxiliary variables (B), box plots of all, observed and missing Y values (C), and scatter plots of X and Y including both observed and missing values of Y (D) for one simulated data set and for the scenario where $n = 1000$, $p = 100$, $P(M_Y = 1) = 0.3$ and the coefficients of X, Z and $\boldsymbol{A}$ in the missingness model are equal to $\log(1.2)$.

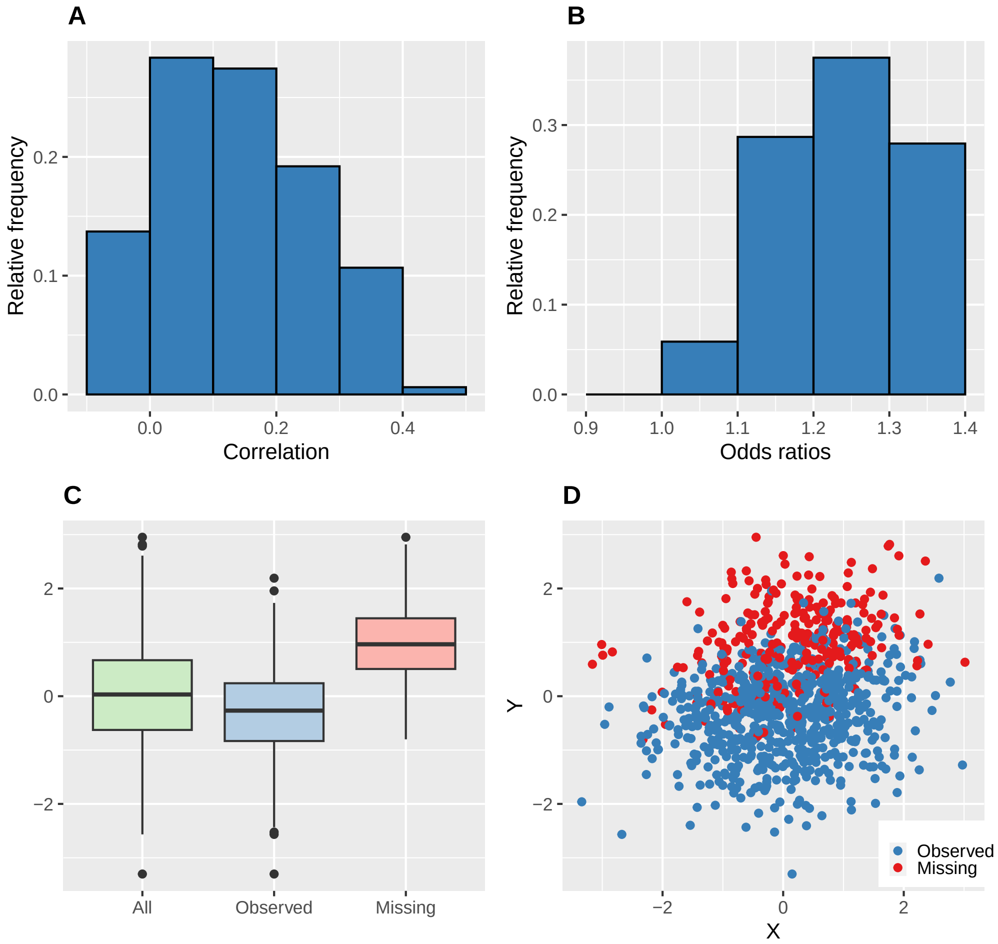

Figure 9: Histogram of correlations between Y and auxiliary variables (A), histogram of odds ratios from univariate logistic regressions of M_Y on auxiliary variables (B), box plots of all, observed and missing Y values (C), and scatter plots of X and Y including both observed and missing values of Y (D) for one simulated data set and for the scenario where $n = 1000$ and $p = 333$.

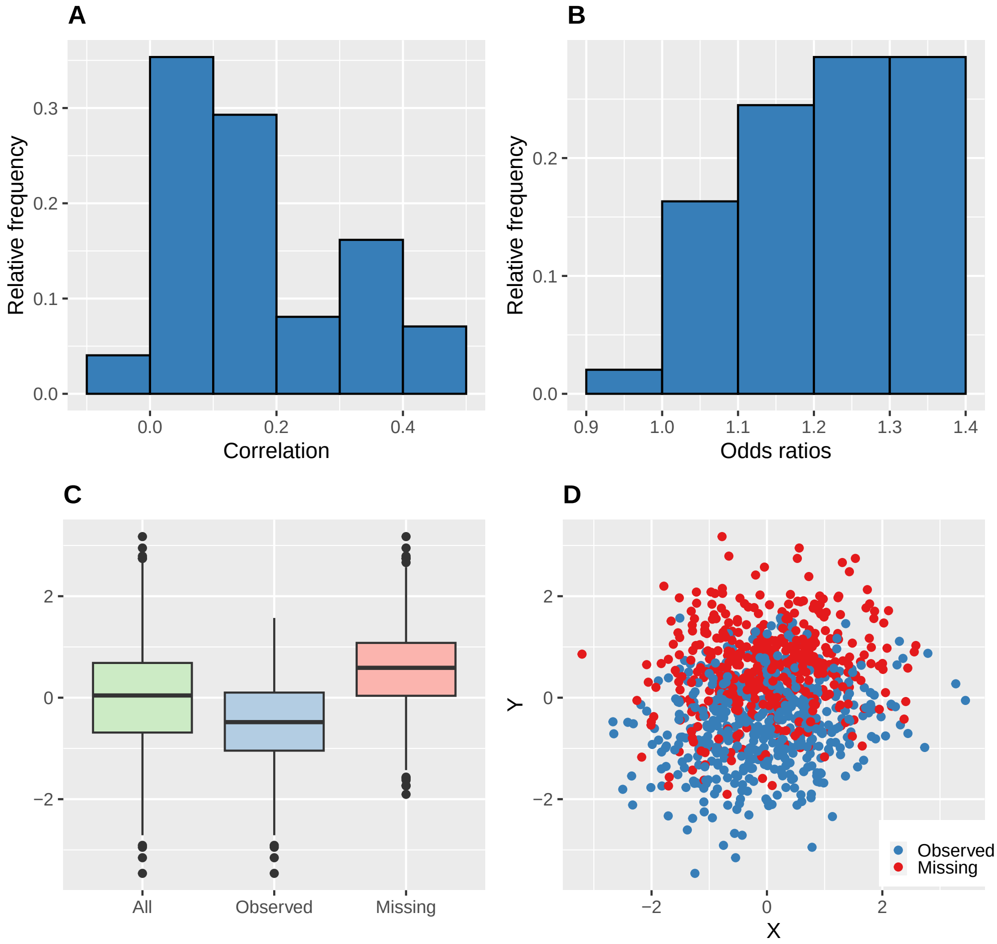

Figure 10: Histogram of correlations between Y and auxiliary variables (A), histogram of odds ratios from univariate logistic regressions of M_Y on auxiliary variables (B), box plots of all, observed and missing Y values (C), and scatter plots of X and Y including both observed and missing values of Y (D) for one simulated data set and for the scenario where $n = 1000$, $p = 100$, $Pr(M_Y = 1) = 0.5$ and the coefficients of X, Z and $\boldsymbol{A}$ in the missingness model are equal to $\log(1.2)$.

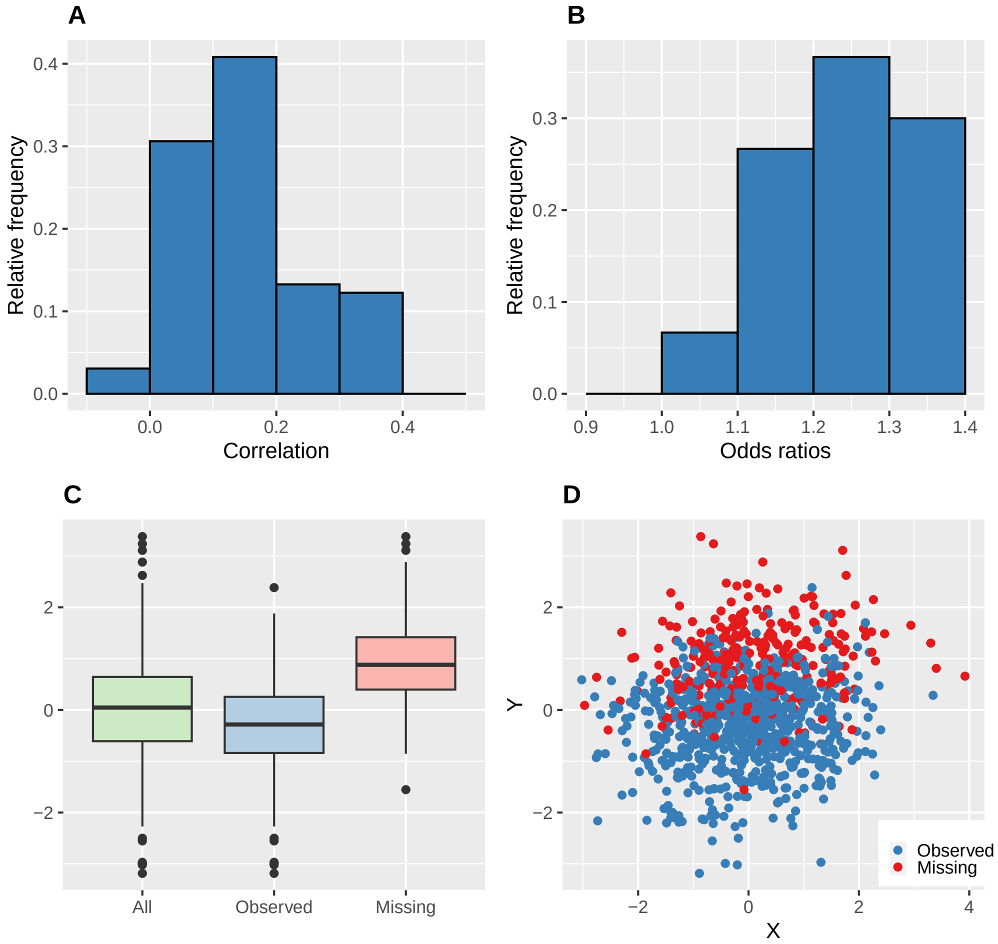

Figure 11: Histogram of correlations between Y and auxiliary variables (A), histogram of odds ratios from univariate logistic regressions of M_Y on auxiliary variables (B), box plots of all, observed and missing Y values (C), and scatter plots of X and Y including both observed and missing values of Y (D) for one simulated data set and for the scenario where $n = 1000$, $p = 100$, $Pr(M_Y = 1) = 0.3$ and the coefficients of X, Z and $\boldsymbol{A}$ in the missingness model are equal to $\log(2)$.

2.4 Additional details for the *Forward-FMI* strategy

In this section we provide a brief description of the theory and assumptions behind the *Forward-FMI* strategy as implemented in our study, and a justification for the implemented stopping rule.

Theory and assumptions

Let A^* denote a "proxy variable" which is created as the predicted values from a linear regression of Y on a subset of $\boldsymbol{A}$. Assume that M_Y follows a Bernoulli distribution and that the distribution of (Y, A^*), given M_Y, is bivariate normal with distinct means and variances for respondents ($M_Y = 0$) and nonrespondents ($M_Y = 1$). Also assume that the probability that Y is missing depends only on A^*. Under these assumptions, the maximum likelihood estimates of μ_Y and the FMI can be expressed as functions of the observed data. For further details and extensions under different assumptions we refer the reader to the work of [2] and [3].

Stopping rule

Andridge and Thompson suggest two approaches for selecting auxiliary variables based on the *Forward-FMI* strategy.[2] The first approach is by examining graphs of the FMI and identifying the point at which changes in the FMI level off. The second approach is to add additional auxiliary variables if they significantly decrease the FMI, which could be implemented via the bootstrap (or a similar) procedure to estimate the difference in FMI between candidate imputation models. However, neither of these approaches was feasible for the current simulation study. Therefore, we devised a simple data-based stopping rule for the *Forward-FMI* strategy. That is, the forward selection algorithm ended when the absolute change in the estimated FMI (between steps of the algorithm) was less than a pre-specified percentage of the percentage of missing data in Y. For the scenarios where $p = 25$, $p = 83$ and $p = 100$, the algorithm stopped when the absolute change in the estimated FMI was less than 1% of the proportion of missing data in Y. For the scenario where $p = 333$, the algorithm stopped when the absolute change in the estimated FMI was less than 0.5% of the proportion of missing data in Y. Figure 12 presents graphs of the absolute change in estimated FMI as a function of the number of auxiliary variables included in the imputation model for a randomly selected data set and each of the simulation scenarios. This figure illustrates that the number of auxiliary variables chosen approximates the point at which the FMI begins to level off, consistent with the approach used by Andridge and Thompson[2]. In other words, the devised data-based stopping rule was appropriate for use in this simulation study.

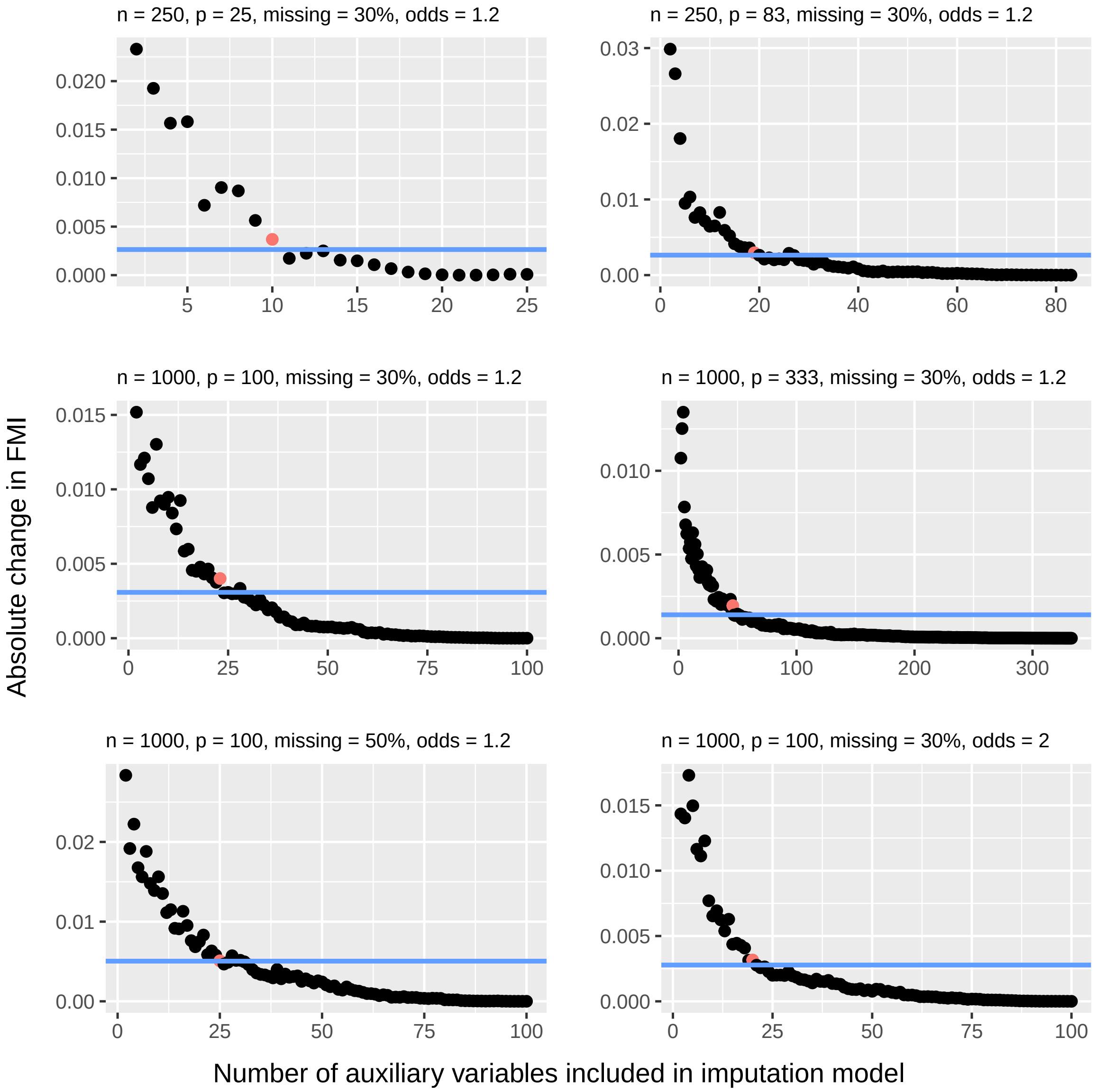

Figure 12: Scatterplots of the absolute change in estimated FMI at each step of the forward selection algorithm against the number of auxiliary variables included in the imputation model for one randomly selected simulated data set and each simulation scenario. The horizontal blue line indicates the value used in the stopping rule of the forward selection algorithm. The x-coordinate of the red point indicates the number of auxiliary variables included in the imputation model.

2.5 Full results

Strategy	β_X							μ_Y					
	**Bias**	**EmpSE**	**RelBias**	**StdBias**	**ModSE**	**RelSE**	**Cov**	**Bias**	**EmpSE**	**StdBias**	**ModSE**	**RelSE**	**Cov**
$n = 250$, $p = 25$, missing = 30%, odds = 1.2													
CCA	-0.008	0.074	-4.8	-11.3	0.074	-0.0	95	-0.162	0.074	-219.2	0.074	-0.3	41
Full	0.003	0.069	1.9	4.7	0.070	1.2	96	-0.002	0.071	-3.3	0.071	0.3	95
Quickpred-pt2	-0.001	0.069	-0.5	-1.4	0.069	1.2	96	-0.025	0.071	-35.8	0.070	-1.1	93
Quickpred-pt4	-0.007	0.072	-3.8	-9.2	0.072	-0.1	95	-0.094	0.077	-122.0	0.071	-7.3	72
PredMiss	0.003	0.072	1.5	3.7	0.072	0.1	96	-0.010	0.072	-14.4	0.073	1.2	95
PcAux	0.003	0.070	1.5	3.8	0.069	-0.2	95	-0.010	0.070	-14.8	0.070	-0.3	95
Forward	-0.004	0.068	-2.3	-5.8	0.069	1.3	95	-0.049	0.071	-69.0	0.069	-2.8	88
Forward-sw	-0.004	0.069	-2.5	-6.5	0.069	0.4	96	-0.049	0.071	-68.8	0.069	-3.0	88
Forward-FMI	-0.004	0.068	-2.2	-5.5	0.069	1.2	96	-0.028	0.071	-39.9	0.069	-2.7	91
LASSO	0.002	0.069	1.4	3.5	0.069	0.6	96	-0.004	0.070	-5.5	0.070	-1.1	94
$n = 250$, $p = 83$, missing = 30%, odds = 1.2													
CCA	-0.014	0.072	-8.1	-19.0	0.070	-1.7	94	-0.288	0.069	-418.4	0.069	0.2	1
Full	-0.001	0.065	-0.5	-1.4	0.064	-1.4	95	0.003	0.064	4.4	0.063	-0.6	95
Quickpred-pt2	-0.005	0.065	-2.9	-7.5	0.065	-0.8	95	-0.038	0.065	-58.0	0.065	-0.2	91
Quickpred-pt4	-0.007	0.068	-3.9	-9.7	0.067	-1.2	95	-0.109	0.067	-162.1	0.067	-0.2	63
PredMiss	-0.000	0.068	-0.1	-0.4	0.067	-0.4	95	0.013	0.067	19.4	0.067	1.1	95
PcAux	0.001	0.071	0.4	1.0	0.070	-1.4	95	0.029	0.072	40.9	0.071	-0.2	94
Forward	-0.012	0.064	-6.9	-18.2	0.064	0.2	95	-0.091	0.065	-140.7	0.064	-1.9	69
Forward-sw	-0.011	0.064	-6.6	-17.4	0.064	-0.2	95	-0.093	0.065	-142.4	0.064	-2.1	69
Forward-FMI	-0.014	0.066	-8.1	-20.7	0.065	-0.9	94	-0.038	0.066	-57.4	0.064	-2.9	90
LASSO	-0.001	0.065	-0.6	-1.5	0.064	-1.4	95	0.003	0.064	4.4	0.063	-0.6	95

Table 2: Bias, empirical standard error (EmpSE), relative bias (RelBias), standardised bias (StdBias), average model standard error (ModSE), relative error in the model standard error (RelSE) and 95% confidence interval coverage (Cov) for β_X and μ_Y, for each analysis strategy and the simulation scenarios where $n = 250$, $p = 25$, missing = 30%, odds = 1.2 and $n = 250$, $p = 83$, missing = 30%, odds = 1.2. MI estimates were successfully obtained for all simulated datasets (convergence = 1 for all strategies and scenarios).

	β_X							μ_Y					
**Strategy**	**Bias**	**EmpSE**	**RelBias**	**StdBias**	**ModSE**	**RelSE**	**Cov**	**Bias**	**EmpSE**	**StdBias**	**ModSE**	**RelSE**	**Cov**
$n = 1000$, $p = 100$, missing = 30%, odds = 1.2													
CCA	-0.011	0.033	-13.0	-34.4	0.034	1.9	94	-0.333	0.033	-1012.0	0.033	1.3	0
Full	0.000	0.032	0.2	0.4	0.032	1.5	96	0.001	0.033	2.6	0.032	-2.7	94
Quickpred-pt2	-0.003	0.031	-3.9	-11.1	0.032	3.3	96	-0.089	0.035	-253.0	0.032	-8.6	24
Quickpred-pt4	-0.006	0.032	-6.3	-17.3	0.033	2.1	95	-0.153	0.036	-425.7	0.033	-8.9	1
PredMiss	0.000	0.032	0.3	0.7	0.033	1.8	96	0.003	0.033	8.1	0.032	-2.1	94
PcAux	-0.000	0.034	-0.2	-0.5	0.035	0.4	95	0.002	0.036	5.9	0.035	-1.5	94
Forward	-0.004	0.031	-4.2	-12.1	0.031	2.7	95	-0.074	0.032	-228.0	0.031	-3.2	35
Forward-sw	-0.004	0.031	-4.5	-12.9	0.031	2.4	96	-0.075	0.033	-230.3	0.031	-4.0	33
Forward-FMI	-0.004	0.032	-4.2	-11.6	0.032	1.3	95	-0.052	0.034	-156.2	0.032	-5.2	61
LASSO	0.000	0.032	0.2	0.4	0.032	1.5	96	0.001	0.033	3.4	0.032	-2.7	94
$n = 1000$, $p = 333$, missing = 30%, odds = 1.2													
CCA	-0.000	0.033	-0.3	-0.9	0.032	-1.2	95	-0.385	0.031	-1242.2	0.031	1.0	0
Full	0.000	0.033	0.2	0.6	0.033	-0.4	96	0.001	0.031	2.0	0.032	0.8	95
Quickpred-pt2	-0.000	0.032	-0.5	-1.3	0.032	0.4	95	-0.083	0.033	-254.4	0.033	0.1	29
Quickpred-pt4	-0.001	0.032	-1.1	-3.0	0.032	-0.5	95	-0.253	0.054	-464.8	0.032	-40.6	0
PredMiss	0.000	0.033	0.3	0.7	0.033	-0.0	95	0.003	0.032	8.4	0.032	1.2	95
PcAux	0.001	0.035	0.8	2.1	0.034	-0.9	95	-0.007	0.035	-20.1	0.035	1.1	95
Forward	-0.004	0.031	-4.0	-11.8	0.031	0.0	95	-0.110	0.032	-347.7	0.031	-0.2	7
Forward-sw	-0.003	0.031	-3.7	-10.8	0.031	-0.6	95	-0.112	0.031	-357.4	0.031	0.0	5
Forward-FMI	-0.004	0.033	-4.8	-13.2	0.032	-1.3	95	-0.047	0.033	-144.8	0.032	-1.5	69
LASSO	0.000	0.033	0.2	0.5	0.033	-0.4	95	0.001	0.031	1.7	0.032	0.8	95

Table 3: Bias, empirical standard error (EmpSE), relative bias (RelBias), standardised bias (StdBias), average model standard error (ModSE), relative error in the model standard error (RelSE) and 95% confidence interval coverage (Cov) for β_X and μ_Y, for each analysis strategy and the simulation scenarios where $n = 1000$, $p = 333$, missing = 30%, odds = 1.2 and $n = 1000$, $p = 100$, missing = 30%, odds = 1.2. MI estimates were successfully obtained for all simulated datasets (convergence = 1 for all strategies and scenarios).

	β_X							μ_Y					
**Strategy**	**Bias**	**EmpSE**	**RelBias**	**StdBias**	**ModSE**	**RelSE**	**Cov**	**Bias**	**EmpSE**	**StdBias**	**ModSE**	**RelSE**	**Cov**
$n = 1000$, $p = 100$, missing = 50%, odds = 1.2													
CCA	-0.015	0.032	-17.5	-47.8	0.032	-0.0	92	-0.378	0.031	-1213.4	0.032	1.5	0
Full	0.000	0.033	0.1	0.1	0.032	-0.9	95	0.000	0.031	0.4	0.032	1.9	95
Quickpred-pt2	-0.004	0.031	-4.7	-13.2	0.031	-0.5	95	-0.100	0.033	-303.5	0.032	-3.4	13
Quickpred-pt4	-0.009	0.032	-9.8	-27.3	0.031	-0.8	94	-0.204	0.035	-586.0	0.032	-8.7	0
PredMiss	0.000	0.033	0.2	0.5	0.032	-0.8	95	0.002	0.032	5.1	0.032	1.5	96
PcAux	0.000	0.035	0.3	0.8	0.034	-0.7	95	0.002	0.037	6.2	0.036	-1.5	95
Forward	-0.005	0.031	-5.8	-16.6	0.031	-1.1	94	-0.098	0.031	-313.5	0.031	-2.1	12
Forward-sw	-0.005	0.031	-6.0	-17.2	0.031	-0.9	95	-0.099	0.031	-315.5	0.031	-2.0	11
Forward-FMI	-0.005	0.032	-5.2	-14.5	0.031	-1.3	95	-0.068	0.033	-208.9	0.031	-4.0	41
LASSO	0.000	0.033	0.1	0.2	0.032	-0.9	95	0.000	0.031	1.5	0.032	1.8	95
$n = 1000$, $p = 100$, missing = 30%, odds = 2													
CCA	-0.015	0.038	-17.1	-39.3	0.039	1.0	93	-0.535	0.039	-1379.4	0.038	-1.9	0
Full	0.001	0.032	0.7	2.0	0.032	0.5	95	-0.000	0.032	-0.8	0.032	-1.3	95
Quickpred-pt2	-0.004	0.033	-4.5	-12.2	0.033	0.9	95	-0.151	0.041	-367.0	0.035	-14.4	2
Quickpred-pt4	-0.008	0.035	-9.3	-23.6	0.036	2.2	95	-0.278	0.049	-562.5	0.037	-24.8	0
PredMiss	0.001	0.032	1.0	2.7	0.033	0.9	95	0.002	0.033	5.6	0.033	-1.6	94
PcAux	0.000	0.037	0.3	0.6	0.037	0.3	95	0.001	0.042	1.9	0.042	0.4	95
Forward	-0.006	0.032	-7.3	-19.9	0.033	1.4	95	-0.181	0.041	-441.6	0.035	-15.4	0
Forward-sw	-0.007	0.032	-7.6	-20.8	0.033	1.9	95	-0.183	0.041	-448.4	0.035	-15.1	0
Forward-FMI	-0.006	0.032	-6.9	-18.8	0.033	0.5	94	-0.088	0.039	-223.8	0.033	-15.2	28
LASSO	0.001	0.032	0.7	2.0	0.032	0.5	95	0.000	0.032	0.6	0.032	-1.3	95

Table 4: Bias, empirical standard error (EmpSE), relative bias (RelBias), standardised bias (StdBias), average model standard error (ModSE), relative error in the model standard error (RelSE) and 95% confidence interval coverage (Cov) for β_X and μ_Y, for each analysis strategy and the simulation scenarios where $n = 1000$, $p = 100$, missing = 50%, odds = 1.2 and $n = 1000$, $p = 100$, missing = 30%, odds = 2. MI estimates were successfully obtained for all simulated datasets (convergence = 1 for all strategies and scenarios).

2.6 Selected variables

The following figures present the average number of auxiliary variables selected across simulation runs, expressed as a proportion of the number of auxiliary variables and stratified by auxiliary variable group. These figures are presented for each simulation scenario and for the relevant analysis strategies. The groups are determined by the true correlation between Y and auxiliary variables used for data generation (categorised as between 0 and 0.1, 0.1 and 0.2, 0.2 and 0.4, and greater than 0.4) and the coefficients of the auxiliary variables in the missingness model ($\gamma \neq 0$ and $\gamma = 0$ are used in the figures to denote auxiliary variables that do, and do not, respectively, appear in the missingness model). The number of variables in each group is also given.

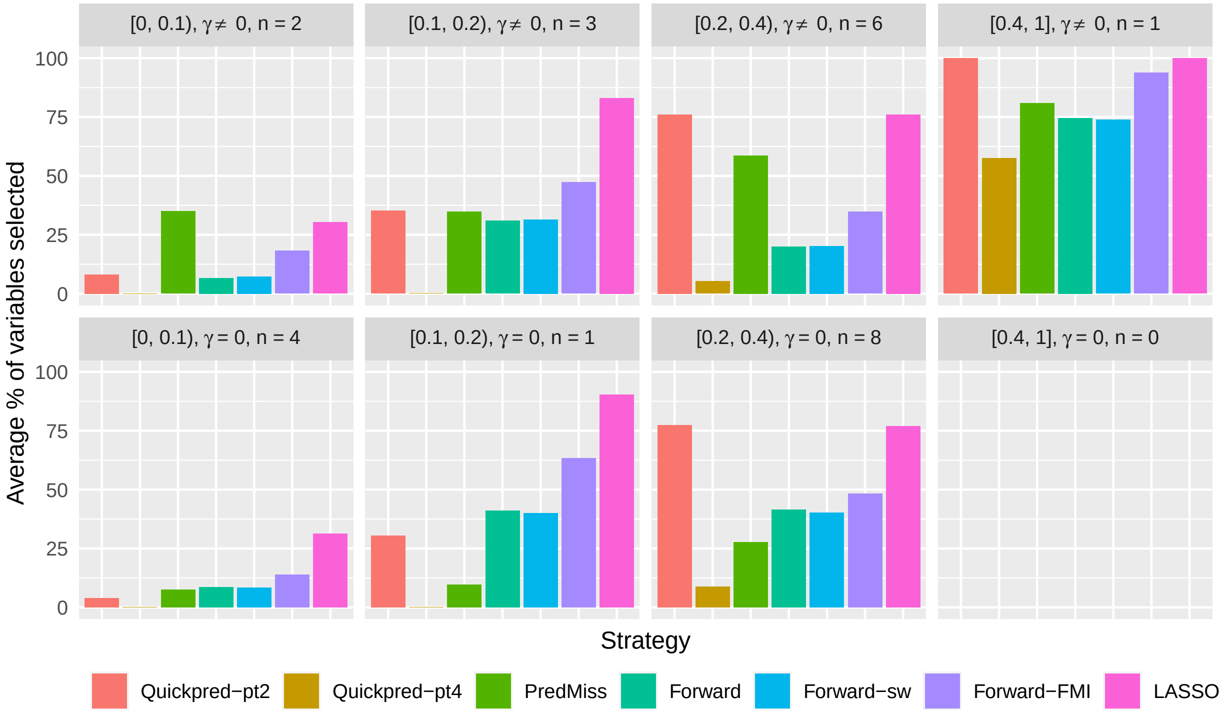

Figure 13: Average number of auxiliary variables selected across simulation runs, expressed as a proportion of the number of auxiliary variables, stratified by auxiliary variable group, for the scenario where $n = 250$ and $p = 25$.

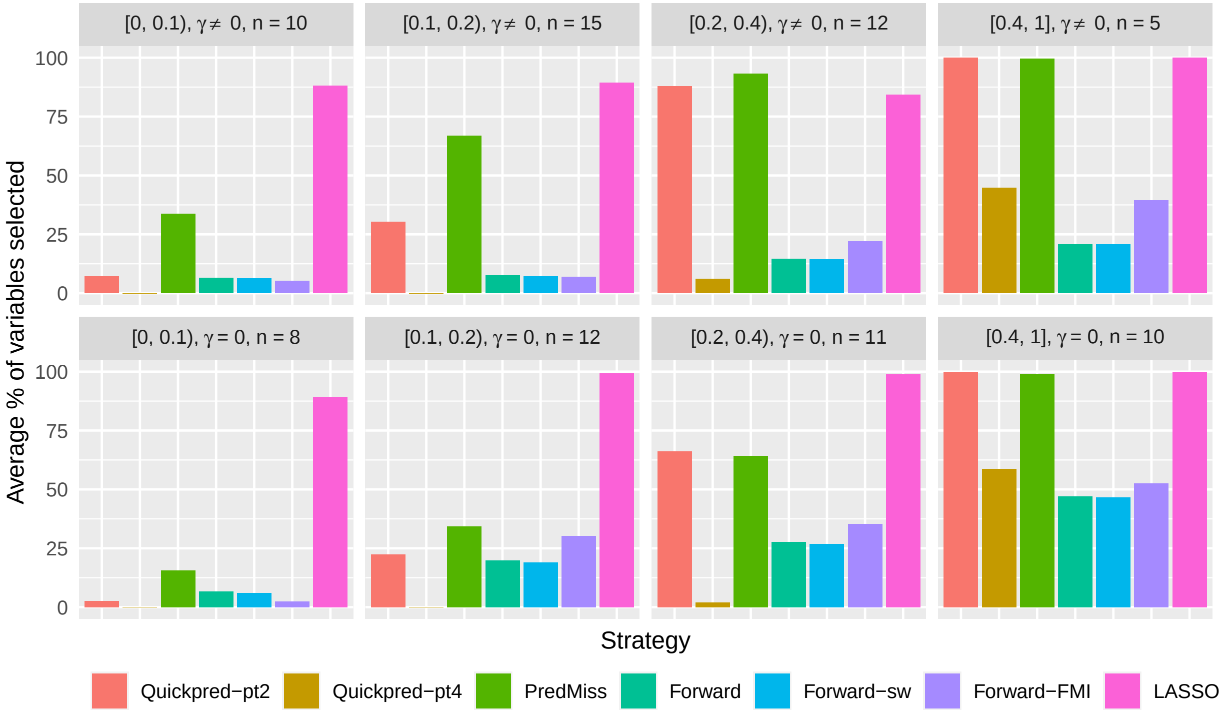

Figure 14: Average number of auxiliary variables selected across simulation runs, expressed as a proportion of the number of auxiliary variables, stratified by auxiliary variable group, for the scenario where $n = 250$ and $p = 83$.

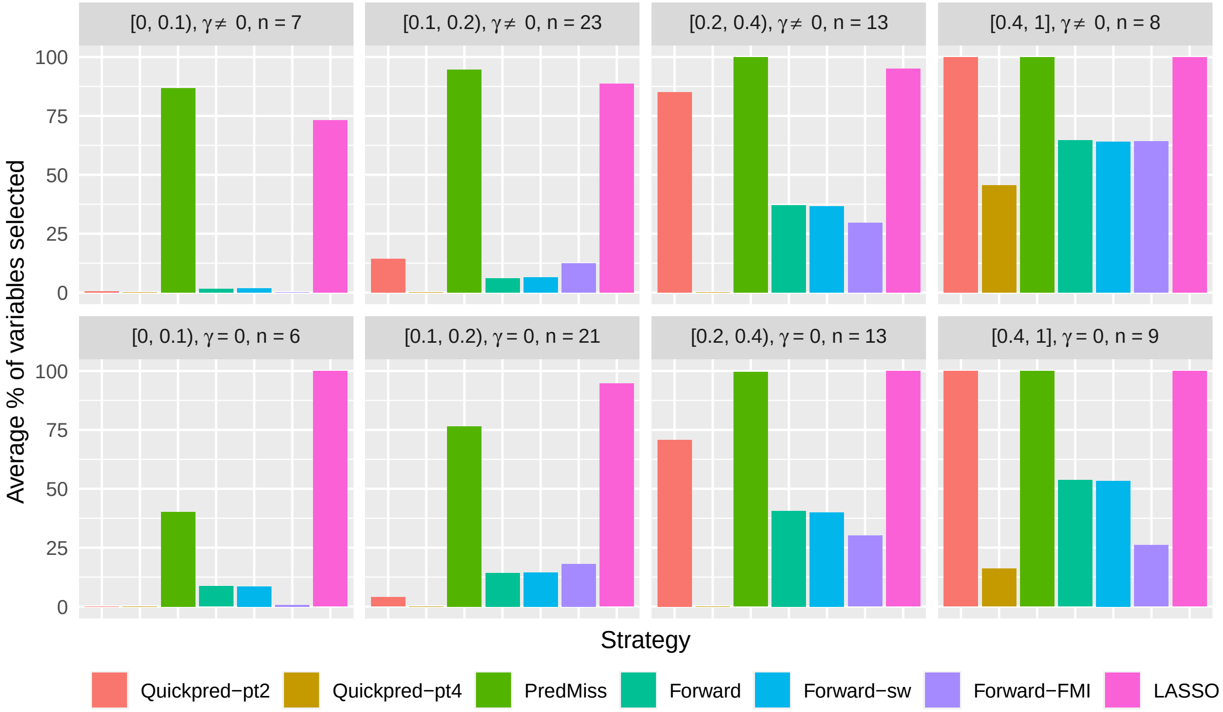

Figure 15: Average number of auxiliary variables selected across simulation runs, expressed as a proportion of the number of auxiliary variables, stratified by auxiliary variable group, for the scenario where $n = 1000$, $p = 100$, $Pr(M_Y = 1) = 0.3$ and the coefficients of X, Z and $\boldsymbol{A}$ in the missingness model are equal to $\log(1.2)$.

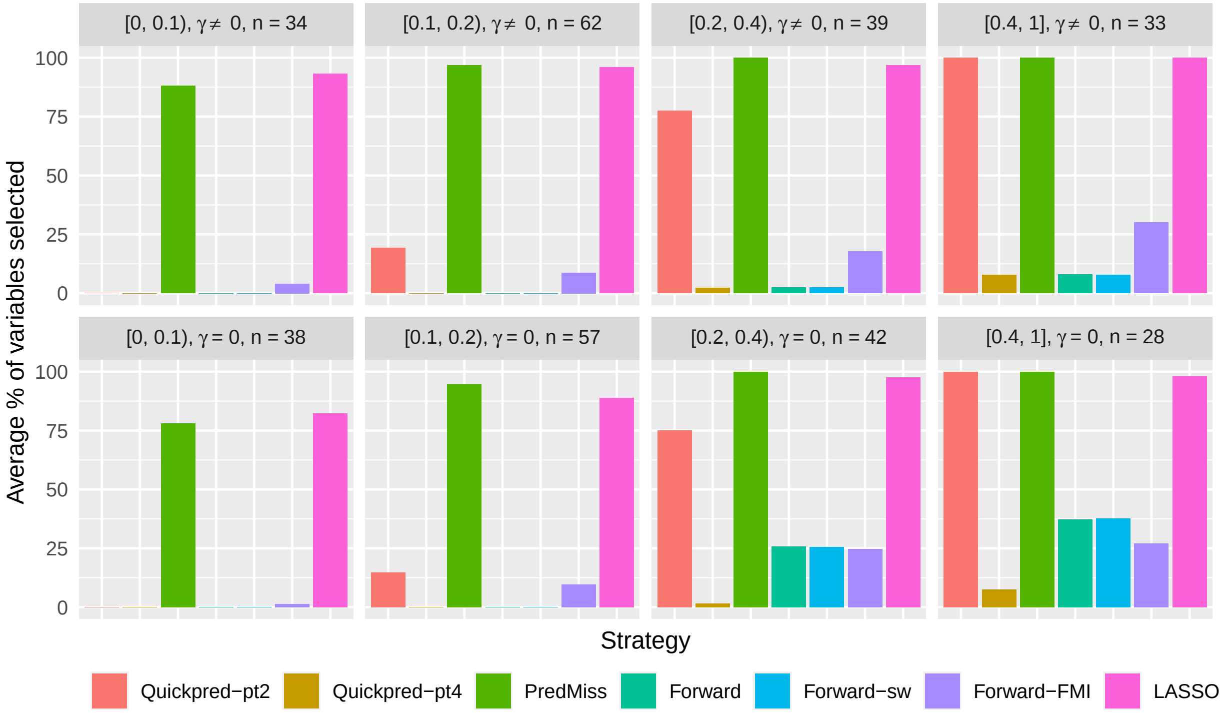

Figure 16: Average number of auxiliary variables selected across simulation runs, expressed as a proportion of the number of auxiliary variables, stratified by auxiliary variable group, for the scenario where $n = 1000$ and $p = 333$.

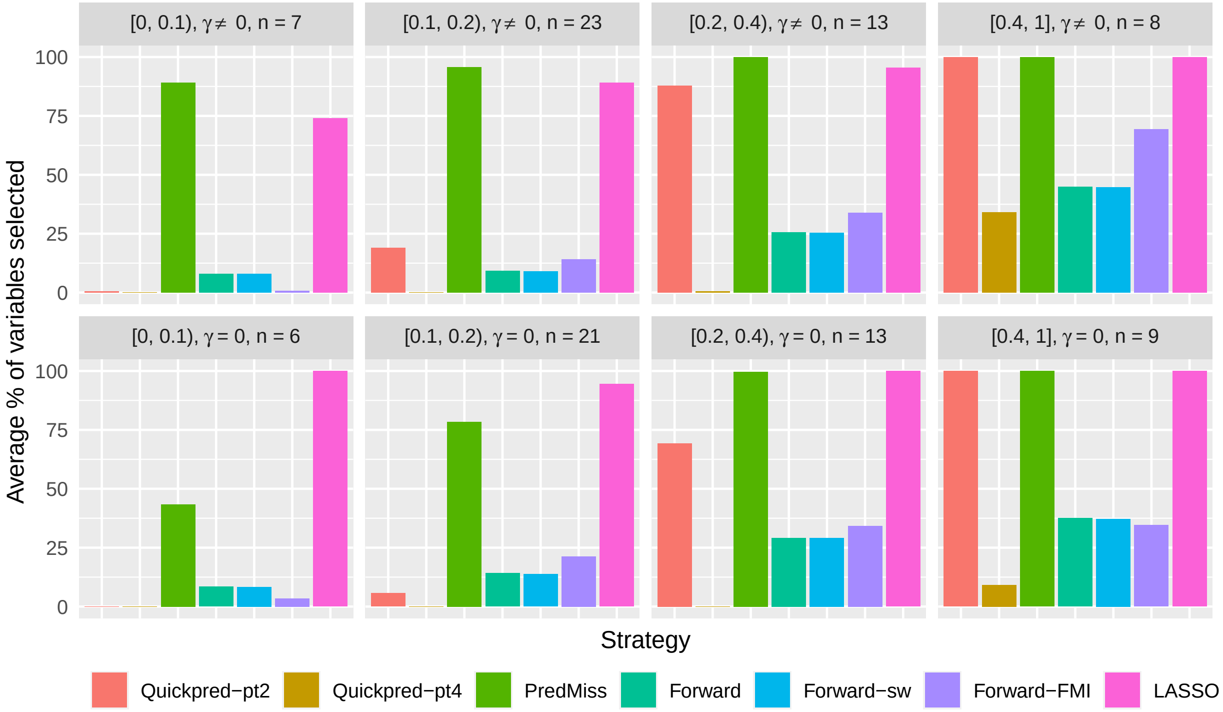

Figure 17: Average number of auxiliary variables selected across simulation runs, expressed as a proportion of the number of auxiliary variables, stratified by auxiliary variable group, for the scenario where $n = 1000$, $p = 100$, $Pr(M_Y = 1) = 0.5$ and the coefficients of X, Z and $\boldsymbol{A}$ in the missingness model are equal to $\log(1.2)$.

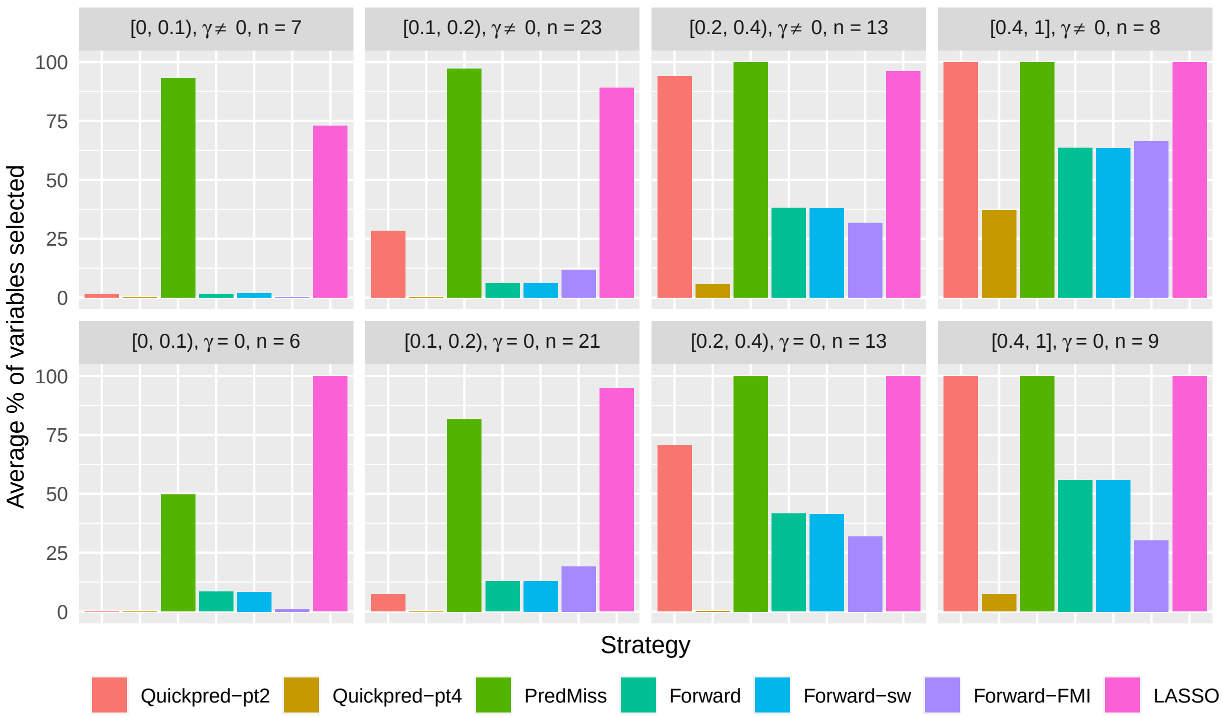

Figure 18: Average number of auxiliary variables selected across simulation runs, expressed as a proportion of the number of auxiliary variables, stratified by auxiliary variable group, for the scenario where $n = 1000$, $p = 100$, $Pr(M_Y = 1) = 0.3$ and the coefficients of X, Z and $\boldsymbol{A}$ in the missingness model are equal to $\log(2)$.

References

[1] J. Hardin, S. R. Garcia, and D. Golan, “A method for generating realistic correlation matrices,” *The Annals of Applied Statistics*, pp. 1733–1762, 2013.

[2] R. Andridge and K. J. Thompson, “Using the fraction of missing information to identify auxiliary variables for imputation procedures via proxy pattern-mixture models,” *Int Stat Rev.*, vol. 83, no. 3, pp. 472–492, 2015.

[3] R. J. Little, “A class of pattern-mixture models for normal incomplete data,” *Biometrika*, vol. 81, no. 3, pp. 471–483, 1994.
